# Supplementary material for: Shared neutrophil and T cell dysfunction is accompanied by a distinct interferon signature during severe febrile illnesses in children
Source: Nat Commun. 2024 Sep 19;15:8224. doi: 10.1038/s41467-024-52246-0 (PMC11413185; doi:10.1038/s41467-024-52246-0)
Supplement: Supplementary file 1 — Supplementary Information [file 41467_2024_52246_MOESM1_ESM.pdf]

SUPPLEMENTARY INFORMATION

Supplementary Table 1: Demographic features and COVID-19 vaccination details of healthy adult controls

Supplementary Table 2: Clinical, demographic and laboratory features of children included in the Transcriptomic Cohort.

Supplementary Table 3: Clinical characteristics of patients included in the mass cytometry experiments.

Supplementary Table 4. Clinical characteristics of children within Cluster 1 and Cluster 2 as defined my immunophenotyping using mass cytometry data.

Supplementary Table 5. Summary of supernatant cytokine analysis following cell stimulation experiments.

Supplementary Table 6. Clinical characteristics of patients and controls included in the cell stimulation mass cytometry experiment.

Supplementary Table 7. Strobe Checklist

Supplementary Figure 1. Duration of prior fever, admission to wards and paediatric intensive care units (PICU) and convalescence relative to sampling at T1, T2 and T3 for individual patients grouped by their severe febrile illness (all patients recruited to the study).

Supplementary Figure 2. Upset plot visualizing counts of patients/controls across different assays.

Supplementary Figure 3. Mass cytometry methods

Supplementary Figure 4. Immune cell populations and expression of markers by mass cytometry. a, Heatmaps displaying hierarchical clustering of immune cell populations from manually gated mass cytometry data in children with acute (time point 1, T1) multisystem inflammatory syndrome in children (MIS-C, n=35), severe bacterial infection (SBI, n=17), severe viral infection (SVI, n=10), Kawasaki disease (KD, n=4) and healthy paediatric controls (HPC, n=10). **b,** Heatmaps displaying hierarchical clustering of immune cell populations from manually gated mass cytometry data in children with MIS-C at time point 1 (T1, acute febrile within 48 hours of admission, n=35), time point 2 (T2, defervescence with CRP concentration <100 mg/l, n=16) and timepoint 3 (T3, convalescence at follow-up clinic, n=26) and healthy paediatric controls (n=10). **c,** Heatmaps displaying hierarchical clustering of immune cell populations from manually gated mass cytometry data in children with severe bacterial infection (SBI) at time point 1 (T1, acute febrile within 48 hours of admission, n=17), time point 2 (T2, defervescence with CRP concentration <100 mg/l, n=8) and timepoint 3 (T3, convalescence at follow-up clinic, n=6) and healthy paediatric controls (n=10).

Supplementary Figure 5. Hierarchical clustering for children with no immunomodulation at T1 sampling.

Supplementary Figure 6. MOFA of immune features from immunophenotyping shows differences between disease groups and key factors of protein expression and cell proportion features in acute (T1) disease and over the trajectory of illness.

Supplementary Figure 7. Factors 1-3 by disease groups and timepoints.

Supplementary Figure 8. Markers of immune cell activation and return to baseline in innate immune cells (mature neutrophils, immature neutrophils, monocytes, myeloid DCs, plasmacytoid DCs, CD4 memory T cells, CD8 memory T cells, memory regulatory T cells). For all figures, patients with MIS-C are represented in shades of purple for T1–T3 and patients with SBI are represented with shades of orange for T1–T3. P values for pairwise-comparisons across time are using Wilcoxon rank sum testing.

Supplementary Figure 9. a, Markers of T cell activation, exhaustion and apoptosis in memory CD4 T cells. **b,** Markers of T cell activation, exhaustion and apoptosis in memory CD8 T cells. **c,** Markers of T cell activation, exhaustion and apoptosis in memory regulatory T cells.

Supplementary Figure 10. Co-expression of CD279 PD1 with markers of activation, exhaustion and apoptosis in CD4 memory T cells. Representative examples of CD279 PD-1 expression (x axis, all plots) versus **a,** HLA-DR; **b,** CD38; **c,** CD152 CTLA4; **d,** CD134 OX40, **e,** CD278 ICOS, **f,** CD95 Fas, **g,** intracellular IFNγ in memory CD4 T cells and naïve/effector CD4 T cells.

Supplementary Figure 11. K-means clustering analysis: effects of immunomodulatory treatment on supernatant cytokines.

Supplementary Figure 12. Supernatant cytokine analysis 1**:** interferons.

Supplementary Figure 13. Supernatant cytokine analysis 2**:** remaining cytokines.

Supplementary Figure 14. Supernatant cytokine sub-analysis of MIS-C T1 by clusters identified on mass cytometry.

Supplementary Figure 15. Heatmap summarising mass cytometry results of stimulated samples.

Supplementary Figure 16. Gene set enrichment analysis (GSEA) results. **a,** children with MIS-C (TP1) versus pediatric healthy controls; **b,** children with definite bacterial infection (TP1) versus pediatric healthy controls; **c,** children with definite viral infection (TP1) versus pediatric healthy controls; **d,** children with MIS-C versus children with definite bacterial infection. GSEA was undertaken in the R statistical environment, using pathways annotated by the Gene Ontology (GO) database.

DIAMONDS Consortium Membership

Supplementary Table 1: Demographic features and COVID-19 vaccination details of healthy adult controls

**Participant ID**	**Sex**	**Age (years)**	**Self-reported ethnicity**	**Date of sample**	**COVID-19 vaccine received**	**Date of vaccination**	**Dose of last vaccine prior to sampling**
Healthy adult control 1	Male	> 21	North/Mid/East European	06/07/2021	mRNA Vaccine BNT162b2	10/03/2021	Second
Healthy adult control 2	Female	> 21	North/Mid/East European	06/07/2021	mRNA Vaccine BNT162b3	21/04/2021	Second
Healthy adult control 3	Female	> 21	North/Mid/East European	06/07/2021	mRNA Vaccine BNT162b4	04/03/2021	Second
Healthy adult control 4	Female	> 21	South Asian	06/07/2021	mRNA Vaccine BNT162b5	07/04/2021	Second
Healthy adult control 5	Female	> 21	North/Mid/East European	13/07/2021	mRNA Vaccine BNT162b6	13/04/2021	Second
Healthy adult control 6	Male	> 21	North/Mid/East European	13/07/2021	mRNA Vaccine BNT162b7	16/03/2021	Second
Healthy adult control 7	Male	> 21	North/Mid/East European	13/07/2021	Non-replicating viral vector vaccine AZD1222 (ChAdOx1)	20/05/2021	Second
Healthy adult control 8	Male	> 21	African - other	13/07/2021	mRNA Vaccine BNT162b4	31/03/2021	Second
Healthy adult control 9	Female	> 21	North/Mid/East European	13/07/2021	mRNA Vaccine BNT162b4	01/07/2021	Second
Healthy adult control 10	Female	> 21	North/Mid/East European	14/07/2021	mRNA Vaccine BNT162b4	20/03/2021	Second
Healthy adult control 11	Male	> 21	South American - other	14/07/2021	mRNA Vaccine BNT162b4	12/05/2021	Second
Healthy adult control 12	Male	> 21	North/Mid/East European	15/07/2021	mRNA Vaccine BNT162b4		
Healthy adult control 13	Male	> 21	North/Mid/East European	22/07/2021	mRNA Vaccine BNT162b4	10/03/2021	Second
Healthy adult control 14	Male	> 21	North/Mid/East European	22/07/2021	Non-replicating viral vector vaccine AZD1222 (ChAdOx1)	24/06/2021	Second
Healthy adult control 15	Female	> 21	North/Mid/East European	03/02/2021	mRNA Vaccine BNT162b4	04/01/2021	First
Healthy adult control 16	Female	> 21	North/Mid/East European	05/02/2021	mRNA Vaccine BNT162b4	01/01/2021	First
Healthy adult control 17	Female	> 21	East Asian	06/10/2021	mRNA Vaccine BNT162b4	19/03/2021	Second
Healthy adult control 18	Female	> 21	North/Mid/East European	06/10/2021	mRNA Vaccine BNT162b4	22/03/2021	Second
Healthy adult control 19	Male	> 21	South American - other	06/10/2021	mRNA Vaccine BNT162b4	26/08/2021	Second
Healthy adult control 20	Female	> 21	North/Mid/East European	06/10/2021	mRNA Vaccine BNT162b4	10/04/2021	Second
Healthy adult control 21	Female	> 21	North/Mid/East European	26/11/2021	mRNA Vaccine BNT162b4	07/10/2021	Booster
Healthy adult control 22	Male	> 21	North/Mid/East European	26/11/2021	mRNA Vaccine BNT162b4	02/10/2021	Booster
Healthy adult control 23	Male	> 21	South Asian	26/11/2021	mRNA Vaccine BNT162b4	04/05/2021	Second
Healthy adult control 24	Female	> 21	East Asian	01/12/2021	mRNA Vaccine BNT162b4	17/11/2021	Booster
Healthy adult control 25	Male	> 21	North/Mid/East European	01/12/2021	mRNA Vaccine BNT162b4	03/04/2021	First
Healthy adult control 26	Male	> 21	Middle Eastern - Arab	01/12/2021	mRNA Vaccine BNT162b4	11/07/2021	Second
Healthy adult control 27	Male	> 21	North/Mid/East European	01/12/2021	mRNA Vaccine BNT162b4	> 14 days prior to sampling	Second

Supplementary Table 2: Clinical, demographic and laboratory features of children included in the Transcriptomic Cohort. Healthy controls were used for normalisation of the data but not for differential expression analysis or feature selection.

	MIS-C (n=38)	***KD (n=136)***	***Viral (n=138)***	***Bacterial (n=188)***	***Healthy controls (n=134)***
**Age in months**	126 (65-151)	30 (16-52)	7 (2-20)	38 (11-96)	92 (29 – 154)
**Sex at birth (female, %)**	13 (39%)	59 (43%)	45 (33%)	85 (45%)	63 (47%)
**Days since symptom onset**	6 (5-7)	6 (5-8)	5 (2-6)	2 (1-4)	-
**Required inotropic support (n, %)**	20 (52.6%)	2 (1.4%)	3 (2%)	50 (26.6%)	-
**Required non-invasive ventilation (n, %)**	5 (13.2%)	2 (1.4%)	6 (4.3%)	46 (24%)	-
**Required invasive ventilation (n, %)**	4 (10.5%)	0	7 (5.1%)	16 (8.5%)	-
**Admitted to PICU (n, %)**	23 (60.6%)	1 (0.7%)	25 (18%)	84 (44.7%)	-
**White blood cells (10^9/L)**	9.7 (8.1-11.5)	13.2 (10.4-18)	9.9 (6-14)	13.2 (8.1-20.6)	-
**Neutrophils (10^9/L)**	8.1 (4.5-11.0)	9.2 (6.3-11.5)	3.6 (1.8-6.5)	14.1 (6-47)	-
**Lymphocytes (10^9/L)**	1.2 (0.8-1.8)	2.8 (1.7-4.9)	4.7 (2.8-6.7)	2.8 (1.5-8.6)	-
**Monocytes (10^9/L)**	0.3 (0.2-0.4)	0.7 (0.5-1.1)	0.8 (0.5-1.4)	1.6 (0.7-4.8)	-
**Platelets (10^9/L)**	209 (144-300.5)	343 (257-426)	365 (245-509)	243 (157-329)	-
**CRP (mg/L)**	173 (99-250)	73 (43-162)	13.2 (4.1-38)	116.8 (40.3-212)	-

Supplementary Table 3: Clinical characteristics of patients included in the mass cytometry experiment.

**Characteristics**	**MIS-C**	**SBI**	**SVI**	**KD**	**HPC**
n	35	17	10	4	10
Age (years) (median, IQR)	8.0 (2.6–13.3)	6.4 (2.6–13.9)	2.6 (1.4–10.1)	0.8 (0.6–2.1)	6.6 (5.1–10)
Female (n, %)	14 (40%)	5 (31%)	7 (70%)	1 (25%)	4 (40%)
Self-reported ethnicity					
Asian (n, %)	5 (15%)	1 (5.9%)	1 (10%)	1 (25%)	0
Black (n, %)	8 (24%)	1 (5.9%)	2 (20%)	0	2 (20%)
Other (n, %)	9 (26%)	1 (5.9%)	0	1 (25%)	1 (10%)
White (n, %)	12 (35%)	14 (82%)	7 (70%)	2 (50%)	7 (70%)
Comorbidity[1]	4 (11%)	7 (41%)	1 (10%)	1 (25%)	0
Presenting clinical features					
Gastrointestinal symptoms	27 (77%)	12 (71%)	3 (30%)	3 (75%)	–
Oral mucositis	4 (11%)	0	0	2 (50%)	–
Rash	23 (66%)	5 (29%)	2 (20%)	4 (100%)	–
Respiratory symptoms	8 (23%)	12 (71%)	7 (70%)	0	–
Additional findings during admission					
Maximum CRP concn (mg/l) (median, IQR)	200 (143–270)	197 (168–299)	129 (67–150)	111 (89–147)	–
Maximum neutrophil count (x10^9/l) (median, IQR)	13 (9.8–18)	15 (9.0–20)	8.8 (7.2–13)	14 (11–16)	–
Minimum lymphocyte count (x10^9/l) (median, IQR)	0.6 (0.4–1.2)	1.3 (0.6–2.9)	1.0 (0.7–2.0)	2.4 (1.7–3.2)	–
Coronary artery aneurysm (n, %)	5 (14%)	0	0	1 (25%)	–
Treatments during admission					
Mechanical ventilation (n, %)	3 (8.8%)	8 (47%)	8 (80%)	0	–
Vasoactive infusion (n, %)	32 (94%)	9 (56%)	3 (3.3%)	0	–
Intravenous immunoglobulin	12 (34%)	2 (12%)	0	4 (100%)	–
High dose corticosteroids	33 (94%)	1 (5.8%)	0	3 (75%)	–
Monoclonal antibody therapy[2]	8 (23%)	0	0	0	–
Outcome					
PICU admission (n, %)	34 (97%)	16 (94%)	9 (90%)	0	–
Hospital length of stay (days) (median, IQR)	6.7 (5.5–8.2)	8.7 (7.8–17)	14 (7.4–28)	6.7 (4.9–8.6)	–
In-hospital mortality (n, %)	1 (2.9%)	1 (5.9%)	1 (10%)	0	–
Infections identified					
SARS-CoV-2 PCR/antigen positive (n, %)	1 (2.9%)	0	3 (30%)	0	–
SARS-CoV-2 IgG antibody positive (n, %)	33 (94%)	0	3 (30%)	0	–

[1]MIS-C: Minor neurological co-morbidities 2, respiratory 1, endocrinological 1; SBI: respiratory 2, cardiac 1, endocrinological 1, minor neurological 2, other genetic syndrome 1; SVI: other genetic syndrome 1; KD: renal 1 child. [2]MIS-C: tocilizumab in 8 children.

Supplementary Table 4. Clinical characteristics of children within Cluster 1 and Cluster 2 as defined by immunophenotyping using mass cytometry data. Statistical comparisons between Cluster 1 and Cluster 2 were made using the two-sided Fisher exact test of proportions or two-sided Wilcoxon test with no adjustments for multiple comparisons.

	**Cluster 1**	**Cluster 2**	**p**
n	29 (44%)	37 (56%)	
Disease group			
MIS-C	22 (76%)	13 (35%)	0.001
SBI	4 (14%)	13 (35%)	0.087
SVI	3 (10%)	7 (19%)	0.493
KD	0	4 (11%)	0.125
Age (years) (median, IQR)	9.3 (4.9–14)	5.4 (1.6–8.9)	<0.001
Female (n, %)	12 (41%)	15 (41%)	1
Self-reported ethnicity			
Asian (n, %)	1 (3%)	2 (5%)	1
Black (n, %)	5 (17%)	5 (14%)	0.738
Other (n, %)	10 (34%)	9 (24%)	0.419
White (n, %)	13 (45%)	21 (57%)	0.457
Additional findings during admission			
Maximum CRP concentration (mg/l) (median, IQR)	209 (134–288)	179 (124–220)	0.392
Treatments during admission			
Mechanical ventilation (n, %)	7 (24%)	9 (24%)	1
Vasoactive infusion (n, %)	19 (66%)	15 (41%)	0.051
Intravenous immunoglobulin (pre-T1 sampling)	5 (17%)	5 (14%)	0.738
High dose corticosteroids (pre-T1 sampling)	12 (41%)	8 (22%)	0.108
Monoclonal antibody therapy[2] (pre-T1 sampling)	0	1 (3%)	1
Outcome			
PICU admission (n, %)	23 (79%)	25 (68%)	0.405
Hospital length of stay (days) (median, IQR)	6.8 (6.2–7.9)	8.1 (5.5–10)	0.413

Supplementary Table 5. Summary of supernatant cytokine analysis following cell stimulation experiments. Median values by cohort and timepoint for baseline results and percentage change from baseline following stimulation with SARS-CoV-2 antigen and mitogen (phytohemagglutinin) are shown. Patients with Multisystem Inflammatory Syndrome in Children (MIS-C), Severe bacterial illness (SBI), Kawasaki disease (KD), Severe viral illness (SVI) and other inflammatory conditions at acute (TP1) and convalescence (TP3), as well as healthy paediatric controls and healthy COVID-19 vaccinated adults were included. The number of samples are indicated in the table for each result. Units are pg/ml for all cytokines are derived from a standard curve for each assay. Abbreviations: IL, interleukin; TNF, tumour necrosis factor; IP, interferonγ induced protein, GMCSF, granulocyte-macrophage colony stimulating factor; IFN, interferon.

Colour	Legend
(red)	Highest value
(yellow)	50th Percentile
(blue)	Lowest value

	BASELINE - median results for each cohort/timepoint										
**Disease group and timepoint**	MIS-C - TP1	MIS-C - TP3	SBI - TP1	SBI - TP3	KD - TP1	KD - TP3	SVI - TP1	SVI - TP3	Inflammatory - TP1	HPC	HAC
**IL1b**	42	70	86	134	NA	158	65	8	69	54	64
**IL6**	103	55	164	179	NA	187	83	149	389	86	319
**TNFα**	24	54	35	208	NA	118	41	1	44	33	44
**IP10**	948	101	256	78	27	143	757	1071	190	79	167
**IFNλ1**	82	170	123	97	1000000	240	229	10	264	119	80
**IL8**	541	2384	3314	3365	7386	3359	1494	210	5469	1769	4512
**IL12p70**	2	8	4	4	4	4	2	0	3	3	2
**IFNα2**	9	16	14	14	23	34	11	4	14	11	3
**IFNλ2/3**	232	175	292	130	215	385	252	60	226	213	210
**GMCSF**	6	35	20	24	18	9	12	0	10	8	0
**IFNβ**	14	0	27	10	0	43	7	3	14	0	0
**IL20**	32	11	23	7	28	6	31	157	14	11	5
**IFNγ**	169	307	156	89	88	413	215	114	110	122	29
**Number of samples**	41	15	13	3	1	3	7	1	9	15	25
SARS-CoV-2 AG STIMULATION - median percentage change from baseline for each cohort/timepoint											
**Disease group and timepoint**	MIS-C - TP1	MIS-C - TP3	SBI - TP1	SBI - TP3	KD - TP1	KD - TP3	SVI - TP1	SVI - TP3	Inflammatory - TP1	HPC	HAC
**IL1b**	8%	22%	8%	-22%	NA	-9%	-22%	15%	-2%	2%	28%
**IL6**	-4%	35%	3%	-43%	NA	79%	5%	-25%	0%	-2%	45%
**TNFα**	-16%	19%	25%	-18%	NA	125%	49%	-42%	-12%	-26%	66%
**IP10**	0%	60%	1%	5%	107%	19%	-7%	9%	3%	26%	427%
**IFNλ1**	1%	-6%	5%	633305%	-100%	29%	-51%	-100%	-22%	6%	7%
**IL8**	51%	36%	40%	68%	97%	37%	67%	89%	21%	67%	78%
**IL12p70**	6%	3%	14%	6%	14%	-1%	-10%	-24%	-14%	-2%	9%
**IFNα2**	7%	4%	6%	-7%	14%	12%	-1%	-50%	-24%	9%	15%
**IFNλ2/3**	-2%	15%	6%	-25%	24%	26%	-40%	-33%	-32%	18%	10%
**GMCSF**	-10%	6%	14%	-31%	22%	44%	-7%	-100%	-32%	2%	32%
**IFNβ**	-10%	12%	-12%	28%	3771%	89%	-87%	-100%	-61%	-41%	141%
**IL20**	-2%	27%	7%	-23%	3%	24%	12%	-22%	-7%	-11%	56%
**IFNγ**	8%	47%	26%	4%	20%	36%	2%	-68%	-13%	25%	246%
**Number of samples**	35	15	13	3	1	3	6	1	9	14	22
MITOGEN STIMULATION - median percentage change from baseline for each cohort/timepoint											
**Disease group and timepoint**	MIS-C - TP1	MIS-C - TP3	SBI - TP1	SBI - TP3	KD - TP1	KD - TP3	SVI - TP1	SVI - TP3	Inflammatory - TP1	HPC	HAC
**IL1b**	85%	346%	17%	649%	NA	573%	210%	96%	15%	402%	692%
**IL6**	114%	6492%	64%	1772%	NA	7063%	911%	554%	-1%	8673%	3102%
**TNFα**	107%	268%	87%	959%	NA	920%	349%	85%	46%	3266%	2924%
**IP10**	18%	1331%	38%	2116%	568%	2172%	99%	1510%	20%	2178%	1125%
**IFNλ1**	7%	18%	7%	52%	-100%	220%	14%	15%	70%	83%	24%
**IL8**	495%	419%	142%	450%	249%	144%	620%	230%	17%	757%	355%
**IL12p70**	15%	27%	8%	32%	65%	145%	32%	54%	-35%	51%	211%
**IFNα2**	3%	36%	10%	-19%	54%	31%	20%	61%	-15%	43%	47%
**IFNλ2/3**	-7%	29%	1%	-10%	57%	44%	11%	9%	22%	20%	36%
**GMCSF**	32%	183%	24%	1561%	98%	566%	98%	122%	33%	1591%	16706%
**IFNβ**	13%	59%	7%	130%	9047%	1626%	360%	187%	-31%	101%	289%
**IL20**	24%	1342%	41%	5137%	24%	3122%	242%	104%	2%	2464%	10328%
**IFNγ**	139%	2025%	265%	23720%	306%	5776%	803%	1168%	36%	6683%	91444%
**Number of samples**	35	15	13	3	1	3	6	1	9	14	22

Supplementary Table 6. Clinical characteristics of patients and controls included in the cell stimulation mass cytometry experiment. Abbreviations: HPC, healthy pediatric controls.

	**MIS-C (N = 12)**	**HPCs (N = 3)**
Age (years), median [IQR]	6.0 [4.0 - 11]	9.5 [6.0-12]
Male sex, n (%)	8 (66.7%)	2(66.7%)
Ethnicity		
Black	6 (50.0%)	0 (0%)
Asian	2 (16.7%)	0 (0%)
White	3 (25.0%)	3 (100%)
Other/Not known	1 (8.3%)	0 (0%)
Maximum CRP (mg/L), median [IQR]	220 [160 - 300]	-
Maximum Neutrophil count ($x10^9$/L), median [IQR]	14 [11 - 18]	-
Maximum Lymphocyte count ($x10^9$/L), median [IQR]	7.5 [5.0 - 12]	-
IVIG pre-mass cytometry sample, n(%)	5 (41.7%)	-
Steroids pre-mass cytometry sample, n(%)	10 (83.3%)	-
Monoclonal antibodies pre-mass cytometry sample, n(%)	3 (25.0%)	-
ITU, n (%)	10 (83.3%)	-
Shock, n (%)	9 (75.0%)	-
Inotropes, n (%)	9 (75.0%)	-
Invasive ventilation, n (%)	1 (8.3%)	-
Death, n (%)	0 (0%)	-
Length of hospital admission (days), median [IQR]	6.5 [5.0 - 8.0]	-
SARS-CoV-2 PCR positive, n (%)	0 (0%)	-
SARS-CoV-2 Serology positive, n (%)	10 (83%)	-

Supplementary Table 7. Strobe Checklist

STROBE Statement—checklist of items that should be included in reports of observational studies

	Item No.	Recommendation	Page No.	Relevant text from manuscript
**Title and abstract**	1	(*a*) Indicate the study's design with a commonly used term in the title or the abstract	1	
		(*b*) Provide in the abstract an informative and balanced summary of what was done and what was found	4	
**Introduction**				
Background/rationale	2	Explain the scientific background and rationale for the investigation being reported	5–6	
Objectives	3	State specific objectives, including any prespecified hypotheses	5–6	
**Methods**				
Study design	4	Present key elements of study design early in the paper	5–6	
Setting	5	Describe the setting, locations, and relevant dates, including periods of recruitment, exposure, follow-up, and data collection	17–18	
Participants	6	(*a*) *Cohort study*—Give the eligibility criteria, and the sources and methods of selection of participants. Describe methods of follow-up<br>*Case-control study*—Give the eligibility criteria, and the sources and methods of case ascertainment and control selection. Give the rationale for the choice of cases and controls<br>*Cross-sectional study*—Give the eligibility criteria, and the sources and methods of selection of participants	17–18	
		(*b*) *Cohort study*—For matched studies, give matching criteria and number of exposed and unexposed<br>*Case-control study*—For matched studies, give matching criteria and the number of controls per case	17–18 and Suppl. Figure 1	
Variables	7	Clearly define all outcomes, exposures, predictors, potential confounders, and effect modifiers. Give diagnostic criteria, if applicable	Table 1 and Figures	
Data sources/ measurement	8*	For each variable of interest, give sources of data and details of methods of assessment (measurement). Describe comparability of assessment methods if there is more than one group	18–26 and Suppl. Figures 2–4.	

Bias	9	Describe any efforts to address potential sources of bias	5–6, 18–26
Study size	10	Explain how the study size was arrived at	5–6

Quantitative variables	11	Explain how quantitative variables were handled in the analyses. If applicable, describe which groupings were chosen and why	18–26
Statistical methods	12	(*a*) Describe all statistical methods, including those used to control for confounding	18–26
		(*b*) Describe any methods used to examine subgroups and interactions	18–26
		(*c*) Explain how missing data were addressed	18–26
		(*d*) *Cohort study*—If applicable, explain how loss to follow-up was addressed<br>*Case-control study*—If applicable, explain how matching of cases and controls was addressed<br>*Cross-sectional study*—If applicable, describe analytical methods taking account of sampling strategy	Suppl. Figure 1
		(*e*) Describe any sensitivity analyses	N/A
**Results**			
Participants	13*	(a) Report numbers of individuals at each stage of study—eg numbers potentially eligible, examined for eligibility, confirmed eligible, included in the study, completing follow-up, and analysed	Suppl. Figures 1 and 2, Table 1
		(b) Give reasons for non-participation at each stage	N/A
		(c) Consider use of a flow diagram	Suppl. Figure 1
Descriptive data	14*	(a) Give characteristics of study participants (eg demographic, clinical, social) and information on exposures and potential confounders	Table 1
		(b) Indicate number of participants with missing data for each variable of interest	Table 1
		(c) *Cohort study*—Summarise follow-up time (eg, average and total amount)	Suppl. Figure 1
Outcome data	15*	*Cohort study*—Report numbers of outcome events or summary measures over time	Table 1

		Case-control study—Report numbers in each exposure category, or summary measures of exposure	
		Cross-sectional study—Report numbers of outcome events or summary measures	
Main results	16	(*a*) Give unadjusted estimates and, if applicable, confounder-adjusted estimates and their precision (eg, 95% confidence interval). Make clear which confounders were adjusted for and why they were included	Figures 1–5
		(*b*) Report category boundaries when continuous variables were categorized	Figures 1–5
		(*c*) If relevant, consider translating estimates of relative risk into absolute risk for a meaningful time period	

Other analyses	17	Report other analyses done—eg analyses of subgroups and interactions, and sensitivity analyses	Figures 1–5
**Discussion**			
Key results	18	Summarise key results with reference to study objectives	14–16
Limitations	19	Discuss limitations of the study, taking into account sources of potential bias or imprecision. Discuss both direction and magnitude of any potential bias	14–16
Interpretation	20	Give a cautious overall interpretation of results considering objectives, limitations, multiplicity of analyses, results from similar studies, and other relevant evidence	14–16
Generalisability	21	Discuss the generalisability (external validity) of the study results	14–16
**Other information**			
Funding	22	Give the source of funding and the role of the funders for the present study and, if applicable, for the original study on which the present article is based	37

Supplementary Figure 1. Duration of prior fever, admission to wards and paediatric intensive care units (PICU) and convalescence relative to sampling at T1, T2 and T3 for individual patients grouped by their severe febrile illness (all patients recruited to the study).

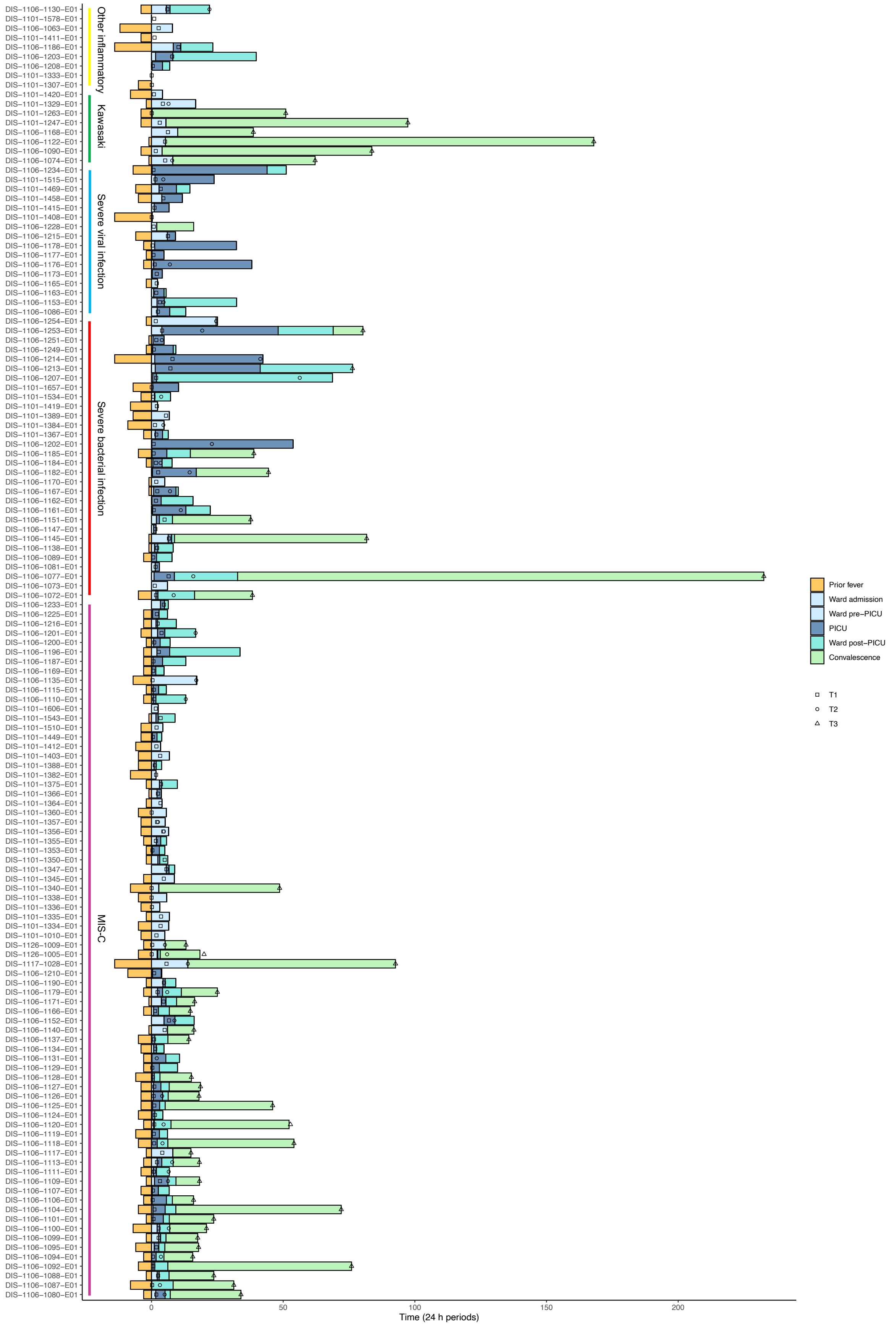

Supplementary Figure 2. Upset plot visualizing counts of patients/controls across different assays. The number of patients and controls analyzed by various combination of assays is shown by vertical bars on the top of the diagrams. The total number of patients analyzed with each assay is indicated by horizontal bars on the right of each panel. Acute (T1), defervescence (T2) and convalescence (T3) are shown. Abbreviations: KD, Kawasaki disease.

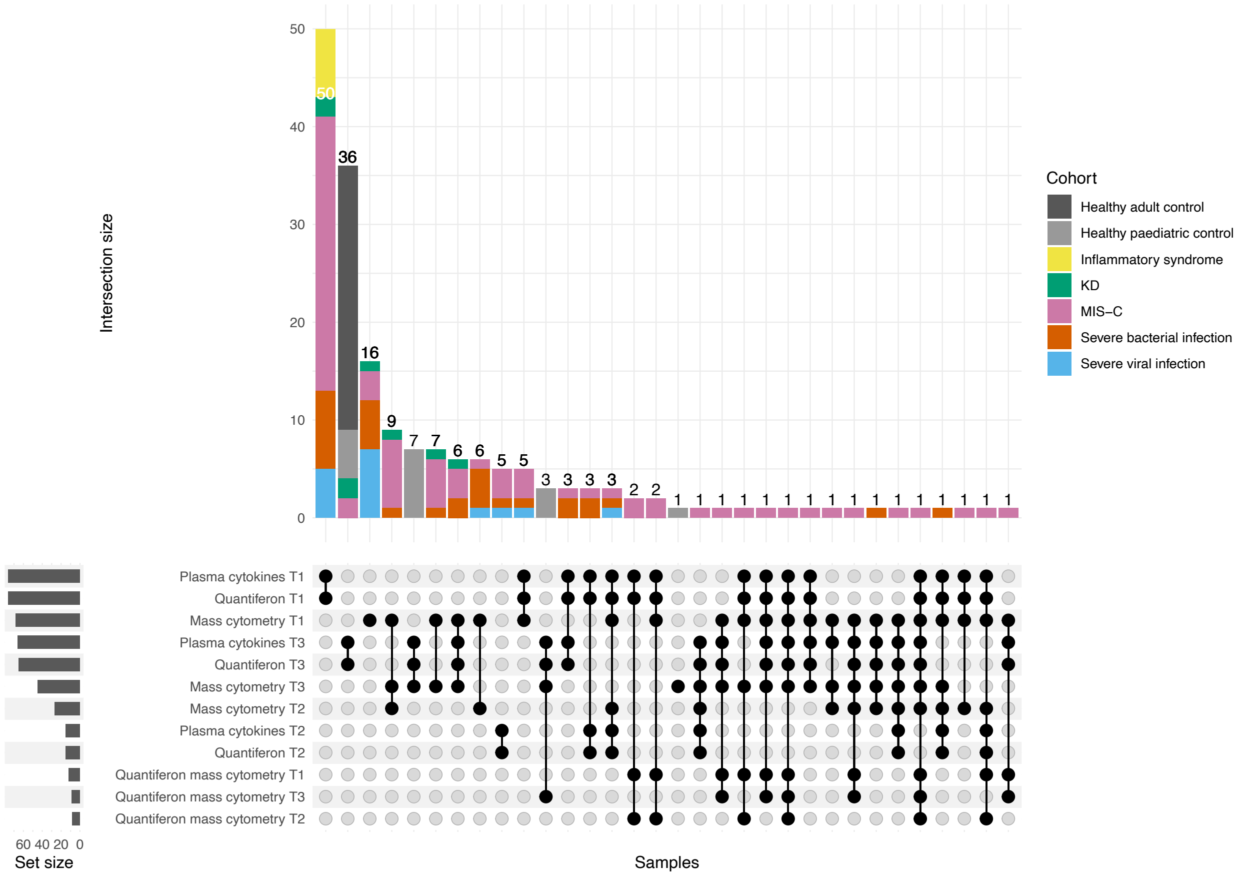

Supplementary Figure 3. Mass cytometry methods. **a–d,** Antibodies used for the mass cytometry methods. Antibodies were from Fluidigm (CA, USA) unless specified. Biolegend (CA, USA) antibodies were custom conjugated to metal tags at the at Guy's and St Thomas' NHS Foundation Trust. Antibodies used for analysis of cell proportions are denoted with an asterisk (also see gating strategy that details this analysis). Note CD8a and CD64 were chelated to the same metal tag but manually gated separately using CD3 as a marker for T cells. **e,** Manual gating strategy, and **f,** implementation of this strategy on a single representative patient (drawn from FlowJo, Beckmann-Coulter based directly on gating in Cytobank, Beckmann-Coulter). **g,** Expression of markers on cell populations from patients with acute MIS-C (other time points and severe febrile illnesses were examined in the same manner).

a, *Barcoding mastermixes*

**Sample 1**	CD45-**106Cd**	
	CD45-**110Cd**	
**Sample 2**	CD45-**106Cd**	
	CD45-**198Pt**	
**Sample 3**	CD45-**106Cd**	
	CD45-**89Y**	
**Sample 4**	CD45-**110Cd**	
	**CD45-198Pt**	
**Sample 5**	CD45-**110Cd**	
	CD45-**89Y**	
**Sample 6**	CD45-**198Pt**	
	CD45-**89Y**	

b, *Cell surface mastermix*

**Cell surface stain**	**Metal Tag**
CD3 UCHT1 (Biolegend)*	111Cd
CD4 RPA-T4 (Biolegend)*	113Cd
CD25 (2A3)*	169Tm
CD127/IL-7Ra (A019D5)*	176Yb
CD45RO (UCHL1)*	149Sm
HLA-DR (G46- 6)*	151Eu
CD197/CCR7 (G043H7)*	167Er
CD45RA (HI100)*	155Gd
CD38 HB-7 HIT2 (Biolegend)*	161Dy
CD8a RPA-T8 (Biolegend)*	116Cd
TCRgd (11F2)*	152Sm
CD19 HIB19 (Biolegend)*	112Cd
CD14 RMO52 M5E2 (Biolegend)*	114Cd
CD15 W6D3 (Biolegend)*	142Nd
CD16 (3G8)*	209Bi
CD20 (2H7)*	171Yb
CD11c (Bu15)*	159Tb
CD27 (L128)*	162Dy
CD5 UCHT2 (Biolegend)	141Pr
CD64 10.1 (Biolegend)	116Cd
CD278/ICOS (C398.4A)	143Nd
CD11b/Mac-1 (ICRF44)	144Nd
CD223/LAG- 3 (11C3C65)	150Nd
CD10 HI10a	156Gd
CD134/OX40 (ACT35)	158Gd
CD95/Fas DX2 (Biolegend)	163Dy
CD161 (HP- 3G10)	164Dy
CD206/MMR (15-2)	168Er
CD279/PD-1 (EH12.2H7)	174Yb
CD28 (CD28.2)	160Gd

c, *Intracellular cytokine mastermix*

**Intracellular cytokines**	**Metal Tag**
IL-6 (MQ2- 13A5)	154Sm
IL-17A (BL168)	148Nd
IFNg (B27)	165Ho
TNFa (MAb11)	146Nd
IL-2 MQ1-17H12 (Biolegend)	145Nd
CD152/CTLA-4 (14D3)	170Er

d, *Phosphorylated marker mastermix*

**Phosphorylated targets**	**Metal Tag**
pStat5 [Y694] (47)	147Sm
pStat1 [Y701] (4a)	153Eu
pS6 [S235/S236] (N7- 548)	175Lu
pNFkBp65 [S529] (K10x)	166Er

e, *Manual gating of cell populations*

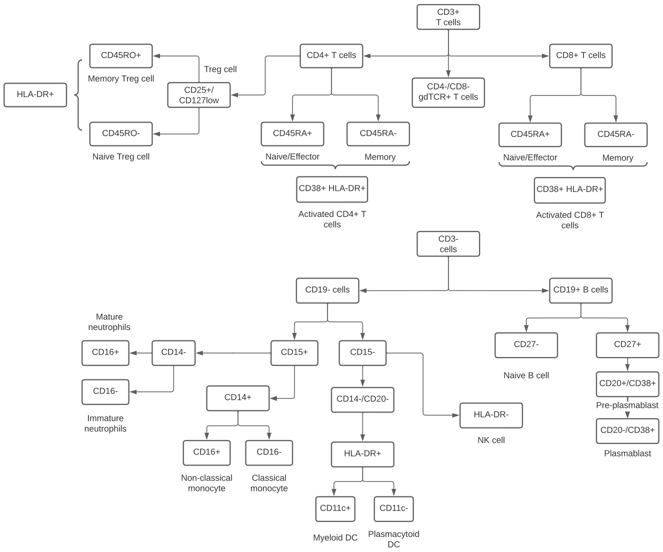

f, *Implementation of manual gating on a single patient with acute MIS-C*

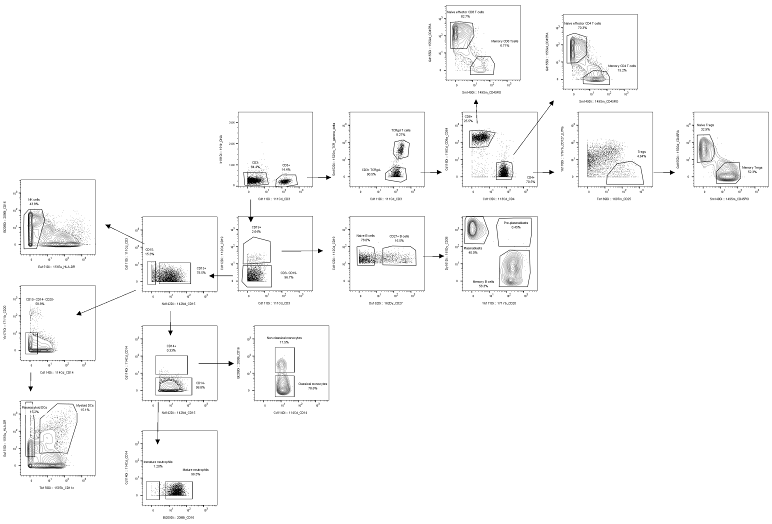

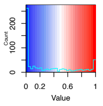

g, *Cell population expression markers in patients with MIS-C*

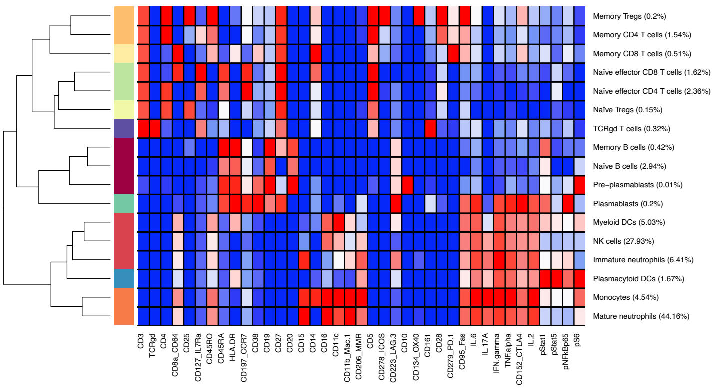

Supplementary Figure 4. Immune cell populations and expression of markers by mass cytometry. a, Heatmaps displaying hierarchical clustering of immune cell populations from manually gated mass cytometry data in children with acute (time point 1, T1) multisystem inflammatory syndrome in children (MIS-C, n=35), severe bacterial infection (SBI, n=17), severe viral infection (SVI, n=10), Kawasaki disease (KD, n=4) and healthy paediatric controls (HPC, n=10), used in the main manuscript. Cells are coloured by the expression of cell markers in an immune cell population, normalised to the expression of that marker across all immune cells analysed. Each heatmap describes a different severe febrile illness at timepoint 1.

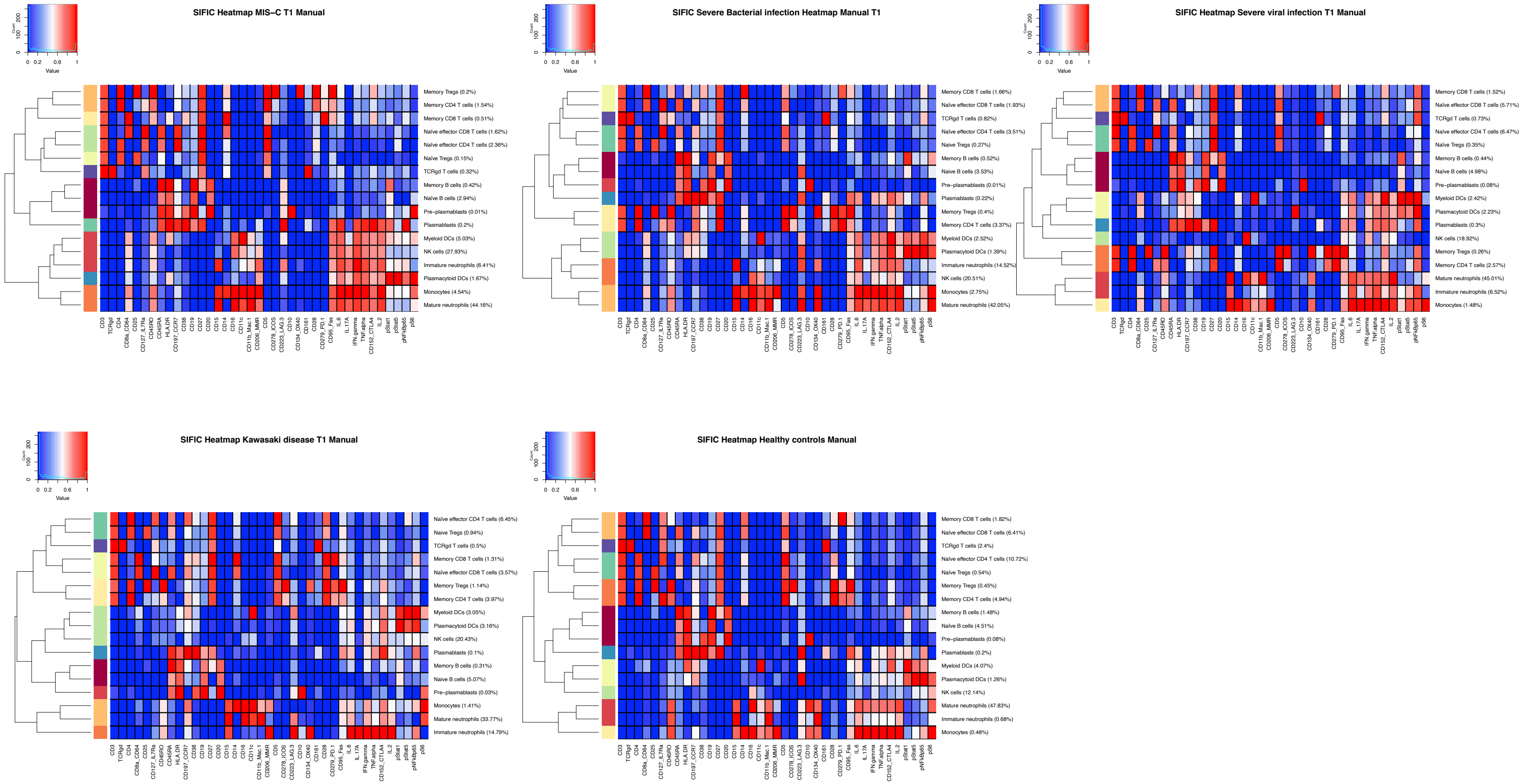

b, Heatmaps displaying hierarchical clustering of immune cell populations from manually gated mass cytometry data in children with MIS-C at time point 1 (T1, acute febrile within 48 hours of admission, n=35), time point 2 (T2, defervescence with CRP concentration <100 mg/l, n=16) and timepoint 3 (T3, convalescence at follow-up clinic, n=26) and healthy paediatric controls (n=10). Cells are coloured by expression as for **a** (above).

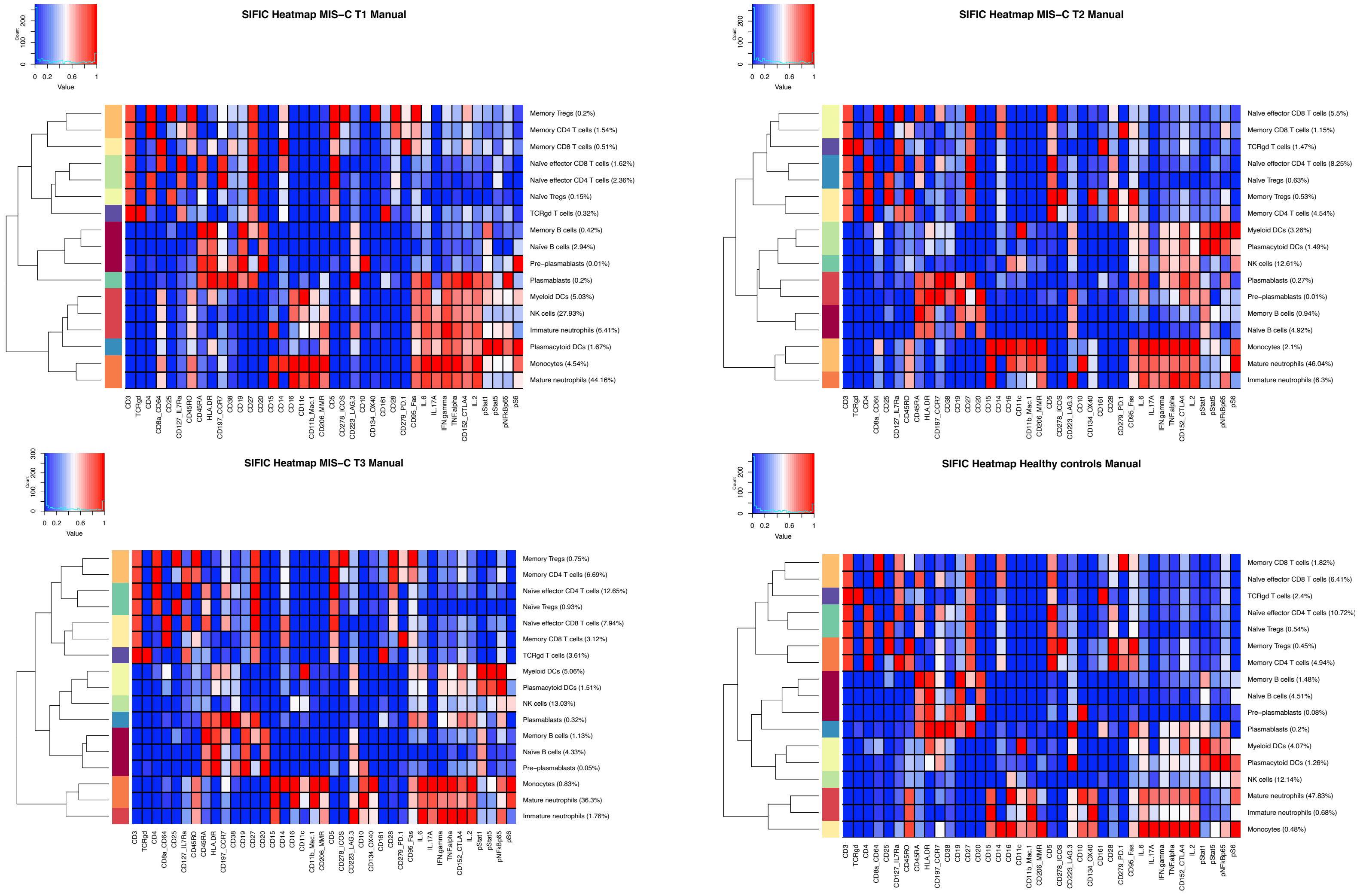

c, Heatmaps displaying hierarchical clustering of immune cell populations from manually gated mass cytometry data in children with severe bacterial infection (SBI) at time point 1 (T1, acute febrile within 48 hours of admission, n=17), time point 2 (T2, defervescence with CRP concentration <100 mg/l, n=8) and timepoint 3 (T3, convalescence at follow-up clinic, n=6) and healthy paediatric controls (n=10). Cells are coloured by expression as for **a** and **b** (above).

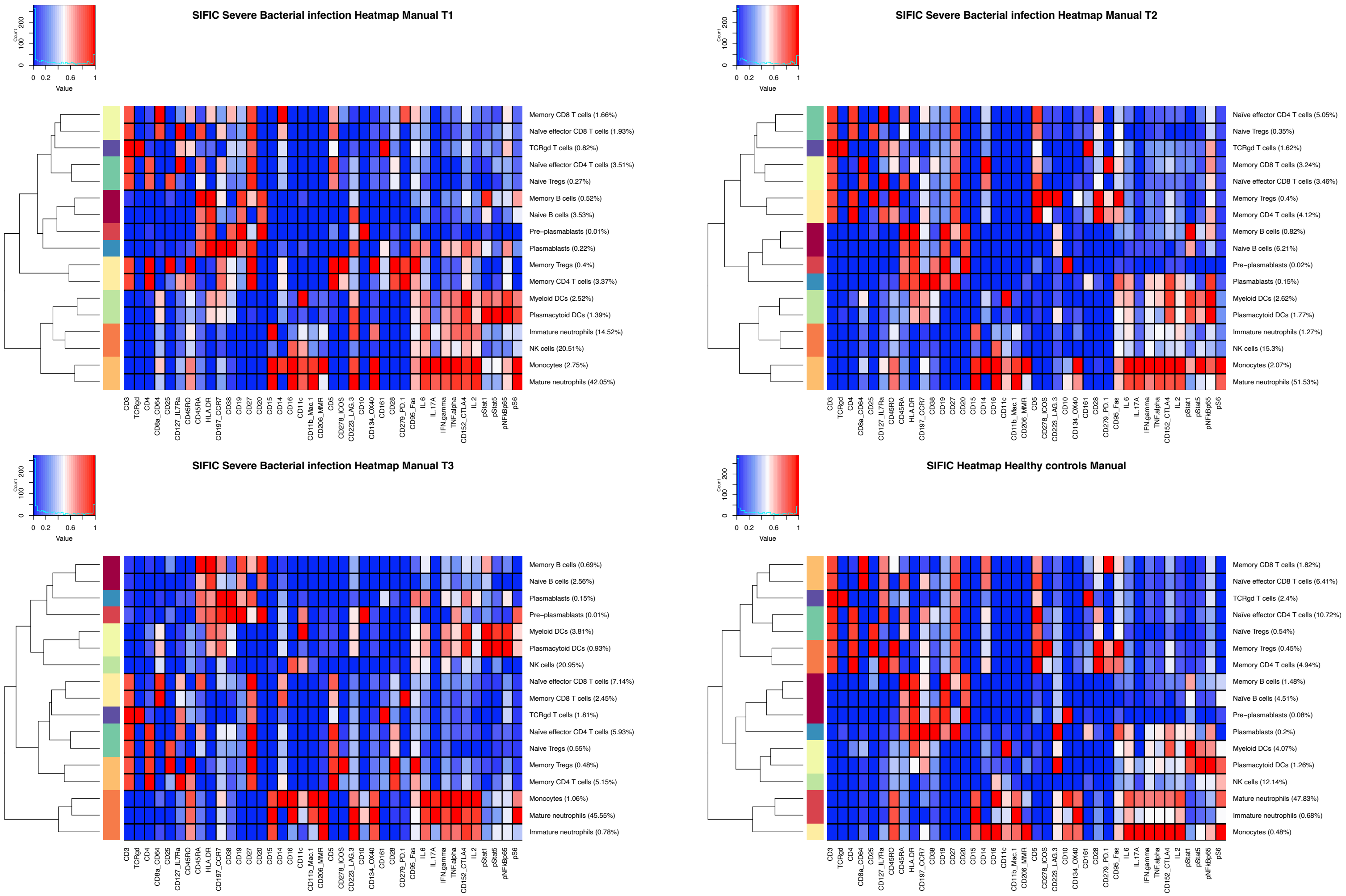

Supplementary Figure 5. Hierarchical clustering for children with no immunomodulation at T1 sampling. Heatmap displaying protein expression and cell proportion data that associated with disease group (**b**) with disease groups clustered as columns (Ward's hierarchical clustering).

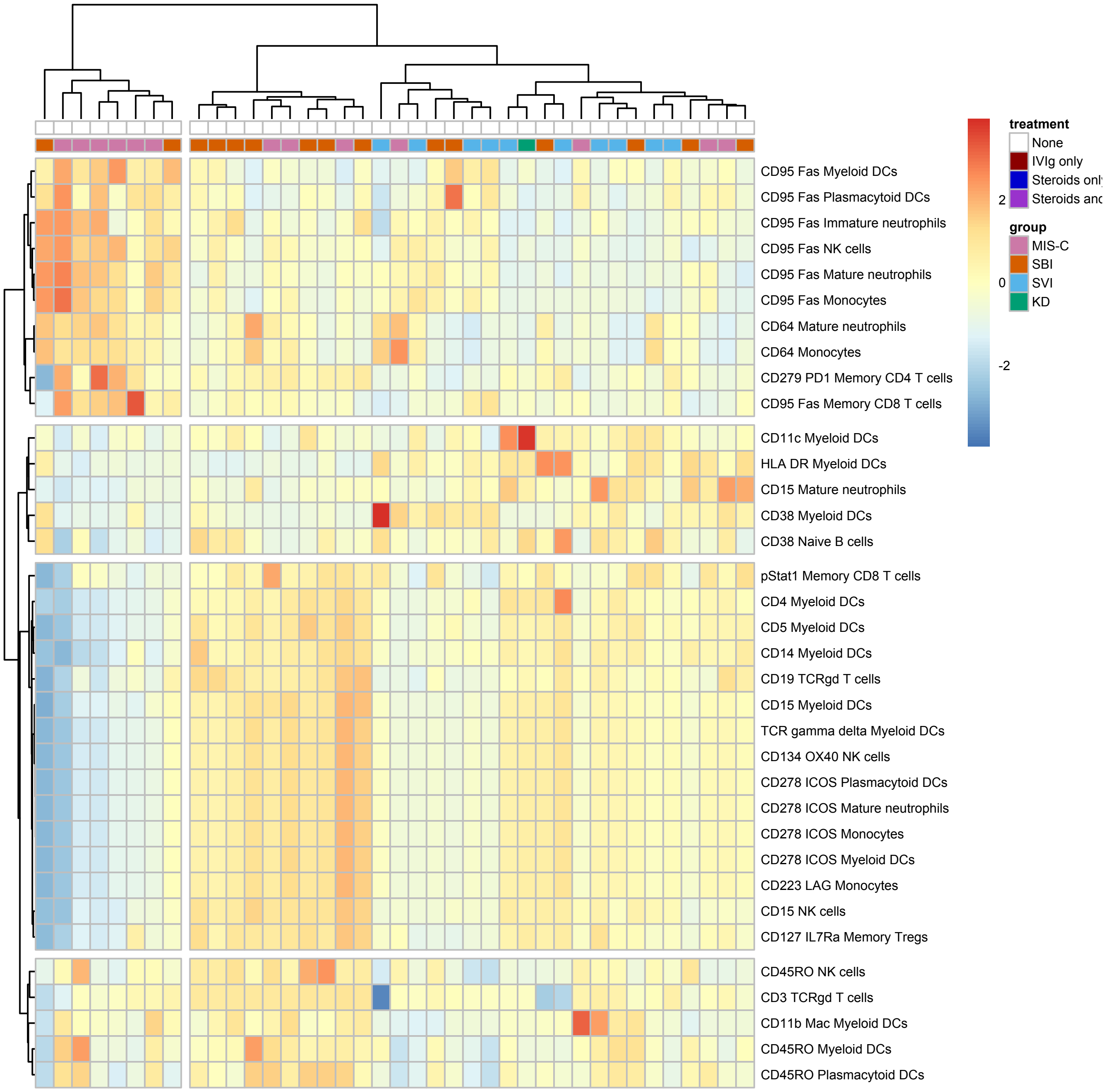

Supplementary Figure 6. MOFA of immune features from immunophenotyping shows differences between disease groups and key factors of protein expression and cell proportion features in acute (T1) disease and over the trajectory of illness. **a,** Variance in data by MOFA Factor, and contribution to the variance by the immune cell proportion data and the cell population expression data. **b,** Relative contribution of features to the top four factors derived from MOFA. **c,** Correlation of Factors to disease group and time point. **d,** Correlation of Factors to Cluster 1 and 2 (from Figure 2c) *$p<0.05$, **$p<0.01$, ***$p<0.001$, ****$p<0.0001$. Data shown are normalized to the median value for HPCs.

a

	Variance explained		
	**Expression**	**Proportions**	**Total by Factor**
Factor 1	17.8%	18.2%	36.0%
Factor 2	8.72%	15.7%	24.4%
Factor 3	6.34%	6.17%	3.84%
Factor 4	4.82%	1.51%	12.5%
Factor 5	2.18%	2.60%	4.78%
**Total**	5.00%	10.3%	81.5%

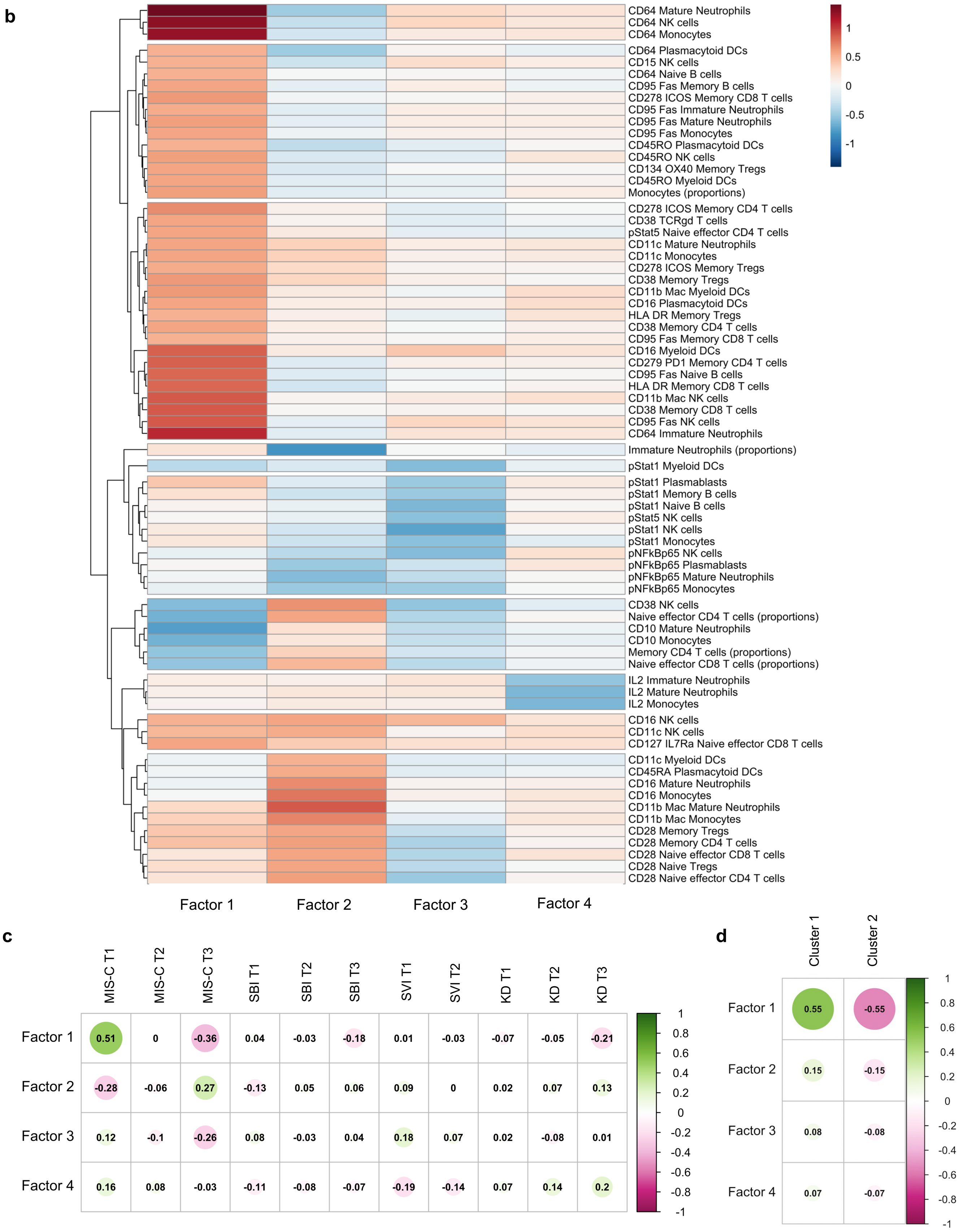

Supplementary Figure 7. Factors 1-3 by disease groups and timepoints. **a,** Factor 1 by illness group; **b,** Factor 1 for MIS-C patients over trajectory of illness; **c,** Factor 1 for SBI over trajectory of illness. **d,** Factor 2 by disease group; **e,** Factor 2 for MIS-C patients over trajectory of illness; **f,** Factor 2 for SBI over trajectory of illness. **g,** Factor 3 by disease group; **h,** Factor 3 for MIS-C patients over trajectory of illness; **i,** Factor 3 for SBI over trajectory of illness. **j,** GLM for association of inflammation severity (C-reactive protein concentration) with Factors 1–4 by illness group.

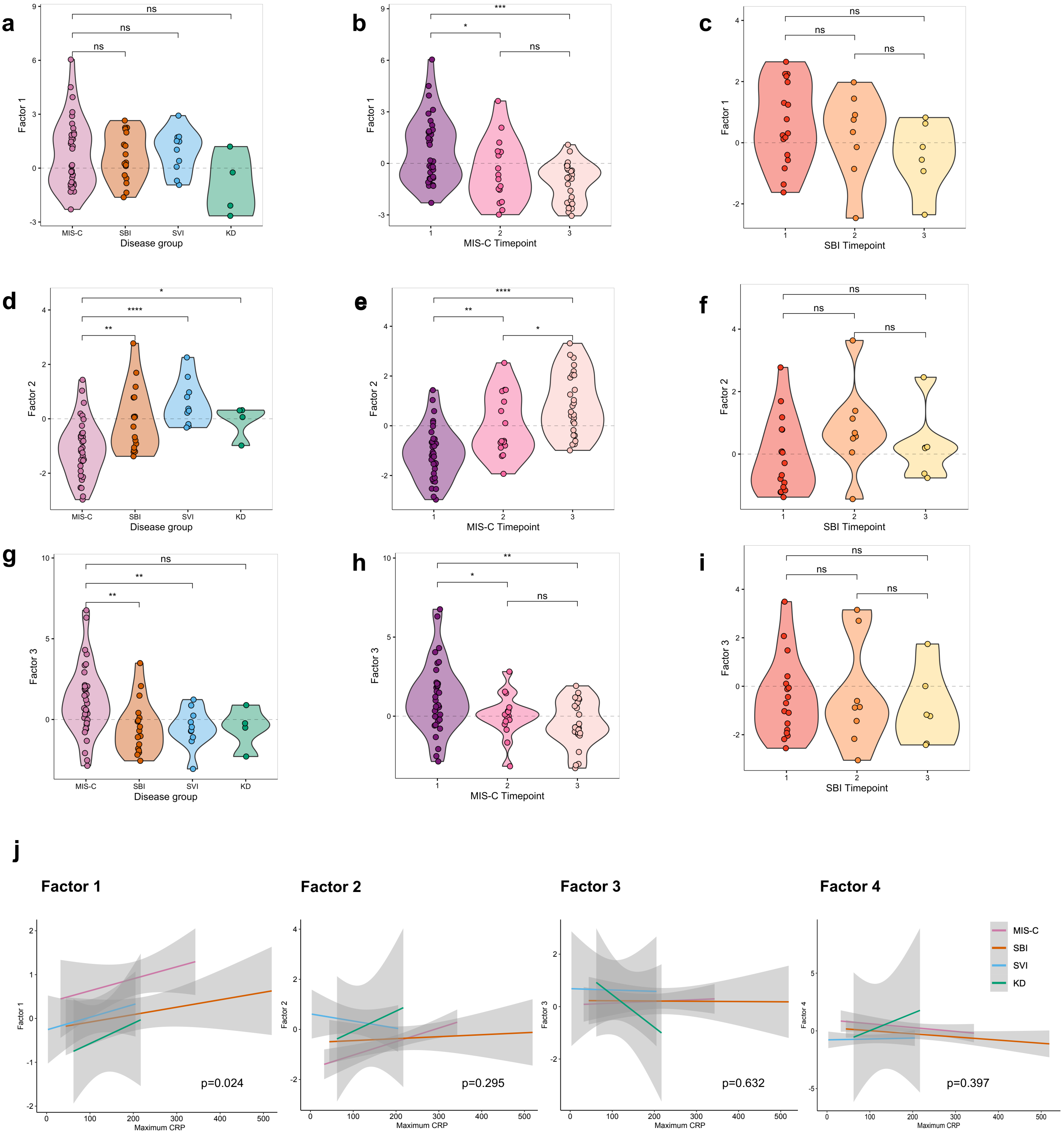

Supplementary Figure 8. Markers of immune cell activation and return to baseline in innate immune cells (mature neutrophils, immature neutrophils, monocytes, myeloid DCs, plasmacytoid DCs, CD4 memory T cells, CD8 memory T cells, memory regulatory T cells). Timepoints included are T1 (acute), T2 (defervescence) and T3 (convalescence). For all figures, patients with MIS-C are represented in shades of purple for T1–T3 and patients with SBI are represented with shades of orange for T1–T3. P values for pairwise-comparisons across time are using Wilcoxon rank sum testing.

Innate immune cells

a, Mature neutrophils. Mature neutrophil proportions, CD64 FcγRI, CD95 Fas, CD28, CD11b Mac, CD10.

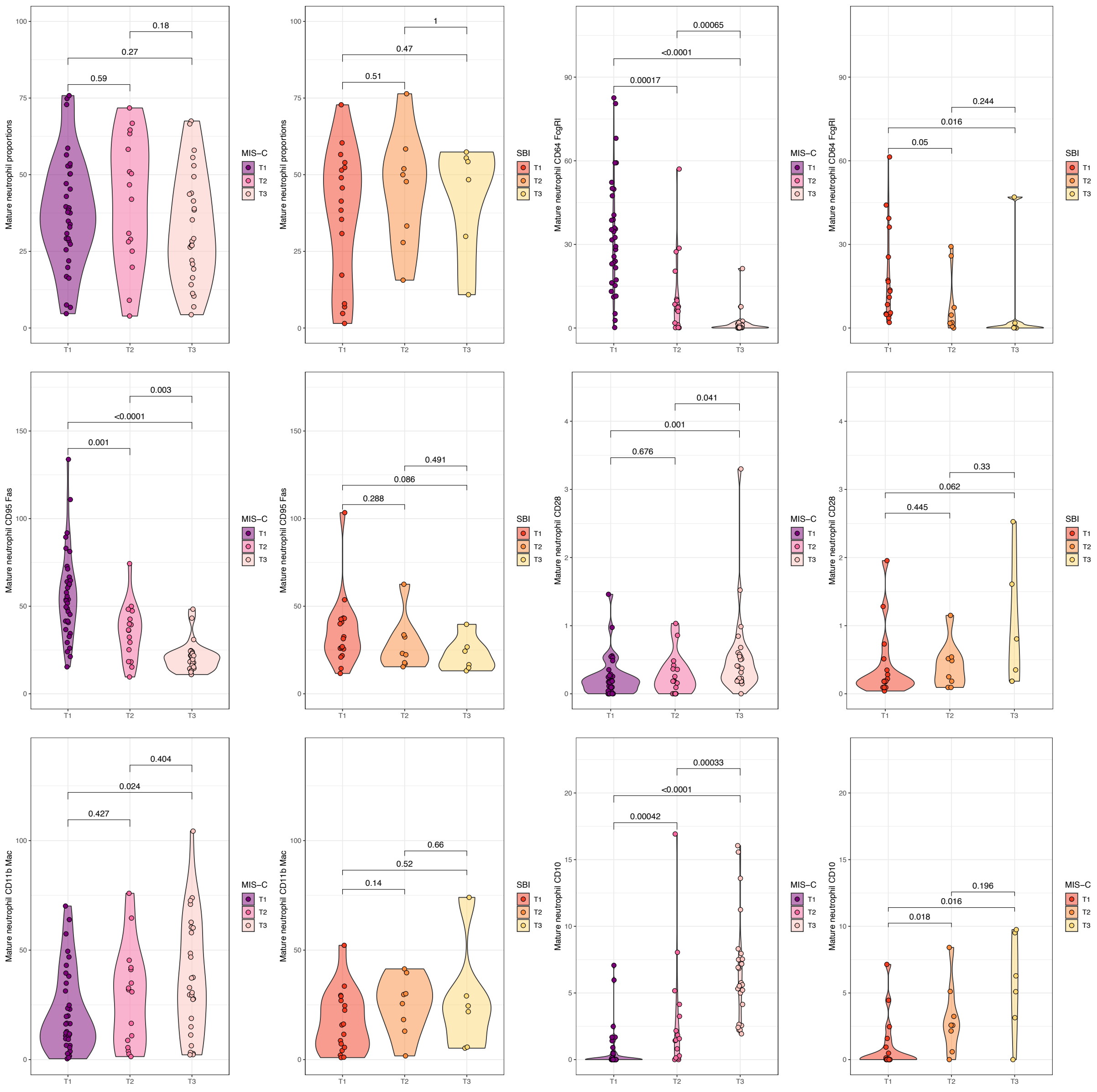

b, Immature neutrophils. Immature neutrophil proportions, CD64 FcγRI, CD95 Fas, CD28, CD11b Mac, CD10.

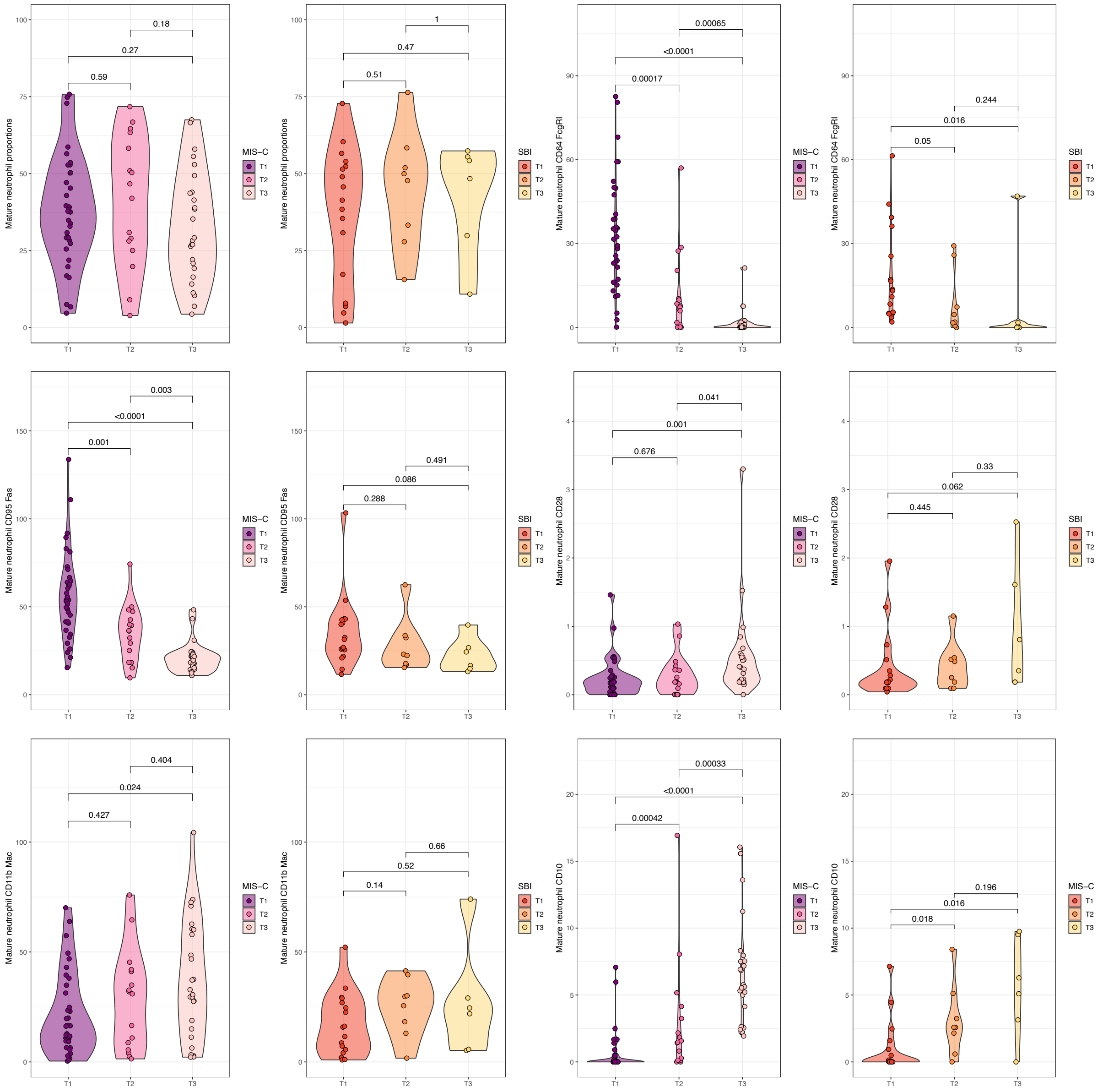

c, Monocytes. CD64 FcγRI, CD95 Fas, CD16, HLA-DR, CD28, CD10.

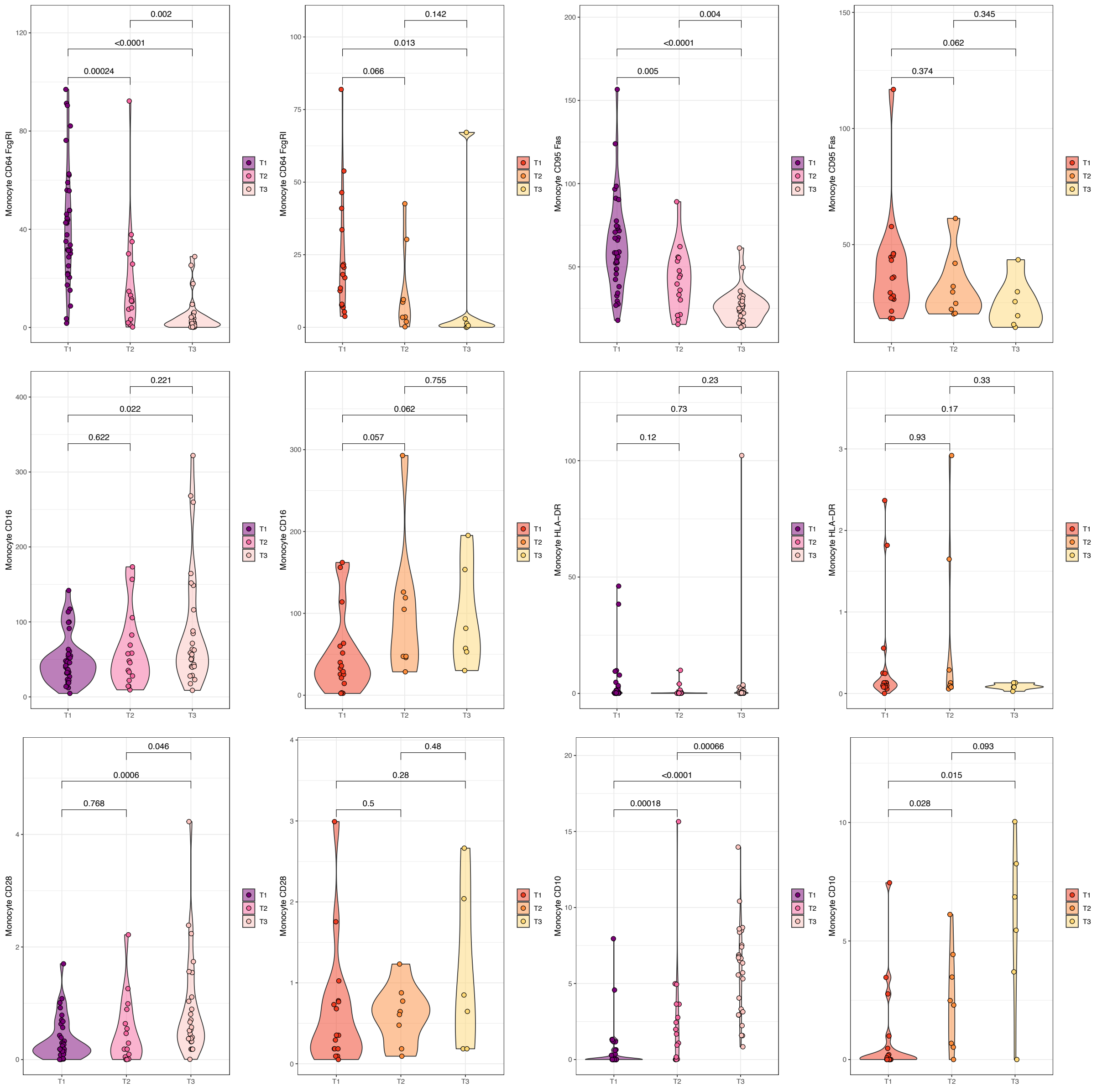

d, Myeloid DCs. CD64 FcγRI, CD95 Fas, CD16, HLA-DR, TNFα, IL6, IL2.

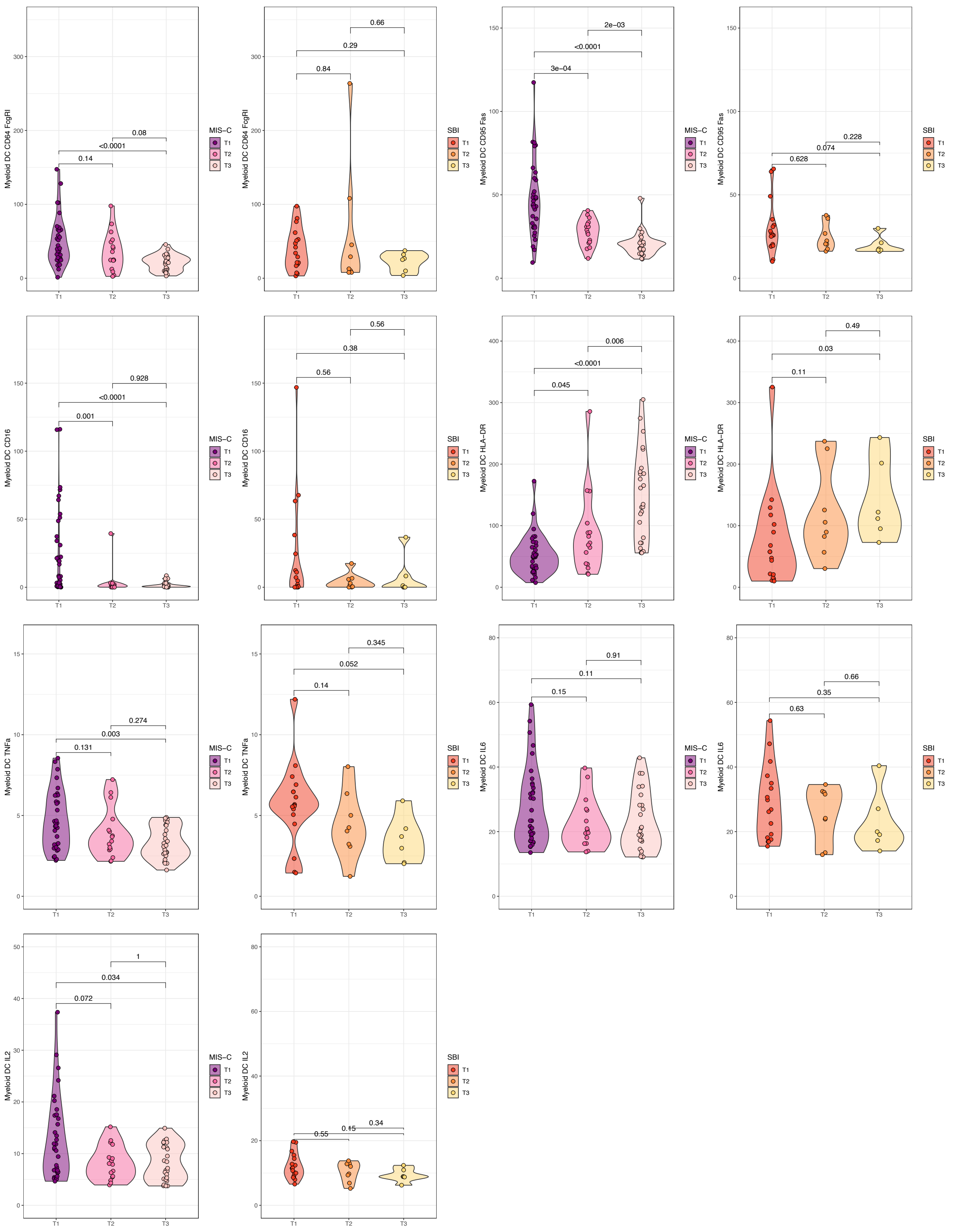

e, Plasmacytoid DCs. CD64 FcγRI, CD95 Fas, TNFα, IL6, IFNγ, pSTAT1, pSTAT5.

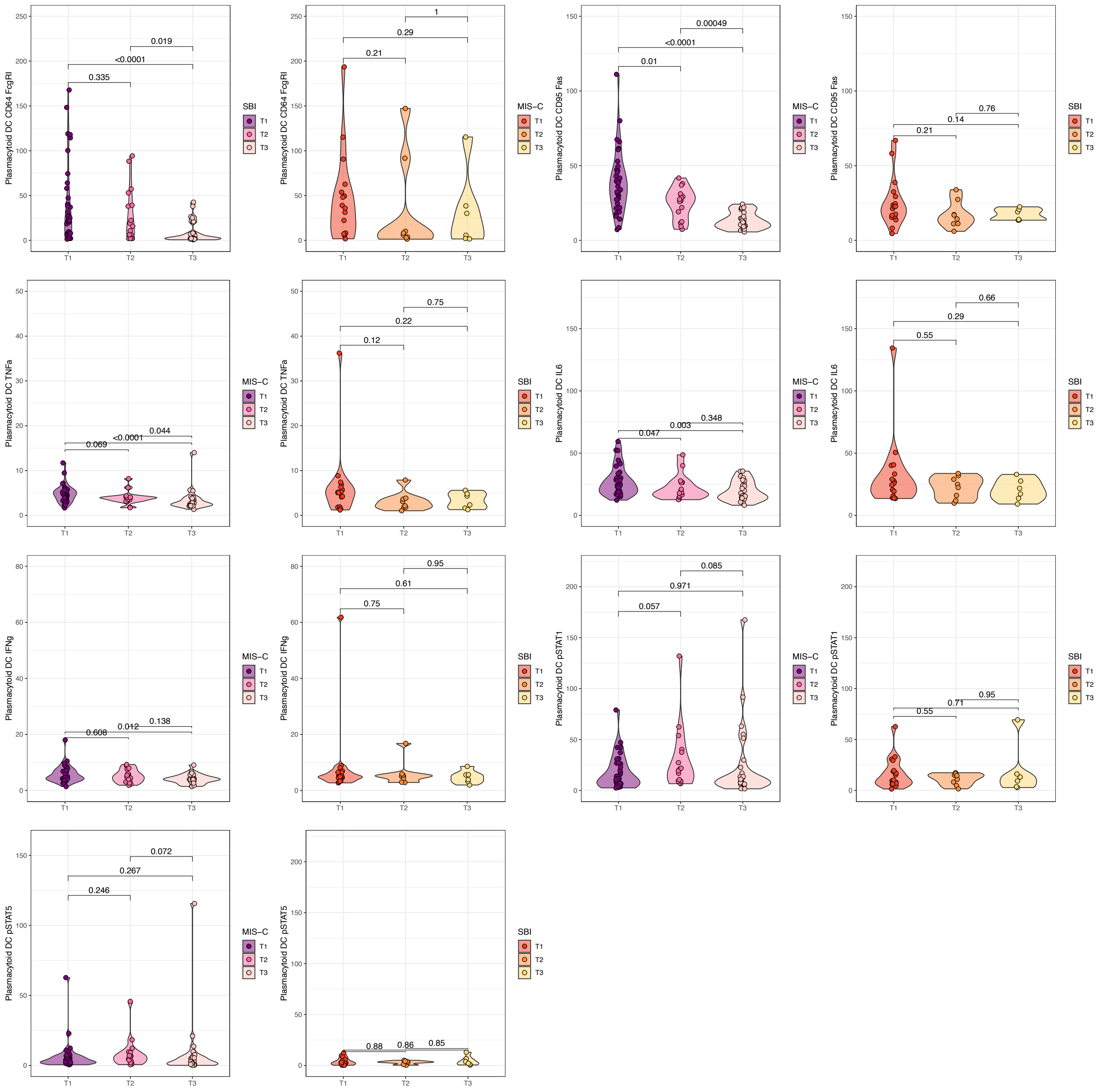

Adaptive immune cells

f, Memory CD4 T cells. HLA-DR, CD38, CD279 PD1, CD152 CTLA4, CD223 LAG3, CD278 ICOS, CD134 OX40, CD95 Fas, pSTAT1, pSTAT5, pNFκBp65, PS6, IFNγ, TNFα, IL6

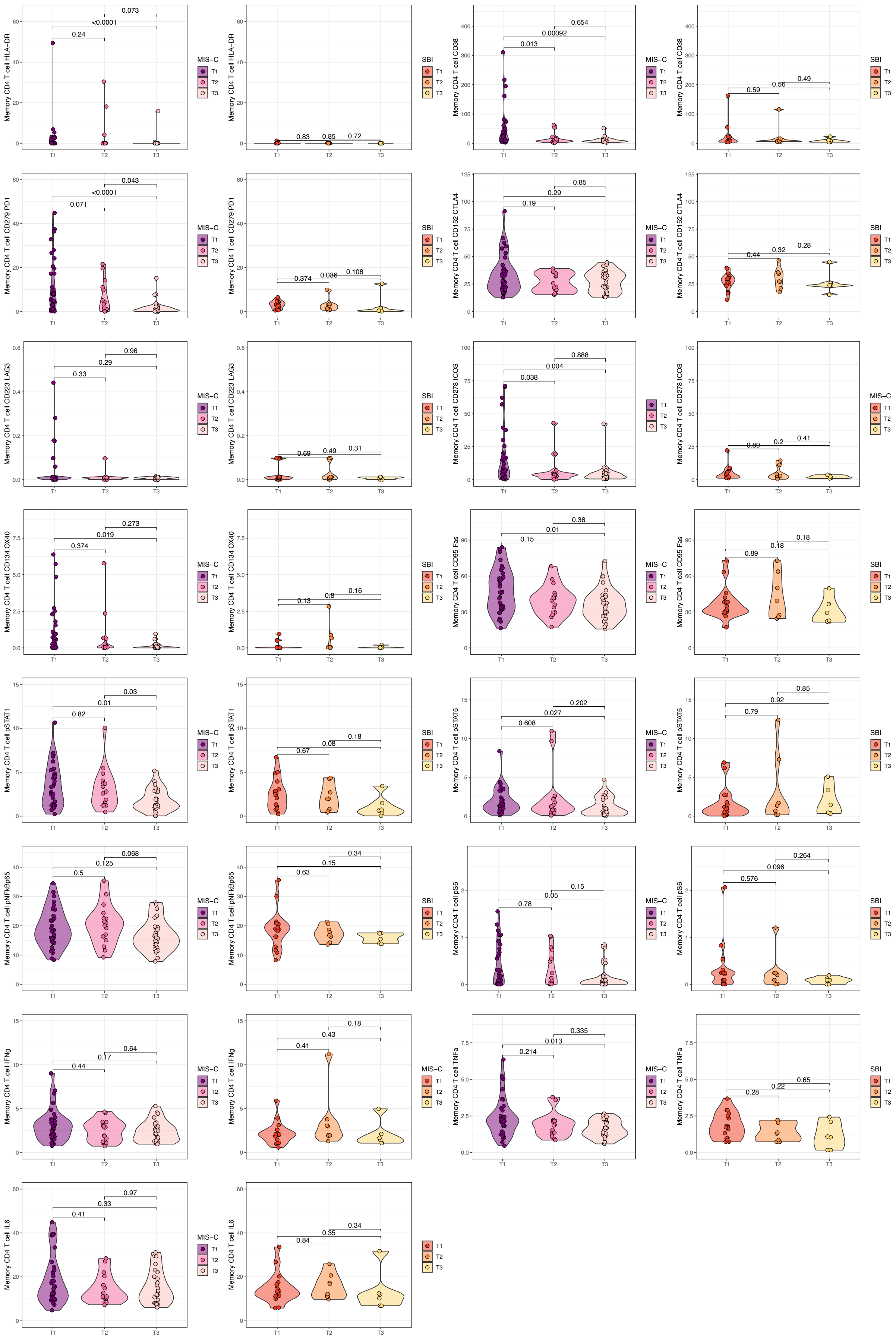
Memory CD4 T cell HLA−DR
0.073
<0.0001
0.24
MIS−C
T1
T2
T3
0.83
0.85
0.72
SBI
Memory CD4 T cell CD38
0.654
0.00092
0.013
0.49
0.56
0.59
Memory CD4 T cell CD279 PD1
0.043
<0.0001
0.071
0.374
0.036
0.108
Memory CD4 T cell CD152 CTLA4
0.85
0.29
0.19
0.44
0.32
0.28
Memory CD4 T cell CD223 LAG3
0.96
0.29
0.33
0.69
0.49
0.31
Memory CD4 T cell CD278 ICOS
0.888
0.004
0.038
0.89
0.2
0.41
Memory CD4 T cell CD134 OX40
0.273
0.019
0.374
0.13
0.8
0.16
Memory CD4 T cell CD95 Fas
0.38
0.01
0.15
0.18
0.18
0.89
Memory CD4 T cell pSTAT1
0.03
0.01
0.82
0.18
0.08
0.67
Memory CD4 T cell pSTAT5
0.202
0.027
0.608
0.85
0.92
0.79
Memory CD4 T cell pNFkBp65
0.068
0.125
0.5
0.34
0.15
0.63
Memory CD4 T cell pS6
0.15
0.05
0.78
0.264
0.096
0.576
Memory CD4 T cell IFNg
0.64
0.17
0.44
0.18
0.43
0.41
Memory CD4 T cell TNFa
0.335
0.013
0.214
0.65
0.22
0.28
Memory CD4 T cell IL6
0.97
0.33
0.41
0.34
0.35
0.84

g, Memory CD8 T cells. HLA-DR, CD38, CD279 PD1, CD152 CTLA4, CD223 LAG3, CD278 ICOS, CD134 OX40, CD95 Fas, pSTAT1, pSTAT5, pNFκBp65, PS6, IFNγ, TNFα, IL6.

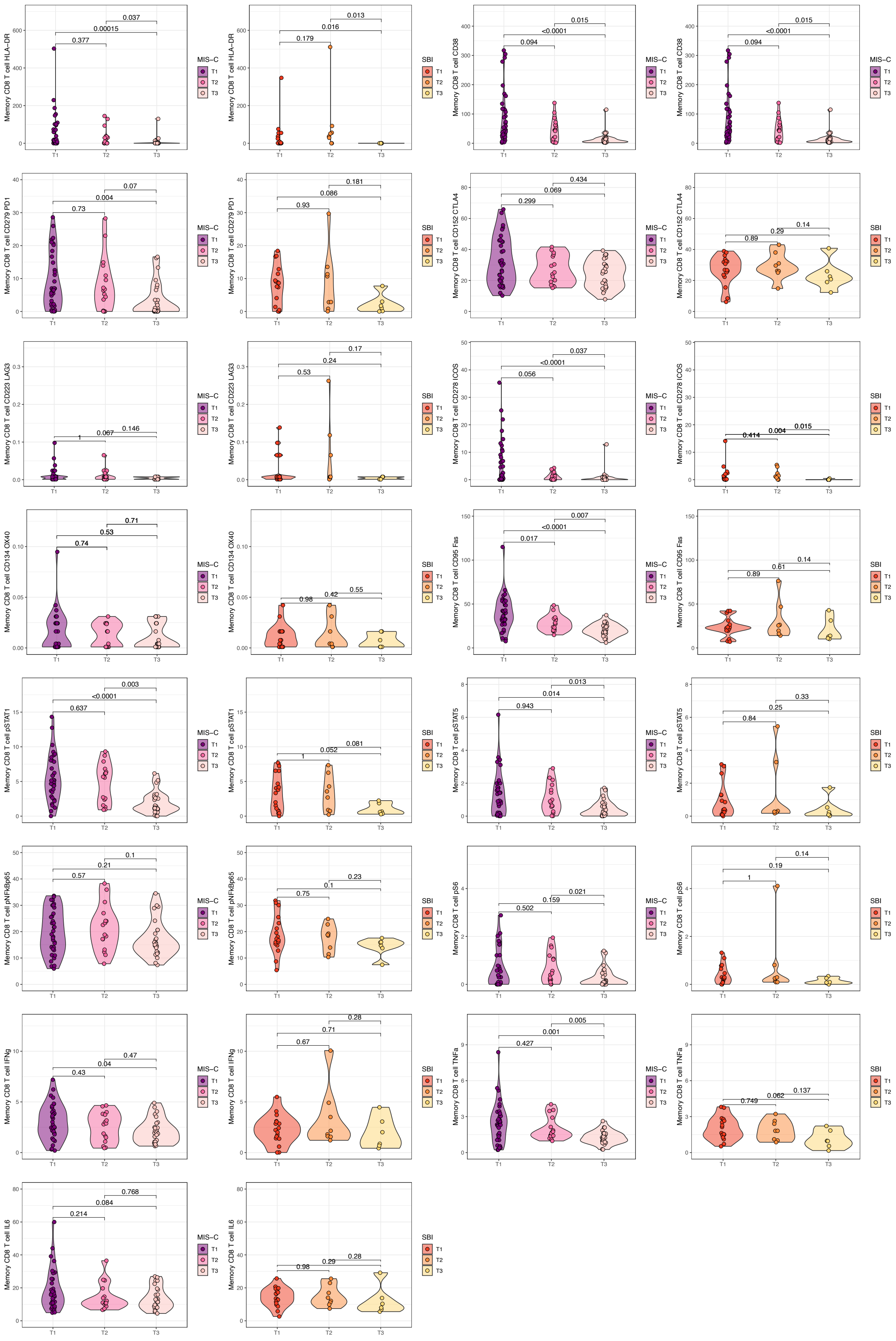

Memory CD8 T cell HLA−DR
MIS−C T1 T2 T3
0.037
0.00015
0.377
SBI T1 T2 T3
0.013
0.016
0.179
Memory CD8 T cell CD38
0.015
<0.0001
0.094
Memory CD8 T cell CD279 PD1
0.07
0.004
0.73
0.181
0.086
0.93
Memory CD8 T cell CD152 CTLA4
0.434
0.069
0.299
0.14
0.29
0.89
Memory CD8 T cell CD223 LAG3
0.146
0.067
1
0.17
0.24
0.53
Memory CD8 T cell CD278 ICOS
0.037
<0.0001
0.056
0.015
0.004
0.414
Memory CD8 T cell CD134 OX40
0.71
0.53
0.74
0.55
0.42
0.98
Memory CD8 T cell CD95 Fas
0.007
<0.0001
0.017
0.14
0.61
0.89
Memory CD8 T cell pSTAT1
0.003
<0.0001
0.637
0.081
0.052
1
Memory CD8 T cell pSTAT5
0.013
0.014
0.943
0.33
0.25
0.84
Memory CD8 T cell pNFkBp65
0.1
0.21
0.57
0.23
0.1
0.75
Memory CD8 T cell pS6
0.021
0.159
0.502
0.14
0.19
1
Memory CD8 T cell IFNg
0.47
0.04
0.43
0.28
0.71
0.67
Memory CD8 T cell TNFa
0.005
0.001
0.427
0.137
0.062
0.749
Memory CD8 T cell IL6
0.768
0.084
0.214
0.28
0.29
0.98

h, Memory regulatory T cells. HLA-DR, CD38, CD279 PD1, CD152 CTLA4, CD223 LAG3, CD278 ICOS, CD134 OX40, CD95 Fas, HLA-DR, CD45RApSTAT1, pSTAT5.

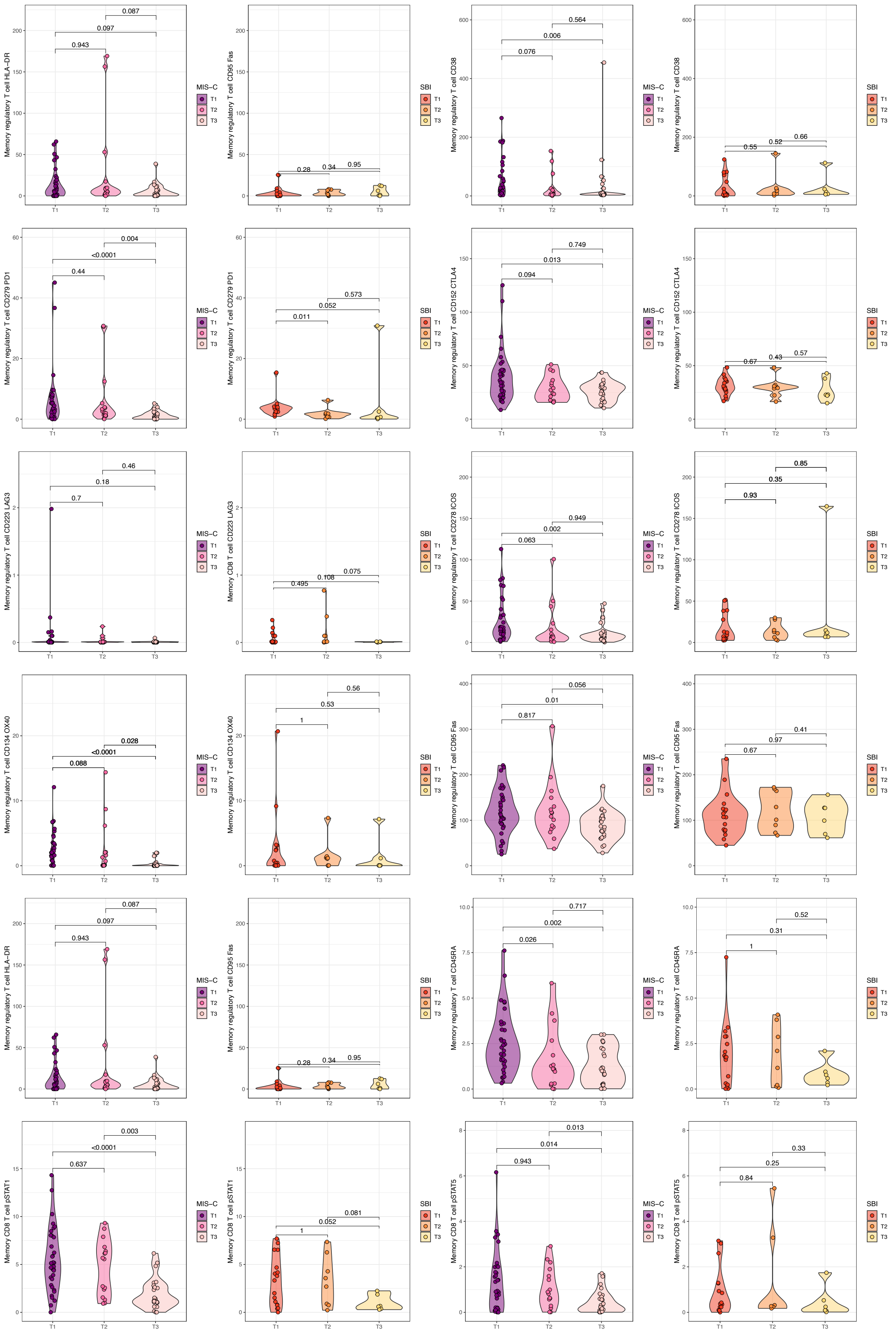
Memory regulatory T cell HLA−DR
0.087
0.097
0.943
MIS−C
T1
T2
T3
Memory regulatory T cell CD95 Fas
0.28
0.34
0.95
SBI
T1
T2
T3
Memory regulatory T cell CD38
0.564
0.006
0.076
MIS−C
Memory regulatory T cell CD38
0.55
0.52
0.66
SBI
Memory regulatory T cell CD279 PD1
0.004
<0.0001
0.44
MIS−C
Memory regulatory T cell CD279 PD1
0.573
0.052
0.011
SBI
Memory regulatory T cell CD152 CTLA4
0.749
0.013
0.094
MIS−C
Memory regulatory T cell CD152 CTLA4
0.67
0.43
0.57
SBI
Memory regulatory T cell CD223 LAG3
0.46
0.18
0.7
MIS−C
Memory CD8 T cell CD223 LAG3
0.495
0.108
0.075
SBI
Memory regulatory T cell CD278 ICOS
0.949
0.002
0.063
MIS−C
Memory regulatory T cell CD278 ICOS
0.85
0.35
0.93
SBI
Memory regulatory T cell CD134 OX40
0.028
<0.0001
0.088
MIS−C
Memory regulatory T cell CD134 OX40
0.56
0.53
1
SBI
Memory regulatory T cell CD95 Fas
0.056
0.01
0.817
MIS−C
Memory regulatory T cell CD95 Fas
0.41
0.97
0.67
SBI
Memory regulatory T cell HLA−DR
0.087
0.097
0.943
MIS−C
Memory regulatory T cell CD95 Fas
0.28
0.34
0.95
SBI
Memory regulatory T cell CD45RA
0.717
0.002
0.026
MIS−C
Memory regulatory T cell CD45RA
0.52
0.31
1
SBI
Memory CD8 T cell pSTAT1
0.003
<0.0001
0.637
MIS−C
Memory CD8 T cell pSTAT1
0.081
0.052
1
SBI
Memory CD8 T cell pSTAT5
0.013
0.014
0.943
MIS−C
Memory CD8 T cell pSTAT5
0.33
0.25
0.84
SBI

Supplementary Figure 9. a, Markers of T cell activation, exhaustion and apoptosis in memory CD4 T cells. Co-expression of HLA-DR, CD38, CD279 (PD-1) with CD152 (CTLA4), CD223 (LAG3), CD278 (ICOS), CD134 (OX40), CD95 (Fas), and intracellular IFNγ expression in memory CD4 T cells in all children with at T1, T2 and T3 (colored by illness group). P-values and adjusted R-squared values are for all observations (regardless of illness group).

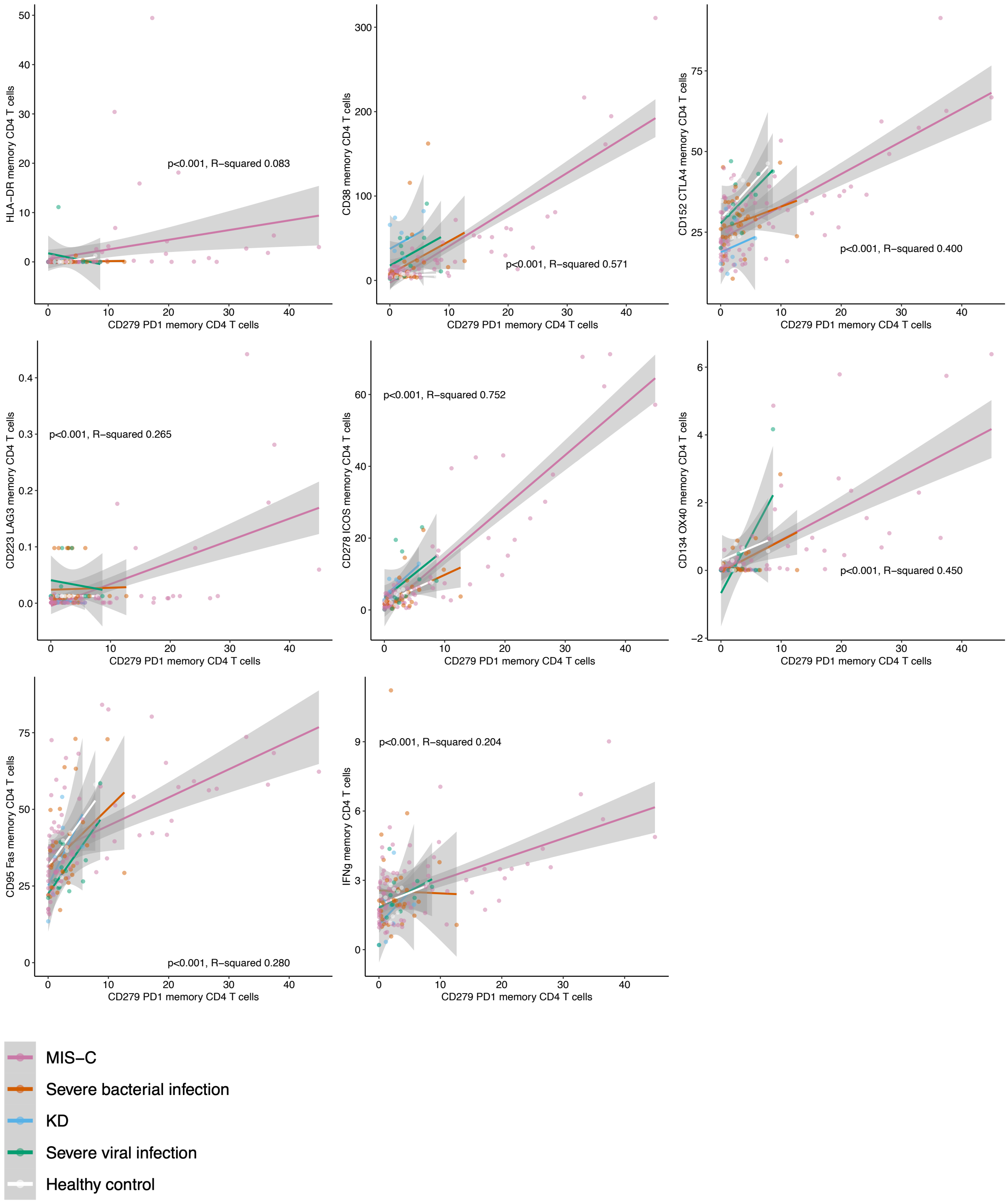

b, Markers of T cell activation, exhaustion and apoptosis in memory CD8 T cells. Co-expression of CD279 (PD-1) with CD152 (CTLA4), CD223 (LAG3), CD278 (ICOS), CD134 (OX40), CD95 (Fas), and the intracellular IFNγ in memory CD8 T cells in all children with at T1, T2 and T3 (colored by illness group). P-values and adjusted R-squared values are for all observations (regardless of illness group).

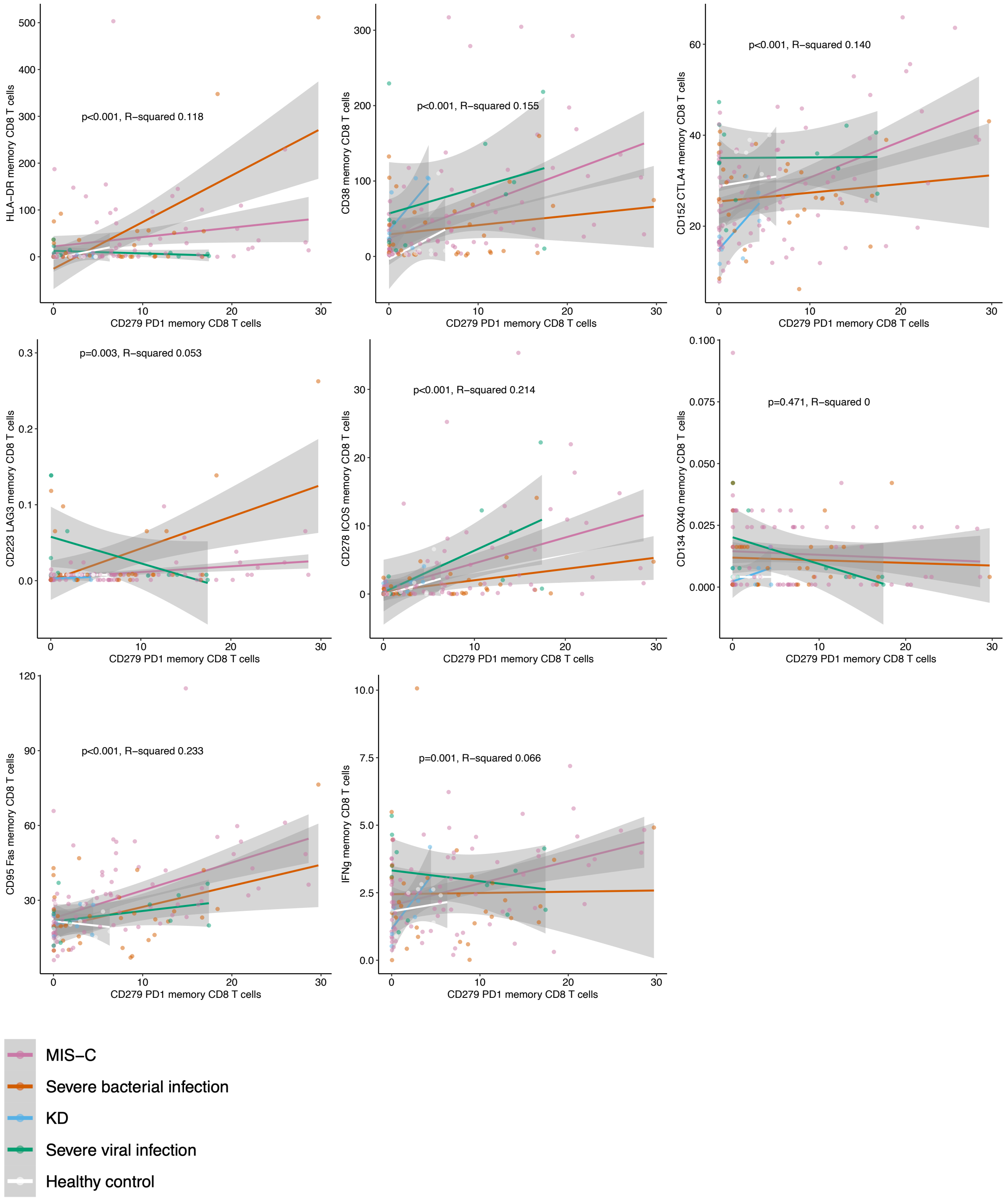

c, Markers of T cell activation, exhaustion and apoptosis in memory regulatory T cells. Co-expression of CD279 (PD-1) with CD152 (CTLA4), CD223 (LAG3), CD278 (ICOS), CD134 (OX40), CD95 (Fas) memory regulatory T cells in all children with at T1, T2 and T3 (colored by illness group). P-values and adjusted R-squared values are for all observations (regardless of illness group).

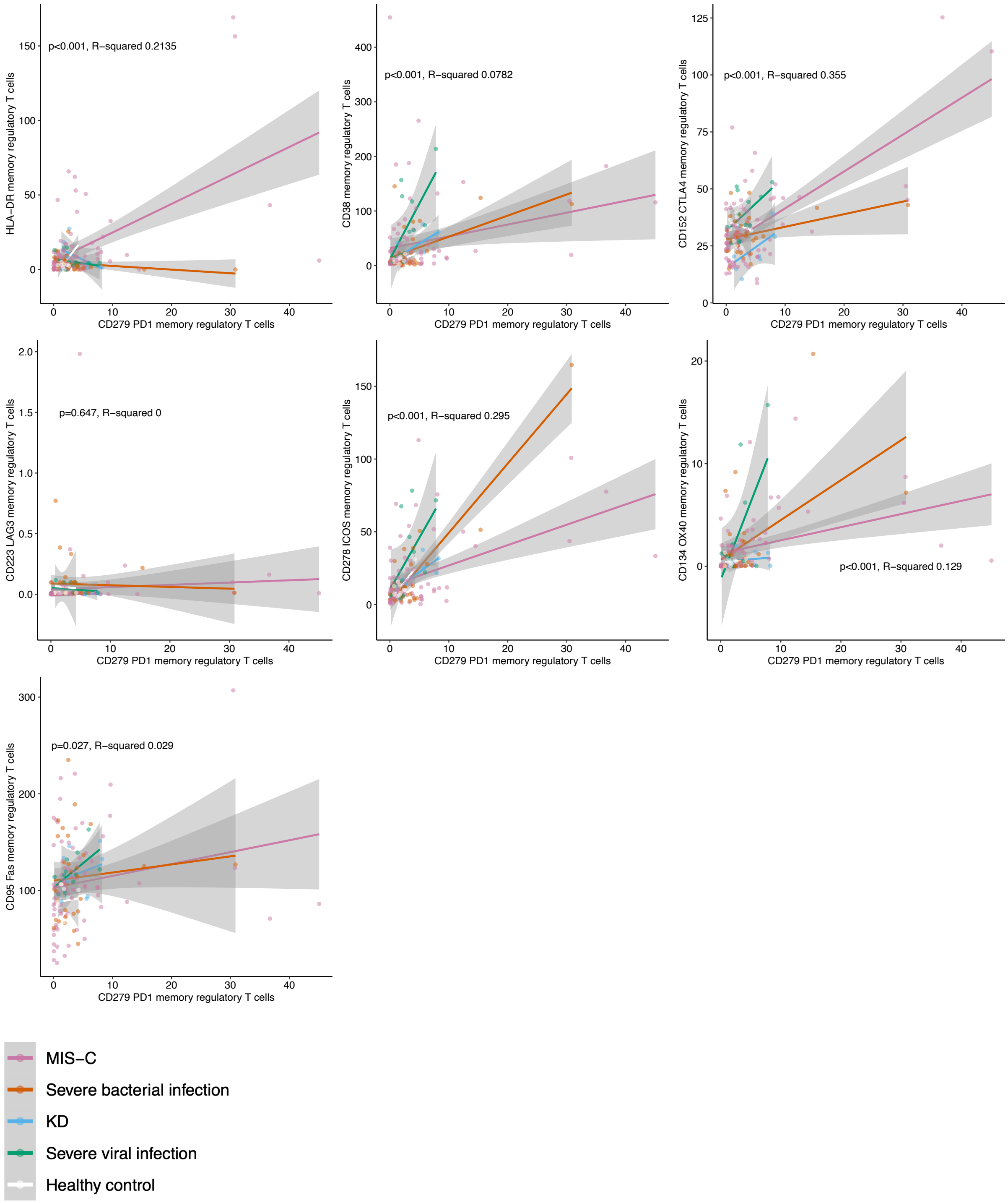

Supplementary Figure 10. Co-expression of CD279 PD1 with markers of activation, exhaustion and apoptosis in CD4 memory T cells. Representative examples of CD279 PD-1 expression (x axis, all plots) versus **a,** HLA-DR; **b,** CD38; **c,** CD152 CTLA4; **d,** CD134 OX40, **e,** CD278 ICOS, **f,** CD95 Fas, **g,** intracellular IFNγ in memory CD4 T cells and naïve/effector CD4 T cells. Plots show data from T1 from a sample from a child with MIS-C with high expression of CD279 PD1; and a healthy paediatric control sample.

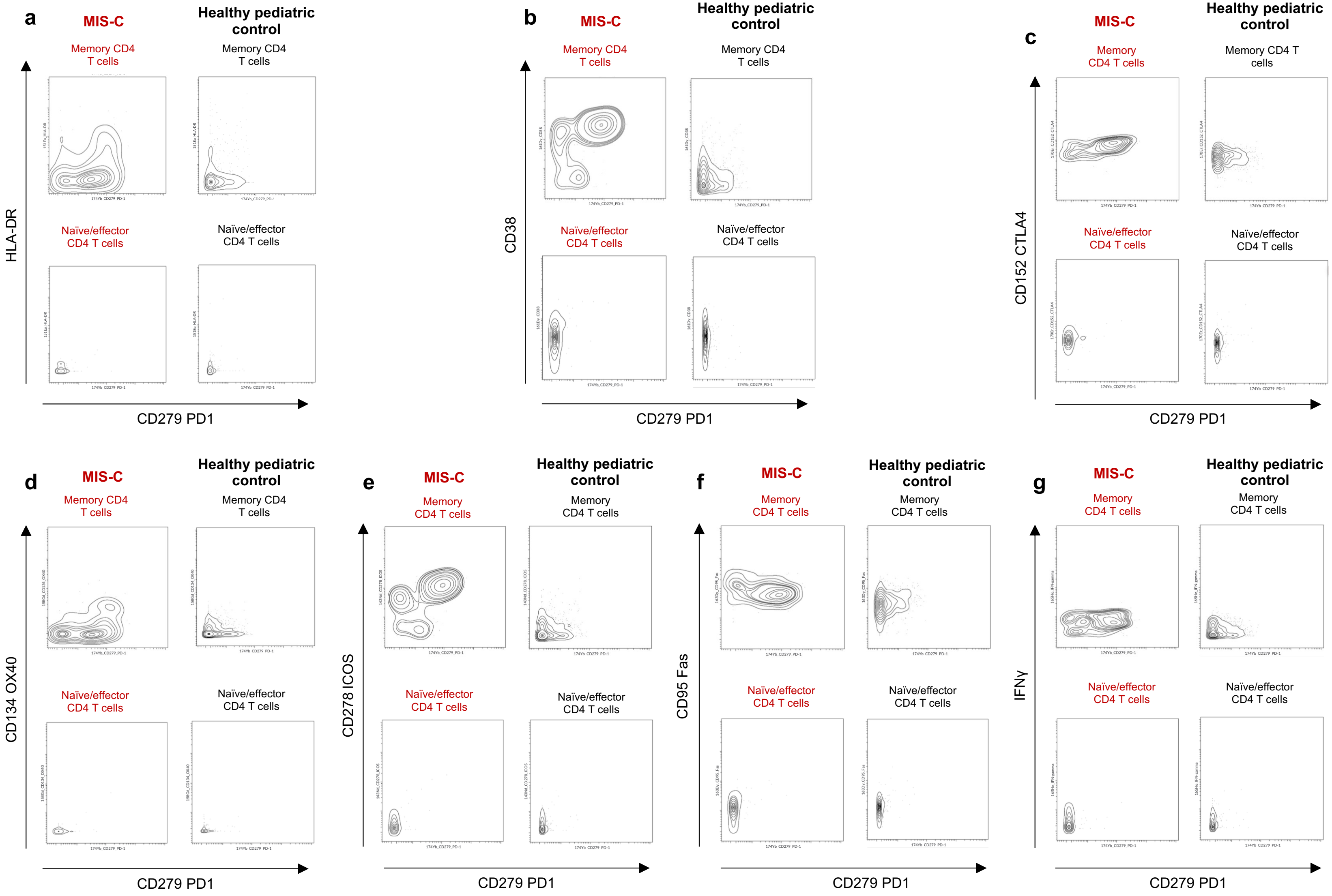

a
MIS-C
Healthy pediatric control
Memory CD4 T cells
Memory CD4 T cells
Naïve/effector CD4 T cells
Naïve/effector CD4 T cells
HLA-DR
CD279 PD1
b
MIS-C
Healthy pediatric control
Memory CD4 T cells
Memory CD4 T cells
Naïve/effector CD4 T cells
Naïve/effector CD4 T cells
CD38
CD279 PD1
c
MIS-C
Healthy pediatric control
Memory CD4 T cells
Memory CD4 T cells
Naïve/effector CD4 T cells
Naïve/effector CD4 T cells
CD152 CTLA4
CD279 PD1
d
MIS-C
Healthy pediatric control
Memory CD4 T cells
Memory CD4 T cells
Naïve/effector CD4 T cells
Naïve/effector CD4 T cells
CD134 OX40
CD279 PD1
e
MIS-C
Healthy pediatric control
Memory CD4 T cells
Memory CD4 T cells
Naïve/effector CD4 T cells
Naïve/effector CD4 T cells
CD278 ICOS
CD279 PD1
f
MIS-C
Healthy pediatric control
Memory CD4 T cells
Memory CD4 T cells
Naïve/effector CD4 T cells
Naïve/effector CD4 T cells
CD95 Fas
CD279 PD1
g
MIS-C
Healthy pediatric control
Memory CD4 T cells
Memory CD4 T cells
Naïve/effector CD4 T cells
Naïve/effector CD4 T cells
IFNγ
CD279 PD1

Supplementary Figure 11. K-means clustering analysis: effects of immunomodulatory treatment on supernatant cytokines. K-means clustering plots for supernatant cytokines samples with no stimulation (**a**), stimulation with SARS-CoV-2 antigen (ag1) (**b**) and stimulation with mitogen (phytohemagglutinin) (**c**). The clusters and their respective centre points are represented by different colours. The shape of each point represents the treatment received prior to research blood sampling.

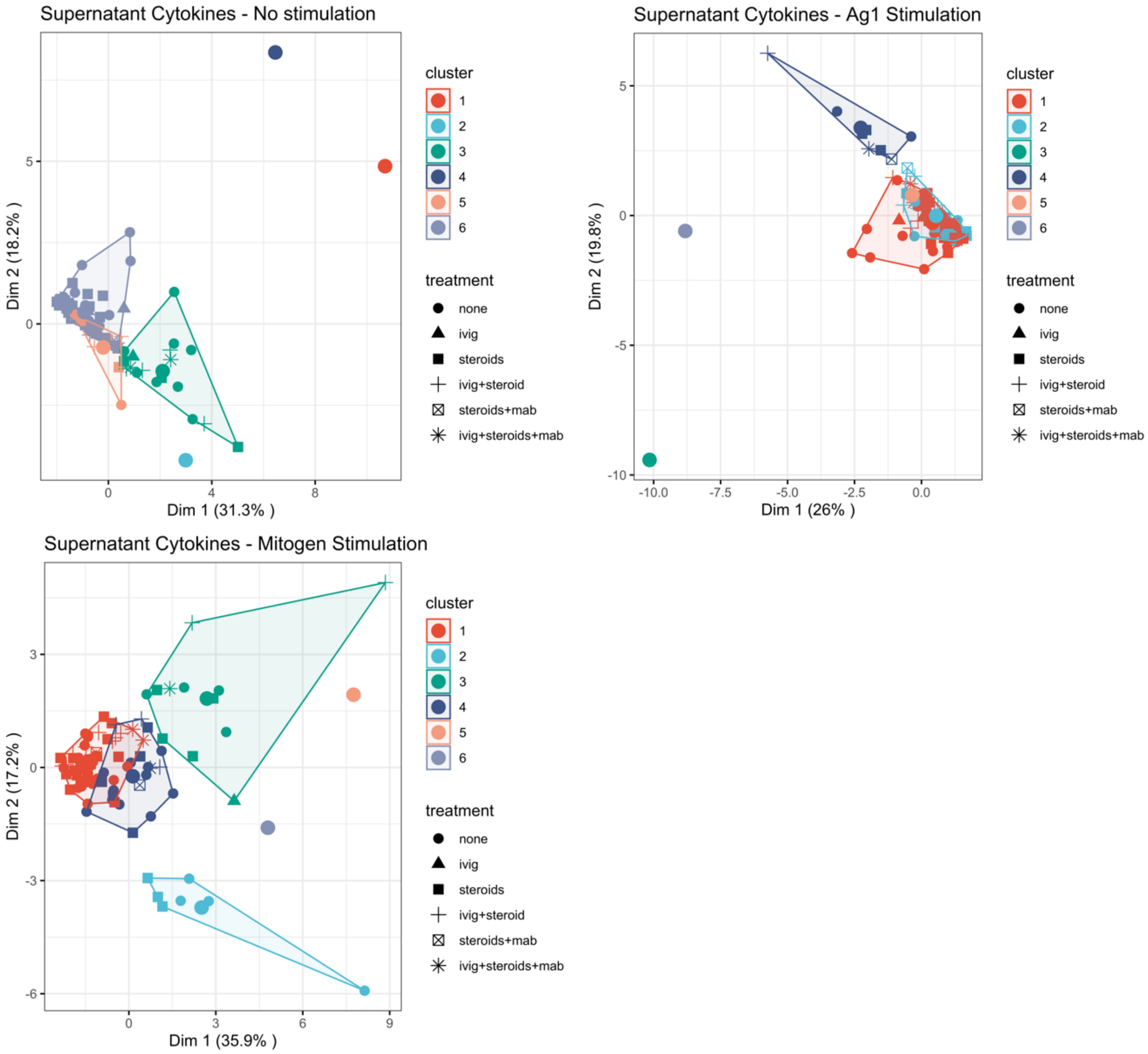

Supplementary Figure 12. Supernatant cytokine analysis 1**:** interferons

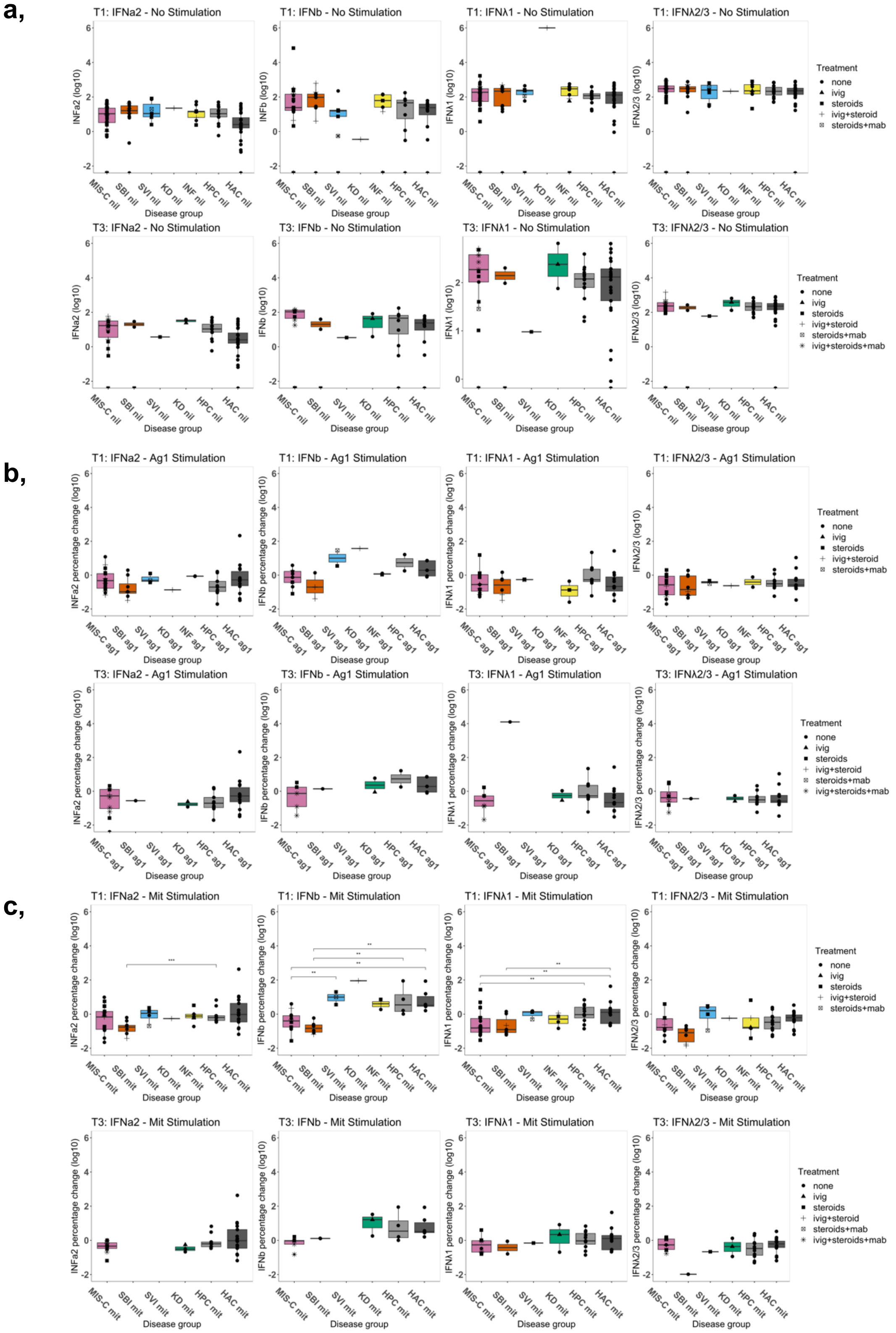

Box plots showing concentration of cell stimulation assay supernatant cytokines levels: interferon (IFN)α2, IFNβ, IFNλ1, IFNλ2/3; Log_{10} values are shown. Each point representations a participant for the following groups: Multisystem inflammatory syndrome in children (MIS-C), severe bacterial illness (SBI), severe viral illness (SVI), Kawasaki disease (KD), other inflammatory disease. Absolute cytokine levels from acute (T1) and convalescent (T3) samples at baseline – unstimulated (**a**) percentage increase from baseline following stimulation with mitogen (mit) (**b**), and absolute

increase from baseline following stimulation with SARS-CoV-2 antigen (ag1) (**c**) are shown. The shape of each point indicates the treatment received prior to sampling.

Supplementary Figure 13. Supernatant cytokine analysis 2: remaining cytokines

a, b, c,

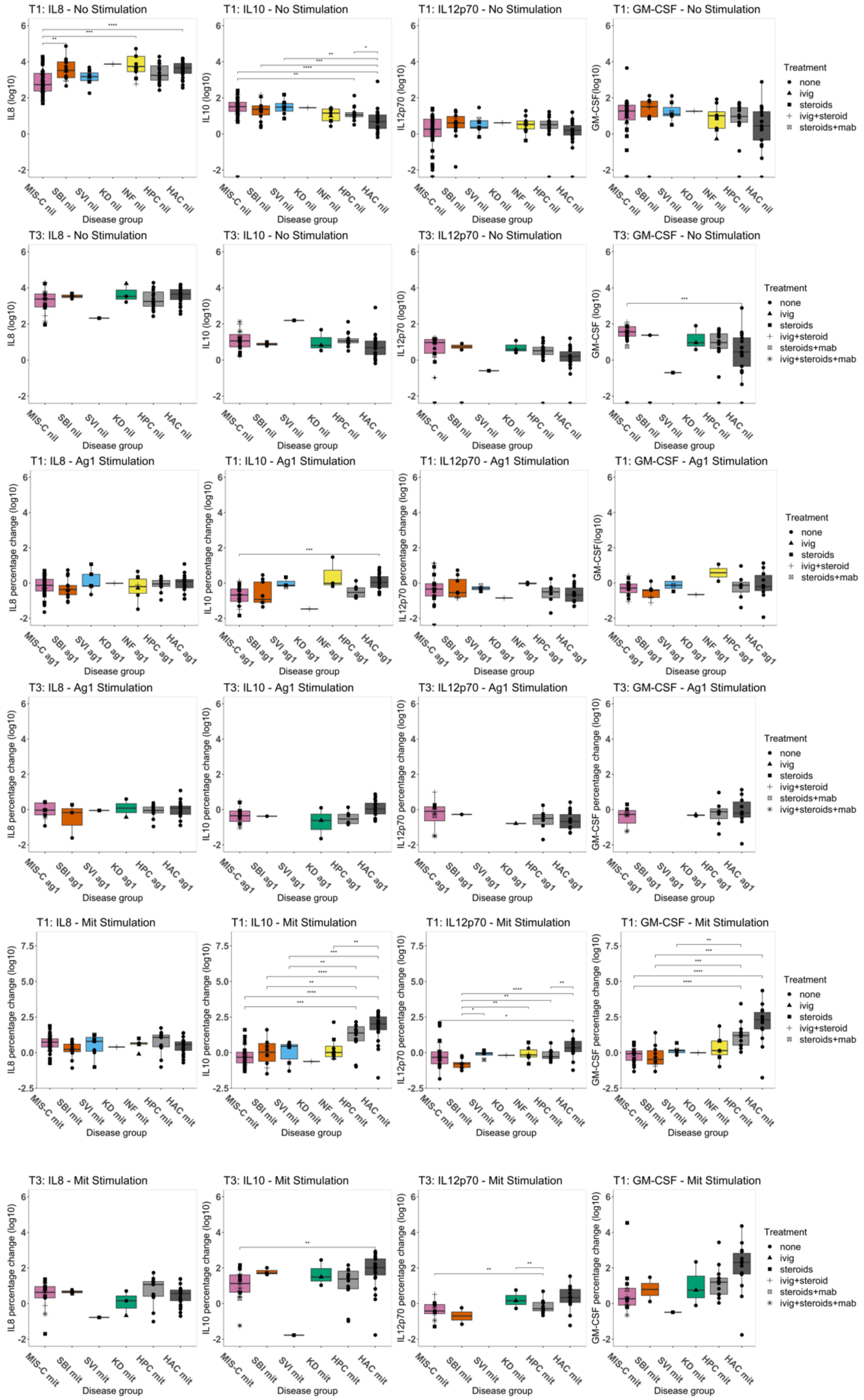

Box plots showing concentration of cell stimulation assay supernatant cytokines levels: interleukin (IL)8, IL10, IL12p70, granulocyte-macrophage colony-stimulating factor (GM-CSF*).* Log_{10} values are shown. Each point representations a participant for the following groups: Multisystem inflammatory syndrome in children (MIS-C), severe

bacterial illness (SBI), severe viral illness (SVI), Kawasaki disease (KD), other inflammatory disease. Absolute cytokine levels from acute (T1) and convalescent (T3) samples at baseline – unstimulated (**a**) percentage increase from baseline following stimulation with mitogen (mit) (**b**), and absolute increase from baseline following stimulation with SARS-CoV-2 antigen (ag1) (**c**) are shown. The shape of each point indicates the treatment received prior to sampling.

Supplementary Figure 14. Supernatant cytokine sub-analysis of MIS-C T1 by clusters identified on mass cytometry.

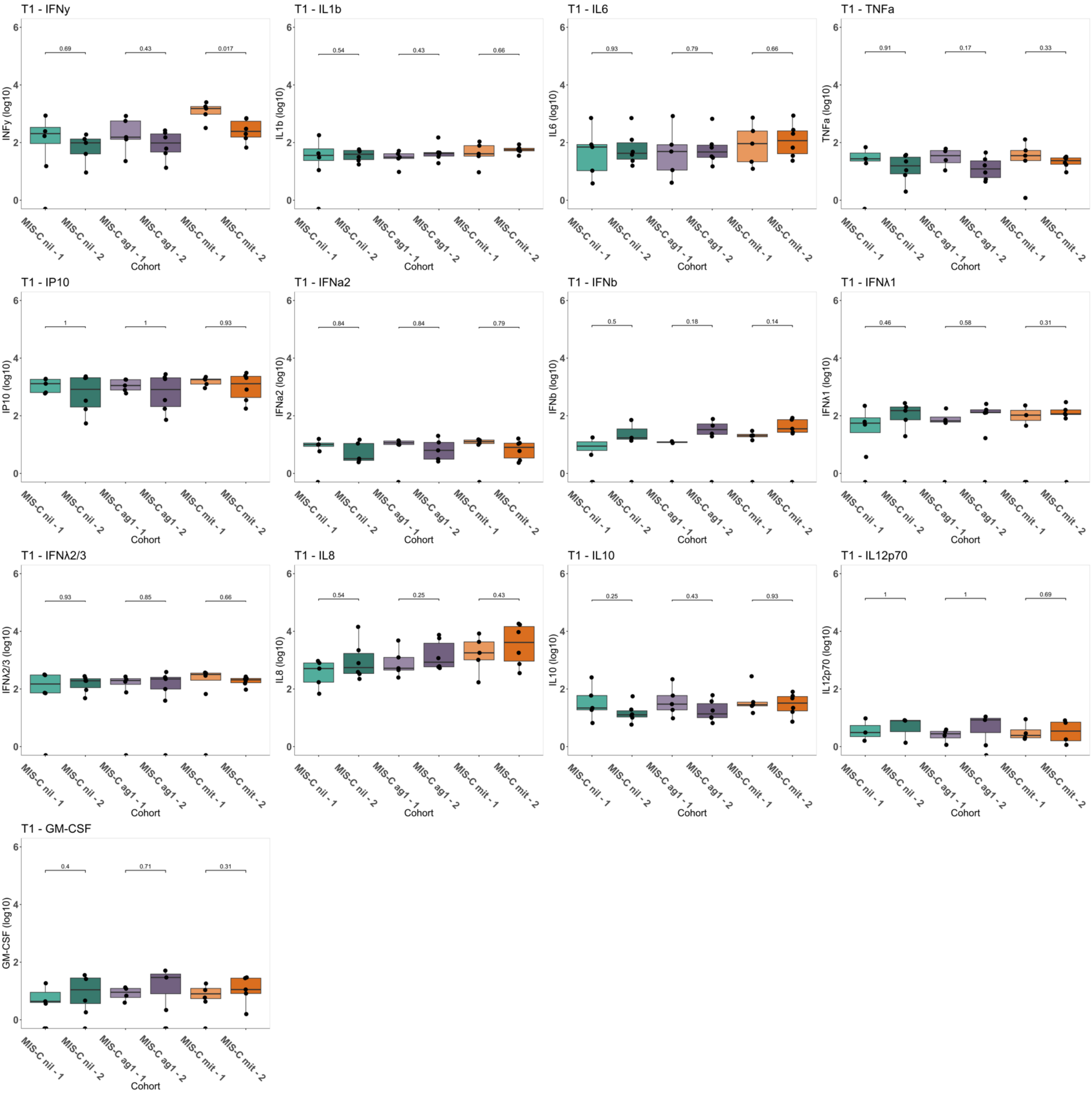

Box plots showing concentration of cell stimulation assay supernatant cytokines levels in acute (T1) MIS-C by clusters 1 and 2 identified on mass cytometry: IFNγ, IL1β, IL6, TNFα, IP10, IFNα2, IFNβ, IFNγ, IFNλ1, IFNλ2/3, IL8, IL10, IL12p70 and GM-CSF.

Log_{10} transformed raw data at baseline and following stimulation with SARS-CoV-2 antigen (ag1) and mitogen (mit) are shown. Each point represents a patient sample. P values from Wilcoxon pairwise comparisons are displayed.

Supplementary Figure 15. Heatmap summarising mass cytometry results of stimulated samples: foldchange from baseline following stimulation with SARS-CoV-2 antigen and mitogen for variables related to the a) innate immune response and b) T cell immune response.

a

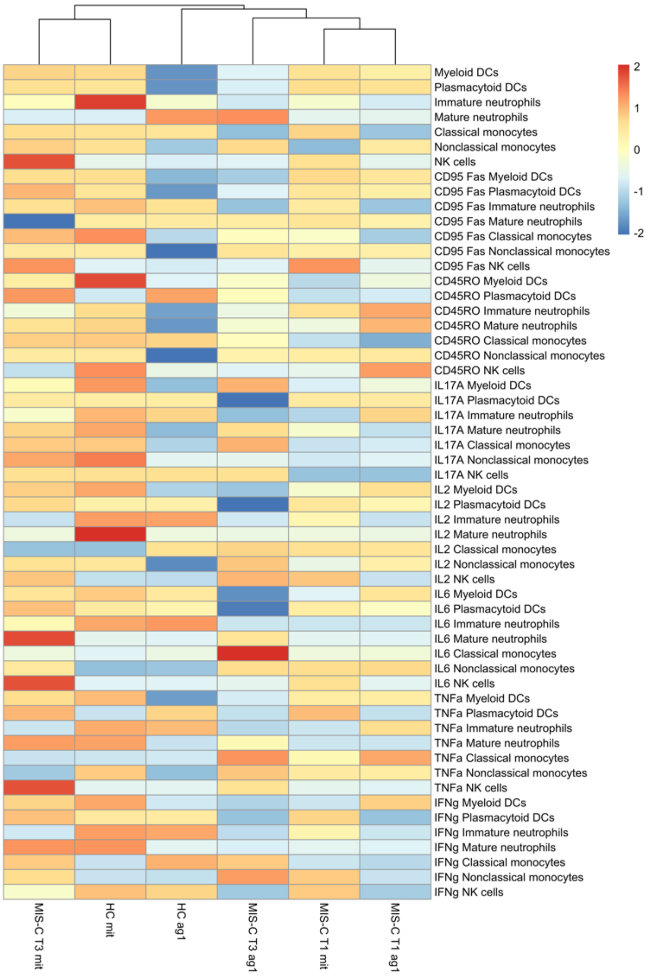

b

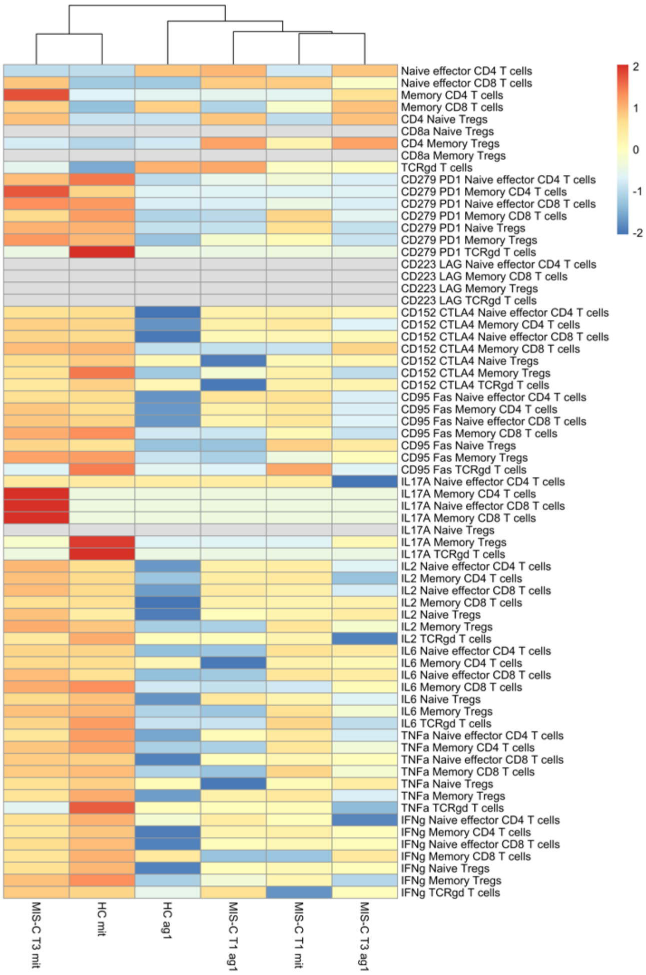

Supplementary Figure 16. Gene set enrichment analysis (GSEA) results. **a,** children with MIS-C (TP1) versus pediatric healthy controls; **b,** children with definite bacterial infection (TP1) versus pediatric healthy controls; **c,** children with definite viral infection (TP1) versus pediatric healthy controls; **d,** children with MIS-C versus children with definite bacterial infection. GSEA was undertaken in the R statistical environment, using pathways annotated by the Gene Ontology (GO) database.

a

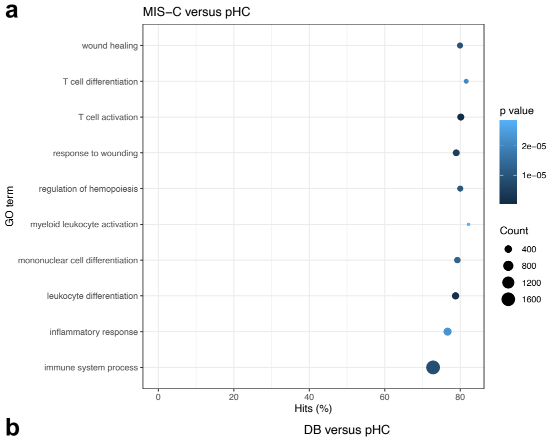

MIS-C versus pediatric healthy controls

Term	DE genes	Genes in category	Percent DE genes (hits)
Myeloid leukocyte activation	157	191	82.2
T cell differentiation	186	228	81.6
T cell activation	339	423	80.1
Regulation of haemopoiesis	256	320	80.0
Wound healing	255	319	79.9
Mononuclear cell differentiation	290	366	79.2
Response to wounding	326	413	78.9
Leukocyte differentiation	367	466	78.8
Inflammatory response	456	595	76.6
Immune system process	1649	2265	72.8

b

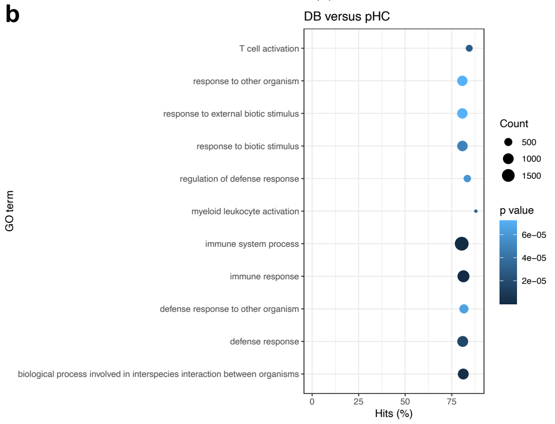

Definite bacterial infection (DB) versus pediatric healthy controls

Term	DE genes	Genes in category	Percent DE genes (hits)
Myeloid leukocyte activation	168	191	88.0
T cell activation	357	423	84.4
Regulation of defense response	416	499	83.4
Defense response to other organism	680	834	81.5
Immune response	1290	1587	81.3
Biological process involved in interspecies interaction between organisms	1023	1260	81.2
Defense response	1024	1266	80.9
Response to biotic stimulus	939	1163	80.7
Response to external biotic stimulus	911	1129	80.7
Immune system process	1820	2265	80.4

c

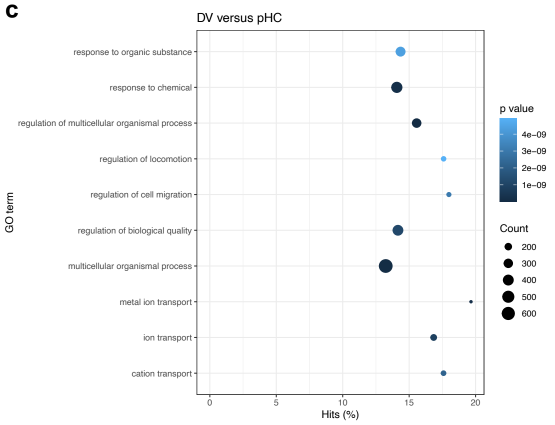

Definite viral infection (DV) versus pediatric healthy controls

Term	DE genes	Genes in category	Percent DE genes (hits)
Innate immune response	451	682	66.1
Response to cytokine	472	743	63.5
Defense response to other organism	529	834	63.4
Defense response	785	1266	62.0
Biological process involved in interspecies interaction between organisms	779	1260	61.8
Response to external biotic stimulus	697	1129	61.7
Response to other organism	697	1129	61.7
Response to external stimulus	1238	2049	60.4
Response to organic substance	1370	2306	59.4
Response to stress	1793	3045	58.9

d

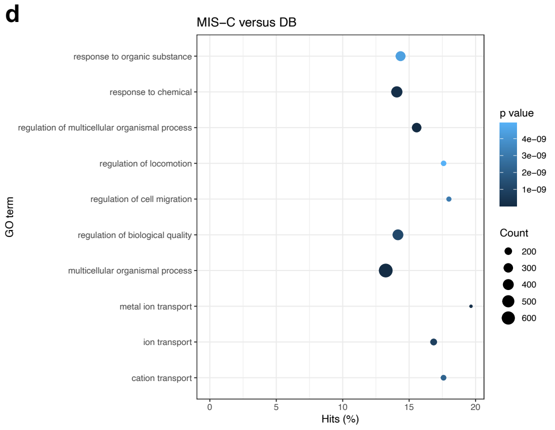

MIS-C versus definite bacterial infection (DB)

Term	DE genes	Genes in category	Percent DE genes (hits)
Metal ion transport	114	580	19.7
Regular of cell migration	129	717	18.0
Cation transport	140	796	17.6
Regulation of locomotion	136	773	17.6
Ion transport	173	1027	16.8
Regulation of multicellular organismal process	306	1966	15.6
Response to organic substance	331	2306	14.4
Regulation of biological quality	393	2776	14.2
Response to chemical	423	3006	14.1
Multicellular organismal process	660	4986	13.2

DIAMONDS Consortium Membership

Supplemental Online Content: Nonauthor Collaborators
*First name, last name, and suffix (if applicable) are required and will appear in PubMed.

*Group Name(s): DIAMONDS consortium							
***First Name and Middle Initial(s)**	***Last Name**	***Suffix (eg, Jr, III)**	Academic Degrees	Institution	Location (city, state/province, country)	Role or Contribution, eg, chair, principal investigator	Group (if more than 1 Group listed in the byline) and/or Subgroup (eg, Steering Committee)
Aubrey	Cunnington			Imperial College London		Consortium member	
Jethro	Herberg			Imperial College London		Consortium member	
Myrsini	Kaforou			Imperial College London		Consortium member	
Victoria J.	Wright			Imperial College London		Consortium member	
Evangelos	Bellos			Imperial College London		Consortium member	
Claire	Broderick			Imperial College London		Consortium member	
Samuel	Channon-Wells			Imperial College London		Consortium member	
Samantha	Cooray			Imperial College London		Consortium member	
Tisham	De			Imperial College London		Consortium member	
Giselle	D'Souza			Imperial College London		Consortium member	
Leire	Estramiana Elorrieta			Imperial College London		Consortium member	

Diego	Estrada-Rivadeneyra			Imperial College London		Consortium member	
Rachel	Galassini			Imperial College London		Consortium member	
Dominic	Habgood-Coote			Imperial College London		Consortium member	
Shea	Hamilton			Imperial College London		Consortium member	
Heather	Jackson			Imperial College London		Consortium member	
James	Kavanagh			Imperial College London		Consortium member	
Ilana	Keren			Imperial College London		Consortium member	
Mahdi	Moradi Marjaneh			Imperial College London		Consortium member	
Stephanie	Menikou			Imperial College London		Consortium member	
Samuel	Nichols			Imperial College London		Consortium member	
Ruud	Nijman			Imperial College London		Consortium member	
Ivana	Pennisi			Imperial College London		Consortium member	
Oliver	Powell			Imperial College London		Consortium member	
Ruth	Reid			Imperial College London		Consortium member	
Priyen	Shah			Imperial College London		Consortium member	
Ortensia	Vito			Imperial College London		Consortium member	

Elizabeth	Whittaker			Imperial College London		Consortium member	
Clare	Wilson			Imperial College London		Consortium member	
Rebecca	Womersley			Imperial College London		Consortium member	
Amina	Abdulla			Imperial College Healthcare NHS Trust		Consortium member	
Sarah	Darnell			Imperial College Healthcare NHS Trust		Consortium member	

Supplemental Online Content: Nonauthor Collaborators
*First name, last name, and suffix (if applicable) are required and will appear in PubMed.

***First Name and Middle Initial(s)**	***Last Name**	***Suffix (eg, Jr, III)**	Academic Degrees	Institution	Location (city, state/province, country)	Role or Contribution, eg, chair, principal investigator	Group (if more than 1 Group listed in the byline) and/or Subgroup (eg, Steering Committee)
Sobia	Mustafa			Imperial College Healthcare NHS Trust		Consortium member	
Pantelis	Georgiou			Imperial College London		Consortium member	
Jesus-Rodriguez	Manzano			Imperial College London		Consortium member	
Nicolas	Moser			Imperial College London		Consortium member	
Michael	Carter			Evelina London Children’s Hospital, Guy’s and St Thomas’ NHS		Consortium member	

				Foundation Trust; King’s College London			
Shane	Tibby			Evelina London Children’s Hospital, Guy’s and St Thomas’ NHS Foundation Trust; King’s College London		Consortium member	
Jonathan	Cohen			Evelina London Children’s Hospital, Guy’s and St Thomas’ NHS Foundation Trust; King’s College London		Consortium member	
Francesca	Davis			Evelina London Children’s Hospital, Guy’s and St Thomas’ NHS Foundation Trust; King’s College London		Consortium member	
Julia	Kenny			Evelina London Children’s Hospital, Guy’s and St Thomas’ NHS Foundation Trust; King’s College London		Consortium member	
Paul	Wellman			Evelina London Children’s Hospital, Guy’s and St Thomas’ NHS Foundation		Consortium member	

				Trust; King's College London			
Marie	White			Evelina London Children's Hospital, Guy's and St Thomas' NHS Foundation Trust; King's College London		Consortium member	

*First name, last name, and suffix (if applicable) are required and will appear in PubMed.

***First Name and Middle Initial(s)**	***Last Name**	***Suffix (eg, Jr, III)**	Academic Degrees	Institution	Location (city, state/province, country)	Role or Contribution, eg, chair, principal investigator	Group (if more than 1 Group listed in the byline) and/or Subgroup (eg, Steering Committee)
Matthew	Fish			Evelina London Children's Hospital, Guy's and St Thomas' NHS Foundation Trust; King's College London		Consortium member	
Aislinn	Jennings			Evelina London Children's Hospital, Guy's and St Thomas' NHS Foundation Trust; King's College London		Consortium member	
Manu	Shankar-Hari			Evelina London Children's		Consortium member	

				Hospital, Guy's and St Thomas' NHS Foundation Trust; King's College London			
Katy	Fidler			University Hospitals Sussex		Consortium member	
Dan	Agranoff			University Hospitals Sussex		Consortium member	
Vivien	Richmond			University Hospitals Sussex		Consortium member	
Mathhew	Seal			University Hospitals Sussex		Consortium member	
Saul	Faust			University Hospital Southampton NHS		Consortium member	
Dan	Owen			University Hospital Southampton NHS Foundation Trust		Consortium member	
Ruth	Ensom			University Hospital Southampton NHS Foundation Trust		Consortium member	
Sarah	McKay			University Hospital Southampton NHS Foundation Trust		Consortium member	
Mariya	Shaji			University Hospital Southampton NHS Foundation Trust		Consortium member	
Rachel	Schranz			University Hospital Southampton		Consortium member	

				NHS Foundation Trust			
Prita	Rughnani			Barts Health NHS Trust		Consortium member	
Amutha	Anpananthar			Barts Health NHS Trust		Consortium member	
Susan	Liebeschuetz			Barts Health NHS Trust		Consortium member	
Anna	Riddell			Barts Health NHS Trust		Consortium member	
Divya	Divakaran			Barts Health NHS Trust		Consortium member	
Louise	Han			Barts Health NHS Trust		Consortium member	
Nosheen	Khalid			Barts Health NHS Trust		Consortium member	
Ivone Lancoma	Malcolm			Barts Health NHS Trust		Consortium member	

*First name, last name, and suffix (if applicable) are required and will appear in PubMed.

***First Name and Middle Initial(s)**	***Last Name**	***Suffix (eg, Jr, III)**	Academic Degrees	Institution	Location (city, state/province, country)	Role or Contribution, eg, chair, principal investigator	Group (if more than 1 Group listed in the byline) and/or Subgroup (eg, Steering Committee)
Jessica	Schofield			Barts Health NHS Trust		Consortium member	
Teresa	Simagan			Barts Health NHS Trust		Consortium member	
Mark	Peters			Great Ormond Street Hospital for Children NHS Foundation Trust		Consortium member	
Alasdair	Bamford			Great Ormond Street Hospital for Children NHS		Consortium member	

				Foundation Trust			
Lauran	O'Neill			Great Ormond Street Hospital for Children NHS Foundation Trust		Consortium member	
Nazima	Pathan			Cambridge University Hospitals NHS Foundation Trust		Consortium member	
Esther	Daubney			Cambridge University Hospitals NHS Foundation Trust		Consortium member	
Deborah	White			Cambridge University Hospitals NHS Foundation Trust		Consortium member	
Melissa	Heightman			University College London Hospitals NHS Foundation Trust		Consortium member	
Sarah	Eisen			University College London Hospitals NHS Foundation Trust		Consortium member	
Terry	Segal			University College London Hospitals NHS Foundation Trust		Consortium member	
Lucy	Wellings			University College		Consortium member	

				London Hospitals NHS Foundation Trust			
Simon	B Drysdale			St George's University Hospitals NHS Foundation Trust		Consortium member	
Nicole	Branch			St George's University Hospitals NHS Foundation Trust		Consortium member	
Lisa	Hamzah			St George's University Hospitals NHS Foundation Trust		Consortium member	
Heather	Jarman			St George's University Hospitals NHS Foundation Trust		Consortium member	
Maggie	Nyirenda			Lewisham and Greenwich NHS Trust		Consortium member	
Lisa	Capozzi			Lewisham and Greenwich NHS Trust		Consortium member	
Emma	Gardiner			Lewisham and Greenwich NHS Trust		Consortium member	
Robert	Moots			Liverpool University Hospitals NHS Foundation Trust		Consortium member	

Supplemental Online Content: Nonauthor Collaborators
*First name, last name, and suffix (if applicable) are required and will appear in PubMed.

***First Name and Middle Initial(s)**	***Last Name**	***Suffix (eg, Jr, III)**	Academic Degrees	Institution	Location (city, state/province, country)	Role or Contribution, eg, chair, principal investigator	Group (if more than 1 Group listed in the byline) and/or Subgroup (eg, Steering Committee)
Magda	Nasher			Liverpool University Hospitals NHS Foundation Trust		Consortium member	
Anita	Hanson			Liverpool University Hospitals NHS Foundation Trust		Consortium member	
Michelle	Linforth			Liverpool University Hospitals NHS Foundation Trust		Consortium member	
Sean	O'Riordan			Leeds Teaching Hospitals NHS Trust		Consortium member	
Donna	Ellis			Leeds Teaching Hospitals NHS Trust		Consortium member	
Akash	Deep			King's College Hospital NHS Foundation Trust		Consortium member	
Ivan	Caro			King's College Hospital NHS Foundation Trust		Consortium member	
Fiona	Shackley			Sheffield Children's NHS Foundation Trust		Consortium member	
Arianna	Bellini			Sheffield Children's NHS		Consortium member	

				Foundation Trust			
Stuart	Gormley			Sheffield Children’s NHS Foundation Trust		Consortium member	
Samira	Neshat			University Hospitals of Leicester NHS Foundation Trust		Consortium member	
Barnaby	Scholefield			Birmingham Women’s and Children’s Hospital NHS Foundation Trust		Consortium member	
Ceri	Robbins			Birmingham Women’s and Children’s Hospital NHS Foundation Trust		Consortium member	
Helen	Winmill			Birmingham Women’s and Children’s Hospital NHS Foundation Trust		Consortium member	
Stéphane C	Paulus			University of Oxford Partner Children’s Hospital, John Radcliffe Hospital		Consortium member	
Andrew J	Pollard			University of Oxford Partner Children’s		Consortium member	
Mark	Anthony			University of Oxford Partner Children’s Hospital, John Radcliffe Hospital		Consortium member	
Sarah	Hopton			University of Oxford		Consortium member	

				Partner Children’s Hospital, John Radcliffe Hospital			

*First name, last name, and suffix (if applicable) are required and will appear in PubMed.

***First Name and Middle Initial(s)**	***Last Name**	***Suffix (eg, Jr, III)**	Academic Degrees	Institution	Location (city, state/province, country)	Role or Contribution, eg, chair, principal investigator	Group (if more than 1 Group listed in the byline) and/or Subgroup (eg, Steering Committee)
Danielle	Miller			University of Oxford Partner Children’s Hospital, John Radcliffe Hospital		Consortium member	
Zoe	Oliver			University of Oxford Partner Children’s Hospital, John Radcliffe Hospital		Consortium member	
Sally	Beer			University of Oxford Partner Children’s Hospital, John Radcliffe Hospital		Consortium member	
Bryony	Ward			University of Oxford Partner Children’s Hospital, John Radcliffe Hospital		Consortium member	
Shrijana	Shrestha			University of Oxford, Nepal Site		Consortium member	

Meeru	Gurung			University of Oxford, Nepal Site		Consortium member	
Puja	Amatya			University of Oxford, Nepal Site		Consortium member	
Bhishma	Pokhrel			University of Oxford, Nepal Site		Consortium member	
Sanjeev Man	Bijukchhe			University of Oxford, Nepal Site		Consortium member	
Madhav Chandra	Gautam			University of Oxford, Nepal Site		Consortium member	
Sarah	Kelly			University of Oxford, Nepal Site		Consortium member	
Peter	O'Reilly			University of Oxford, Nepal Site		Consortium member	
Sonu	Shrestha			University of Oxford, Nepal Site		Consortium member	
Federico	Martinón-Torres			Hospital Clínico Universitario de Santiago, Santiago de Compostela		Consortium member	
Antonio	Salas			Hospital Clínico Universitario de Santiago, Santiago de Compostela		Consortium member	
Fernando Álvez	González			Hospital Clínico Universitario de Santiago, Santiago de Compostela		Consortium member	
Sonia Ares	Gómez			Hospital Clínico Universitario de Santiago, Santiago de Compostela		Consortium member	
Xabier	Bello			Hospital Clínico		Consortium member	

				Universitario de Santiago, Santiago de Compostela			
Mirian Ben	García			Hospital Clínico Universitario de Santiago, Santiago de Compostela		Consortium member	
Fernando Caamaño	Viña			Hospital Clínico Universitario de Santiago, Santiago de Compostela		Consortium member	

*First name, last name, and suffix (if applicable) are required and will appear in PubMed.

***First Name and Middle Initial(s)**	***Last Name**	***Suffix (eg, Jr, III)**	Academic Degrees	Institution	Location (city, state/province, country)	Role or Contribution, eg, chair, principal investigator	Group (if more than 1 Group listed in the byline) and/or Subgroup (eg, Steering Committee)
Sandra	Carnota			Hospital Clínico Universitario de Santiago, Santiago de Compostela		Consortium member	
María José	Curras-Tuala			Hospital Clínico Universitario de Santiago, Santiago de Compostela		Consortium member	
Ana Dacosta	Urbieta			Hospital Clínico Universitario de Santiago, Santiago de Compostela		Consortium member	
Carlos Durán	Suárez			Hospital Clínico		Consortium member	

				Universitario de Santiago, Santiago de Compostela			
Isabel Ferreiros	Vidal			Hospital Clínico Universitario de Santiago, Santiago de Compostela		Consortium member	
Luisa García	Vicente			Hospital Clínico Universitario de Santiago, Santiago de Compostela		Consortium member	
Alberto	Gómez-Carballa			Hospital Clínico Universitario de Santiago, Santiago de Compostela		Consortium member	
Jose Gómez	Rial			Hospital Clínico Universitario de Santiago, Santiago de Compostela		Consortium member	
Pilar Leboráns	Iglesias			Hospital Clínico Universitario de Santiago, Santiago de Compostela		Consortium member	
Narmeen	Mallah			Hospital Clínico Universitario de Santiago, Santiago de Compostela		Consortium member	
Nazareth	Martinón-Torres			Hospital Clínico Universitario de Santiago, Santiago de Compostela		Consortium member	
José María	Martinón			Hospital Clínico Universitario de Santiago,		Consortium member	

				Santiago de Compostela			
Belén Mosquera Pérez	Sánchez			Hospital Clínico Universitario de Santiago, Santiago de Compostela		Consortium member	
Jacobo	Pardo-Seco			Hospital Clínico Universitario de Santiago, Santiago de Compostela		Consortium member	
Sara	Pischedda			Hospital Clínico Universitario de Santiago, Santiago de Compostela		Consortium member	
Sara Rey	Vázquez			Hospital Clínico Universitario de Santiago, Santiago de Compostela		Consortium member	
Irene Rivero	Calle			Hospital Clínico Universitario de Santiago, Santiago de Compostela		Consortium member	
Carmen	Rodríguez-Tenreiro			Hospital Clínico Universitario de Santiago, Santiago de Compostela		Consortium member	

*First name, last name, and suffix (if applicable) are required and will appear in PubMed.

***First Name and Middle Initial(s)**	***Last Name**	***Suffix (eg, Jr, III)**	Academic Degrees	Institution	Location (city, state/province, country)	Role or Contribution, eg, chair, principal investigator	Group (if more than 1 Group listed in the byline) and/or Subgroup (eg,

							Steering Committee)
Lorenzo	Redondo-Collazo			Hospital Clínico Universitario de Santiago, Santiago de Compostela		Consortium member	
Antonio	Salas			Hospital Clínico Universitario de Santiago, Santiago de Compostela		Consortium member	
Sonia Serén	Fernández			Hospital Clínico Universitario de Santiago, Santiago de Compostela		Consortium member	
Marisol Vilas	Iglesias			Hospital Clínico Universitario de Santiago, Santiago de Compostela		Consortium member	
Enitan D	Carrol			University of Liverpool Institute of Infection, Veterinary and Ecological Sciences; Alder Hey Children's Hospital, Liverpool		Consortium member	
Elizabeth	Cocklin			University of Liverpool Institute of Infection, Veterinary and Ecological Sciences; Alder Hey Children's Hospital, Liverpool		Consortium member	
Rebecca	Beckley			University of Liverpool Institute of Infection, Veterinary and Ecological Sciences; Alder Hey Children's		Consortium member	

				Hospital, Liverpool			
Abbey	Bracken			University of Liverpool Institute of Infection, Veterinary and Ecological Sciences; Alder Hey Children's Hospital, Liverpool		Consortium member	
Ceri	Evans			University of Liverpool Institute of Infection, Veterinary and Ecological Sciences; Alder Hey Children's Hospital, Liverpool		Consortium member	
Aakash	Khanijau			University of Liverpool Institute of Infection, Veterinary and Ecological Sciences; Alder Hey Children's Hospital, Liverpool		Consortium member	
Rebecca	Lenihan			University of Liverpool Institute of Infection, Veterinary and Ecological Sciences; Alder Hey Children's Hospital, Liverpool		Consortium member	

*First Name and Middle Initial(s)	*Last Name	*Suffix (eg, Jr, III)	Academic Degrees	Institution	Location (city, state/province, country)	Role or Contribution, eg, chair, principal investigator	Group (if more than 1 Group listed in the byline) and/or Subgroup (eg, Steering Committee)
Nadia	Lewis-Burke			University of Liverpool Institute of Infection, Veterinary and Ecological Sciences; Alder Hey Children's Hospital, Liverpool		Consortium member	
Karen	Newall			Alder Hey Children's Hospital, Liverpool		Consortium member	
Sam	Romaine			Alder Hey Children's Hospital, Liverpool		Consortium member	
Jennifer	Whitbread			Alder Hey Children's Hospital, Liverpool		Consortium member	
Maria	Tsolia			National and Kapodistrian University of Athens		Consortium member	
Irini	Eleftheriou			National and Kapodistrian University of Athens		Consortium member	
Nikos	Spyridis			National and Kapodistrian University of Athens		Consortium member	
Maria	Tambouratzi			National and Kapodistrian University of Athens		Consortium member	

Despoina	Maritsi			National and Kapodistrian University of Athens		Consortium member	
Antonios	Marmarinos			National and Kapodistrian University of Athens		Consortium member	
Marietta	Xagorari			National and Kapodistrian University of Athens		Consortium member	
Lourida	Panagiota			National and Kapodistrian University of Athens		Consortium member	
Pefanis	Aggelos			National and Kapodistrian University of Athens		Consortium member	
Akinosoglou	Karolina			National and Kapodistrian University of Athens		Consortium member	
Gogos	Charalambos			National and Kapodistrian University of Athens		Consortium member	
Maragos	Markos			National and Kapodistrian University of Athens		Consortium member	
Voulgarelis	Michalis			National and Kapodistrian University of Athens		Consortium member	

Supplemental Online Content: Nonauthor Collaborators
*First name, last name, and suffix (if applicable) are required and will appear in PubMed.

***First Name and Middle Initial(s)**	***Last Name**	***Suffix (eg, Jr, III)**	Academic Degrees	Institution	Location (city, state/province, country)	Role or Contribution, eg, chair, principal investigator	Group (if more than 1 Group listed in the byline) and/or Subgroup (eg, Steering Committee)

Stergiou	Ioanna			National and Kapodistrian University of Athens		Consortium member	
Marieke	Emonts			Newcastle upon Tyne Hospitals NHS Foundation Trust and Newcastle University (UK)		Consortium member	
Emma	Lim			Newcastle upon Tyne Hospitals NHS Foundation Trust and Newcastle University (UK)		Consortium member	
John	Isaacs			Newcastle upon Tyne Hospitals NHS Foundation Trust and Newcastle University (UK)		Consortium member	
Kathryn	Bell			Newcastle upon Tyne Hospitals NHS Foundation Trust and Newcastle University (UK)		Consortium member	
Stephen	Crulley			Newcastle upon Tyne Hospitals NHS Foundation Trust and Newcastle University (UK)		Consortium member	
Daniel	Fabian			Newcastle upon Tyne Hospitals NHS Foundation Trust and Newcastle University (UK)		Consortium member	
Evelyn	Thomson			Newcastle upon Tyne Hospitals NHS Foundation		Consortium member	

				Trust and Newcastle University (UK)			
Diane	Wallia			Newcastle upon Tyne Hospitals NHS Foundation Trust and Newcastle University (UK)		Consortium member	
Caroline	Miller			Newcastle upon Tyne Hospitals NHS Foundation Trust and Newcastle University (UK)		Consortium member	
Ashley	Bell			Newcastle upon Tyne Hospitals NHS Foundation Trust and Newcastle University (UK)		Consortium member	
Fabian J S	van der Velden			Newcastle upon Tyne Hospitals NHS Foundation Trust and Newcastle University (UK)		Consortium member	

*First name, last name, and suffix (if applicable) are required and will appear in PubMed.

***First Name and Middle Initial(s)**	***Last Name**	***Suffix (eg, Jr, III)**	Academic Degrees	Institution	Location (city, state/province, country)	Role or Contribution, eg, chair, principal investigator	Group (if more than 1 Group listed in the byline) and/or Subgroup (eg, Steering Committee)
Geoff	Shenton			Newcastle upon Tyne Hospitals NHS Foundation Trust and		Consortium member	

				Newcastle University (UK)			
Ashley	Price			Newcastle upon Tyne Hospitals NHS Foundation Trust and Newcastle University (UK)		Consortium member	
Owen	Treloar			Newcastle upon Tyne Hospitals NHS Foundation Trust and Newcastle University (UK)		Consortium member	
Daisy	Thomas			Newcastle upon Tyne Hospitals NHS Foundation Trust and Newcastle University (UK)		Consortium member	
Pablo	Rojo			Servicio Madrileño de Salud (SERMAS) - Fundación Biomédica del Hospital Universitario 12 de Octubre (FIB-H12O)		Consortium member	
Cristina	Epalza			Servicio Madrileño de Salud (SERMAS) - Fundación Biomédica del Hospital Universitario 12 de Octubre (FIB-H12O)		Consortium member	
Serena	Villaverde			Servicio Madrileño de Salud (SERMAS) - Fundación Biomédica del Hospital Universitario 12 de Octubre (FIB-H12O)		Consortium member	

Sonia	Márquez			Servicio Madrileño de Salud (SERMAS) - Fundación Biomédica del Hospital Universitario 12 de Octubre (FIB-H12O)		Consortium member	
Manuel	Gijón			Servicio Madrileño de Salud (SERMAS) - Fundación Biomédica del Hospital Universitario 12 de Octubre (FIB-H12O)		Consortium member	
Fátima	Machín			Servicio Madrileño de Salud (SERMAS) - Fundación Biomédica del Hospital Universitario 12 de Octubre (FIB-H12O)		Consortium member	

*First name, last name, and suffix (if applicable) are required and will appear in PubMed.

***First Name and Middle Initial(s)**	***Last Name**	***Suffix (eg, Jr, III)**	Academic Degrees	Institution	Location (city, state/province, country)	Role or Contribution, eg, chair, principal investigator	Group (if more than 1 Group listed in the byline) and/or Subgroup (eg, Steering Committee)
Laura	Cabello			Servicio Madrileño de Salud (SERMAS) - Fundación Biomédica del Hospital Universitario 12 de Octubre (FIB-H12O)		Consortium member	

Irene	Hernández			Servicio Madrileño de Salud (SERMAS) - Fundación Biomédica del Hospital Universitario 12 de Octubre (FIB-H12O)		Consortium member	
Lourdes	Gutiérrez			Servicio Madrileño de Salud (SERMAS) - Fundación Biomédica del Hospital Universitario 12 de Octubre (FIB-H12O)		Consortium member	
Ángela	Manzanares			Servicio Madrileño de Salud (SERMAS) - Fundación Biomédica del Hospital Universitario 12 de Octubre (FIB-H12O)		Consortium member	
Taco W	Kuijpers			Amsterdam University Medical Center		Consortium member	
Martijn	van de Kuip			Amsterdam University Medical Center		Consortium member	
Marceline	van Furth			Amsterdam University Medical Center		Consortium member	
Merlijn	van den Berg			Amsterdam University Medical Center		Consortium member	
Giske	Biesbroek			Amsterdam University Medical Center		Consortium member	

Floris	Verkuil			Amsterdam University Medical Center		Consortium member	
Carlijn	van der Zee			Amsterdam University Medical Center		Consortium member	
Dasja	Pajkrt			Amsterdam University Medical Center		Consortium member	
Michael Boele	van Hensbroek			Amsterdam University Medical Center		Consortium member	
Dieneke	Schonenberg			Amsterdam University Medical Center		Consortium member	

*First name, last name, and suffix (if applicable) are required and will appear in PubMed.

***First Name and Middle Initial(s)**	***Last Name**	***Suffix (eg, Jr, III)**	Academic Degrees	Institution	Location (city, state/province, country)	Role or Contribution, eg, chair, principal investigator	Group (if more than 1 Group listed in the byline) and/or Subgroup (eg, Steering Committee)
Mariken	Gruppen			Amsterdam University Medical Center		Consortium member	
Sietse	Nagelkerke			Amsterdam University Medical Center		Consortium member	
MachielH	Jansen			Amsterdam University Medical Center		Consortium member	
Ines	Goetschalckx			Amsterdam University Medical Center		Consortium member	

Lorenza	Romani			Bambino Gesù Children’s Hospital, Rome		Consortium member	
Maia	De Luca			Bambino Gesù Children’s Hospital, Rome		Consortium member	
Sara	Chiurchiù			Bambino Gesù Children’s Hospital, Rome		Consortium member	
Costanza	Tripiciano			Bambino Gesù Children’s Hospital, Rome		Consortium member	
Stefania	Mercadante			Bambino Gesù Children’s Hospital, Rome		Consortium member	
Clementien L	Vermont			ERASMUS MC-Sophia Children’s Hospital		Consortium member	
Henriëtte A	Moll			ERASMUS MC-Sophia Children’s Hospital		Consortium member	
Dorine M	Borensztajn			ERASMUS MC-Sophia Children’s Hospital		Consortium member	
Nienke N	Hagedoorn			ERASMUS MC-Sophia Children’s Hospital		Consortium member	
Chantal	Tan			ERASMUS MC-Sophia Children’s Hospital		Consortium member	
Joany	Zachariasse			ERASMUS MC-Sophia Children’s Hospital		Consortium member	

W	Dik			ERASMUS MC-Sophia Children's Hospital		Consortium member	
Ching-Fen	Shen			National Cheng Kung University Hospital, College of Medicine, National Cheng Kung University, Taiwan		Consortium member	

*First name, last name, and suffix (if applicable) are required and will appear in PubMed.

***First Name and Middle Initial(s)**	***Last Name**	***Suffix (eg, Jr, III)**	Academic Degrees	Institution	Location (city, state/province, country)	Role or Contribution, eg, chair, principal investigator	Group (if more than 1 Group listed in the byline) and/or Subgroup (eg, Steering Committee)
Dace	Zavadska			Riga Stradins University		Consortium member	
Sniedze	Laivacuma			Riga Stradins University		Consortium member	
Aleksandra	Rudzate			Riga Stradins University		Consortium member	
Diana	Stoldere			Riga Stradins University		Consortium member	
Arta	Barzdina			Riga Stradins University		Consortium member	
Elza	Barzdina			Riga Stradins University		Consortium member	
Sniedze	Laivacuma			Riga Stradins University		Consortium member	
Monta	Madelane			Riga Stradins University		Consortium member	
Dagne	Gravele			Children clinical university hospital, Riga		Consortium member	

Dace	Svilz			Children clinical university hospital, Riga		Consortium member	
Romain	Basmaci			Service de Pédiatrie-Urgences, AP-HP, Hôpital Louis-Mourier, F-92700 Colombes, France		Consortium member	
Noémie	Lachaume			Service de Pédiatrie-Urgences, AP-HP, Hôpital Louis-Mourier, F-92700 Colombes, France		Consortium member	
Pauline	Bories			Service de Pédiatrie-Urgences, AP-HP, Hôpital Louis-Mourier, F-92700 Colombes, France		Consortium member	
RajaBen	Tkhayat			Service de Pédiatrie-Urgences, AP-HP, Hôpital Louis-Mourier, F-92700 Colombes, France		Consortium member	
Laura	Chériaux			Service de Pédiatrie-Urgences, AP-HP, Hôpital Louis-		Consortium member	

				Mourier, F-92700 Colombes, France			
Juraté	Davoust			Service de Pédiatrie-Urgences, AP-HP, Hôpital Louis-Mourier, F-92700 Colombes, France		Consortium member	
Kim-Thanh	Ong			Service de Pédiatrie-Urgences, AP-HP, Hôpital Louis-Mourier, F-92700 Colombes, France		Consortium member	
Marie	Cotillon			Service de Pédiatrie-Urgences, AP-HP, Hôpital Louis-Mourier, F-92700 Colombes, France		Consortium member	

***First Name and Middle Initial(s)**	***Last Name**	***Suffix (eg, Jr, III)**	Academic Degrees	Institution	Location (city, state/province, country)	Role or Contribution, eg, chair, principal investigator	Group (if more than 1 Group listed in the byline) and/or Subgroup (eg, Steering Committee)
Thibault	de Groc			Service de Pédiatrie-		Consortium member	

				Urgences, AP-HP, Hôpital Louis-Mourier, F-92700 Colombes, France			
Sébastien	Le			Service de Pédiatrie-Urgences, AP-HP, Hôpital Louis-Mourier, F-92700 Colombes, France		Consortium member	
Nathalie	Vergnault			Service de Pédiatrie-Urgences, AP-HP, Hôpital Louis-Mourier, F-92700 Colombes, France		Consortium member	
Hélène	Sée			Service de Pédiatrie-Urgences, AP-HP, Hôpital Louis-Mourier, F-92700 Colombes, France		Consortium member	
Laure	Cohen			Service de Pédiatrie-Urgences, AP-HP, Hôpital Louis-Mourier, F-92700 Colombes, France		Consortium member	
Alice	de Tugny			Service de Pédiatrie-Urgences, AP-HP, Hôpital Louis-Mourier, F-92700 Colombes, France		Consortium member	
Nevena	Danekova			Service de Pédiatrie-Urgences, AP-HP, Hôpital Louis-Mourier,		Consortium member	

				F-92700 Colombes, France			
Marine	Mommert-Tripon			BioMérieux - Open Innovation & Partnerships Department, Lyon, Franceérieux		Consortium member	
Karen	Brengel-Pesce			BioMérieux - Open Innovation & Partnerships Department, Lyon, Franceérieux		Consortium member	
Marko	Pokorn			University Medical Centre Ljubljana, Slovenia		Consortium member	
Mojca	Kolnik			University Medical Centre Ljubljana, Slovenia		Consortium member	
Tadej	Avčin			University Medical Centre Ljubljana, Slovenia		Consortium member	
Tanja	Avramoska			University Medical Centre Ljubljana, Slovenia		Consortium member	

Supplemental Online Content: Nonauthor Collaborators
*First name, last name, and suffix (if applicable) are required and will appear in PubMed.

***First Name and Middle Initial(s)**	***Last Name**	***Suffix (eg, Jr, III)**	Academic Degrees	Institution	Location (city, state/province, country)	Role or Contribution, eg, chair, principal investigator	Group (if more than 1 Group listed in the byline) and/or Subgroup (eg,

							Steering Committee)
Natalija	Bahovec			University Medical Centre Ljubljana, Slovenia		Consortium member	
Petra	Bogovič			University Medical Centre Ljubljana, Slovenia		Consortium member	
Lidija	Kitanovski			University Medical Centre Ljubljana, Slovenia		Consortium member	
Mirijam	Nahtigal			University Medical Centre Ljubljana, Slovenia		Consortium member	
Lea	Papst			University Medical Centre Ljubljana, Slovenia		Consortium member	
Tina	Plankar Srovin			University Medical Centre Ljubljana, Slovenia		Consortium member	
Franc	Strle			University Medical Centre Ljubljana, Slovenia		Consortium member	
Katarina	Vincek			University Medical Centre Ljubljana, Slovenia		Consortium member	
Michiel	van der Flier			University Medical Center Utrecht, Utrecht		Consortium member	
Wim J E	Tissing			University Medical Center		Consortium member	

				Utrecht, Utrecht			
Roelie M	Wösten-van Asperen			University Medical Center Utrecht, Utrecht		Consortium member	
Sebastiaan J	Vastert			University Medical Center Utrecht, Utrecht		Consortium member	
Daniel C	Vijlbrief			University Medical Center Utrecht, Utrecht		Consortium member	
Louis J	Bont			University Medical Center Utrecht, Utrecht		Consortium member	
Coco R	Beudeker			University Medical Center Utrecht, Utrecht		Consortium member	
Philipp	Agyeman			Inselspital, Bern University Hospital, University of Bern, Switzerland		Consortium member	
Luregn	Schlapbach			Inselspital, Bern University Hospital, University of Bern, Switzerland		Consortium member	
Christoph	Aebi			Inselspital, Bern University Hospital, University of Bern, Switzerland		Consortium member	

Supplemental Online Content: Nonauthor Collaborators
*First name, last name, and suffix (if applicable) are required and will appear in PubMed.

***First Name and Middle Initial(s)**	***Last Name**	***Suffix (eg, Jr, III)**	Academic Degrees	Institution	Location (city, state/province, country)	Role or Contribution, eg, chair, principal investigator	Group (if more than 1 Group listed in the byline) and/or Subgroup (eg, Steering Committee)
Mariama	Usman			Inselspital, Bern University Hospital, University of Bern, Switzerland		Consortium member	
Stefanie	Schlüchter			Inselspital, Bern University Hospital, University of Bern, Switzerland		Consortium member	
Verena	Wyss			Inselspital, Bern University Hospital, University of Bern, Switzerland		Consortium member	
Nina	Schöbi			Inselspital, Bern University Hospital, University of Bern, Switzerland		Consortium member	
Elisa	Zimmermann			Inselspital, Bern University Hospital, University of Bern, Switzerland		Consortium member	
Marion	Meier			Inselspital, Bern University Hospital, University of Bern, Switzerland		Consortium member	
Kathrin	Weber			Inselspital, Bern University		Consortium member	

				Hospital, University of Bern, Switzerland			
Colin	Fink			Micropathology Ltd, The Venture Center, University of Warwick Science Park, Sir William Lyons Road, Coventry		Consortium member	
Marie	Voice			Micropathology Ltd, The Venture Center, University of Warwick Science Park, Sir William Lyons Road, Coventry		Consortium member	
Leo	Calvo-Bado			Micropathology Ltd, The Venture Center, University of Warwick Science Park, Sir William Lyons Road, Coventry		Consortium member	
Michael	Steele			Micropathology Ltd, The Venture Center, University of Warwick Science Park, Sir William Lyons Road, Coventry		Consortium member	
Jennifer	Holden			Micropathology Ltd, The Venture Center, University of Warwick Science Park, Sir William Lyons Road, Coventry		Consortium member	

*First Name and Middle Initial(s)	*Last Name	*Suffix (eg, Jr, III)	Academic Degrees	Institution	Location (city, state/province, country)	Role or Contribution, eg, chair, principal investigator	Group (if more than 1 Group listed in the byline) and/or Subgroup (eg, Steering Committee)
Andrew	Taylor			Micropathology Ltd, The Venture Center, University of Warwick Science Park, Sir William Lyons Road, Coventry		Consortium member	
Ronan	Calvez			Micropathology Ltd, The Venture Center, University of Warwick Science Park, Sir William Lyons Road, Coventry		Consortium member	
Catherine	Davies			Micropathology Ltd, The Venture Center, University of Warwick Science Park, Sir William Lyons Road, Coventry		Consortium member	
Benjamin	Evans			Micropathology Ltd, The Venture Center, University of Warwick Science Park, Sir William Lyons Road, Coventry		Consortium member	
Jake	Stevens			Micropathology Ltd, The Venture Center, University of Warwick Science Park, Sir William Lyons Road, Coventry		Consortium member	

Peter	Matthews			Micropathology Ltd, The Venture Center, University of Warwick Science Park, Sir William Lyons Road, Coventry		Consortium member	
Kyle	Billing			Micropathology Ltd, The Venture Center, University of Warwick Science Park, Sir William Lyons Road, Coventry		Consortium member	
Werner	Zenz			Medical University of Graz, Austria		Consortium member	
Alexander	Binder			Medical University of Graz, Austria		Consortium member	
Benno	Kohlmaier			Medical University of Graz, Austria		Consortium member	
Daniela S	Kohlfürst			Medical University of Graz, Austria		Consortium member	
Nina A	Schweintzger			Medical University of Graz, Austria		Consortium member	
Christoph	Zurl			Medical University of Graz, Austria		Consortium member	
Susanne	Hösele			Medical University of Graz, Austria		Consortium member	
Manuel	Leitner			Medical University of Graz, Austria		Consortium member	

*First name, last name, and suffix (if applicable) are required and will appear in PubMed.

*First Name and Middle Initial(s)	*Last Name	*Suffix (eg, Jr, III)	Academic Degrees	Institution	Location (city, state/province, country)	Role or Contribution, eg, chair,	Group (if more than 1 Group listed in the

						principal investigator	byline) and/or Subgroup (eg, Steering Committee)
Lena	Pölz			Medical University of Graz, Austria		Consortium member	
Alexandra	Rusu			Medical University of Graz, Austria		Consortium member	
Glorija	Rajic			Medical University of Graz, Austria		Consortium member	
Bianca	Stoiser			Medical University of Graz, Austria		Consortium member	
Martina	Strempfl			Medical University of Graz, Austria		Consortium member	
Manfred G	Sagmeister			Medical University of Graz, Austria		Consortium member	
Sebastian	Bauchinger			Medical University of Graz, Austria		Consortium member	
Martin	Benesch			Medical University of Graz, Austria		Consortium member	
Astrid	Ceolotto			Medical University of Graz, Austria		Consortium member	
Ernst	Eber			Medical University of Graz, Austria		Consortium member	
Siegfried	Gallistl			Medical University of Graz, Austria		Consortium member	
Harald	Haidl			Medical University of Graz, Austria		Consortium member	
Almuthe	Hauer			Medical University of Graz, Austria		Consortium member	
Christa	Hude			Medical University of Graz, Austria		Consortium member	

Andreas	Kapper			Medical University of Graz, Austria		Consortium member	
Markus	Keldorfer			Medical University of Graz, Austria		Consortium member	
Sabine	Löffler			Medical University of Graz, Austria		Consortium member	
Tobias	Niedrist			Medical University of Graz, Austria		Consortium member	
Heidemari e	Pilch			Medical University of Graz, Austria		Consortium member	
Andreas	Pfleger			Medical University of Graz, Austria		Consortium member	
Klaus	Pfurtscheller			Medical University of Graz, Austria		Consortium member	
Siegfried	Rödl			Medical University of Graz, Austria		Consortium member	
Andrea	Skrabl-Baumgartne r			Medical University of Graz, Austria		Consortium member	
Volker	Strenger			Medical University of Graz, Austria		Consortium member	
Elmar	Wallner			Medical University of Graz, Austria		Consortium member	
Maike K	Tauchert			Biobanking and BioMolecular Resources Research Infrastructure - European Research Infrastructure Consortium (BBMRI-ERIC), Neue Stiftingtalstrass e 2/B/6, 8010, Graz, Austria		Consortium member	
Shunmay	Yeung			London School of Hygiene and		Consortium member	

				Tropical Medicine			
Manuel	Dewez			London School of Hygiene and Tropical Medicine		Consortium member	

Supplemental Online Content: Nonauthor Collaborators
*First name, last name, and suffix (if applicable) are required and will appear in PubMed.

***First Name and Middle Initial(s)**	***Last Name**	***Suffix (eg, Jr, III)**	Academic Degrees	Institution	Location (city, state/province, country)	Role or Contribution, eg, chair, principal investigator	Group (if more than 1 Group listed in the byline) and/or Subgroup (eg, Steering Committee)
David	Bath			London School of Hygiene and Tropical Medicine		Consortium member	
Elizabeth	Fitchett			London School of Hygiene and Tropical Medicine		Consortium member	
Fiona	Cresswell			London School of Hygiene and Tropical Medicine		Consortium member	
Effua	Usuf			Medical Research Council Unit The Gambia at LSHTM		Consortium member	
Kalifa	Bojang			Medical Research Council Unit The Gambia at LSHTM		Consortium member	
Anna	Roca			Medical Research		Consortium member	

				Council Unit The Gambia at LSHTM			
Isatou	Sarr			Medical Research Council Unit The Gambia at LSHTM		Consortium member	
Momodou	Saidykhan			Medical Research Council Unit The Gambia at LSHTM		Consortium member	
Ebrahim	Ndure			Medical Research Council Unit The Gambia at LSHTM		Consortium member	
Ulrich	von Both			Dr. von Hauner Children’s Hospital, University Hospital, LMU Munich		Consortium member	
Laura	Kolberg			Dr. von Hauner Children’s Hospital, University Hospital, LMU Munich		Consortium member	
Patricia	Schmied			Dr. von Hauner Children’s Hospital, University Hospital, LMU Munich		Consortium member	
Ioanna	Mavridi			Dr. von Hauner Children’s Hospital, University Hospital, LMU Munich		Consortium member	

Irene	Alba-Alejandre			University Hospital, LMU Munich, Munich, Germany		Consortium member	
Nikolaus	Haas			University Hospital, LMU Munich,		Consortium member	
Esther	Maier			Dr. von Hauner Children's Hospital, University Hospital, LMU Munich		Consortium member	
Sabrina	Juranek			Dr. von Hauner Children's Hospital, University Hospital, LMU Munich		Consortium member	
Tobias	Feuchtinger			Dr. von Hauner Children's Hospital, University Hospital, LMU Munich		Consortium member	

Supplemental Online Content: Nonauthor Collaborators
*First name, last name, and suffix (if applicable) are required and will appear in PubMed.

***First Name and Middle Initial(s)**	***Last Name**	***Suffix (eg, Jr, III)**	Academic Degrees	Institution	Location (city, state/province, country)	Role or Contribution, eg, chair, principal investigator	Group (if more than 1 Group listed in the byline) and/or Subgroup (eg, Steering Committee)
Katharina	Danhauser			Dr. von Hauner		Consortium member	

				Children's Hospital, University Hospital, LMU Munich			
Matthias	Griese			Dr. von Hauner Children's Hospital, University Hospital, LMU Munich		Consortium member	
Matthias	Kappler			Dr. von Hauner Children's Hospital, University Hospital, LMU Munich		Consortium member	
Eberhard	Lurz			Dr. von Hauner Children's Hospital, University Hospital, LMU Munich		Consortium member	
Sebastian	Schroepf			Dr. von Hauner Children's Hospital, University Hospital, LMU Munich		Consortium member	
Florian	Hoffmann			Dr. von Hauner Children's Hospital, University Hospital, LMU Munich		Consortium member	
Karl	Reiter			Dr. von Hauner Children's Hospital, University Hospital, LMU Munich		Consortium member	
Carola	Schoen			Dr. von Hauner Children's		Consortium member	

				Hospital, University Hospital, LMU Munich			
Philipp	Agyeman			Bern University Hospital, University of Bern, Switzerland		Consortium member	
Christoph	Aebi			Bern University Hospital, University of Bern, Switzerland		Consortium member	
Luregn J	Schlapbach			University Children`s Hospital Zurich		Consortium member	
Eric	Giannoni			Lausanne University Hospital and University of Lausanne, Switzerland		Consortium member	
Martin	Stocker			Children’s Hospital Lucerne, Switzerland		Consortium member	
Klara M	Posfay-Barbe			Children’s Hospital of Geneva, University Hospitals of Geneva, Switzerland		Consortium member	
Ulrich	Heininger			University of Basel Children’s Hospital, Switzerland		Consortium member	
Sara	Bernhard-Stirnemann			Children’s Hospital Aarau, Switzerland		Consortium member	
Anita	Niederer-Loher			Children’s Hospital of Eastern Switzerland		Consortium member	

				St. Gallen, Switzerland			
Christian	Kahlert			Children's Hospital of Eastern Switzerland St. Gallen, Switzerland		Consortium member	

Supplemental Online Content: Nonauthor Collaborators
*First name, last name, and suffix (if applicable) are required and will appear in PubMed.

***First Name and Middle Initial(s)**	***Last Name**	***Suffix (eg, Jr, III)**	Academic Degrees	Institution	Location (city, state/province, country)	Role or Contribution, eg, chair, principal investigator	Group (if more than 1 Group listed in the byline) and/or Subgroup (eg, Steering Committee)
Giancarlo	Natalucci			University Hospital Zurich, Switzerland		Consortium member	
Christa	Relly			Children's Research Center, University Children's Hospital Zurich, Switzerland		Consortium member	
Christoph	Berger			Children's Research Center, University Children's Hospital Zurich, Switzerland		Consortium member	
Thomas	Riedel			Children's Hospital Chur, Switzerland		Consortium member	
