# Supplementary material for: Shared neutrophil and T cell dysfunction is accompanied by a distinct interferon signature during severe febrile illnesses in children
Source: Nat Commun. 2024 Sep 19;15:8224. doi: 10.1038/s41467-024-52246-0 (PMC11413185; doi:10.1038/s41467-024-52246-0)
Supplement: Supplementary file 3 — Reporting Summary [file 41467_2024_52246_MOESM3_ESM.pdf]

nature portfolio

Corresponding author(s): Manu Shankar-Hari
Michael Levin

Last updated by author(s): Jul 31, 2024

Reporting Summary

Nature Portfolio wishes to improve the reproducibility of the work that we publish. This form provides structure for consistency and transparency in reporting. For further information on Nature Portfolio policies, see our Editorial Policies and the Editorial Policy Checklist.

Statistics

For all statistical analyses, confirm that the following items are present in the figure legend, table legend, main text, or Methods section.

n/a	Confirmed	
☐	☒	The exact sample size (*n*) for each experimental group/condition, given as a discrete number and unit of measurement
☐	☒	A statement on whether measurements were taken from distinct samples or whether the same sample was measured repeatedly
☐	☒	The statistical test(s) used AND whether they are one- or two-sided<br>*Only common tests should be described solely by name; describe more complex techniques in the Methods section.*
☐	☒	A description of all covariates tested
☐	☒	A description of any assumptions or corrections, such as tests of normality and adjustment for multiple comparisons
☐	☒	A full description of the statistical parameters including central tendency (e.g. means) or other basic estimates (e.g. regression coefficient) AND variation (e.g. standard deviation) or associated estimates of uncertainty (e.g. confidence intervals)
☒	☐	For null hypothesis testing, the test statistic (e.g. *F*, *t*, *r*) with confidence intervals, effect sizes, degrees of freedom and *P* value noted<br>*Give P values as exact values whenever suitable.*
☒	☐	For Bayesian analysis, information on the choice of priors and Markov chain Monte Carlo settings
☐	☒	For hierarchical and complex designs, identification of the appropriate level for tests and full reporting of outcomes
☒	☐	Estimates of effect sizes (e.g. Cohen's *d*, Pearson's *r*), indicating how they were calculated

Our web collection on statistics for biologists contains articles on many of the points above.

Software and code

Policy information about availability of computer code

Data collection	Cytometry data were acquired using the Helios cytometer. Cytometry data were processed as FCS files in Cytobank v10.3 (Beckmann Coulter), including manual gating. Gene expression data were used from publicly available datasets.
Data analysis	FCS files were exported to CSV files with further analysis undertake in the R environment for statistical computing (v.4.4.1 and earlier iterations and standard packages within tidyverse and base R). Code for the complex multi-omic factor analysis using the MOFA2 package is available at https://github.com/michaeljamescarter/SIFIC (DOI 10.5281/zenodo.12790924). Differential gene expression was undertaken in R (v.4.4.1 and earlier iterations) using the DESeq2 package. Gene set enrichment analyses were implemented in R (v.4.4.1 and earlier iterations) using fgsea (v.1.3) and the C7 immunologic signature gene sets available from as part of the Human MSigDB Collections (https://www.gsea-msigdb.org/gsea/msigdb/collections.jsp). We used Gene Ontology (GO) enrichment analysis. Code for the bioinformatic analyses of mass cytometry data is available at: https://github.com/michaeljamescarter/SIFIC. Code for the analysis of RNA-seq data is available at: https://github.com/PIDBG/misc_transcriptomic_signature29. A permanent repository for the code is at the DOI 10.5281/zenodo.12790924.

For manuscripts utilizing custom algorithms or software that are central to the research but not yet described in published literature, software must be made available to editors and reviewers. We strongly encourage code deposition in a community repository (e.g. GitHub). See the Nature Portfolio guidelines for submitting code & software for further information.

Data

Policy information about availability of data

All manuscripts must include a data availability statement. This statement should provide the following information, where applicable:

- Accession codes, unique identifiers, or web links for publicly available datasets
- A description of any restrictions on data availability
- For clinical datasets or third party data, please ensure that the statement adheres to our policy

Source data are provided with this paper. The gene counts and patient metadata for the Transcriptomic Cohort are available at ArrayExpress under accession code E-MTAB-11671 (https://www.ebi.ac.uk/biostudies/arrayexpress/studies/E-MTAB-11671). The merged and normalized dataset used for the analysis of the discovery dataset is available in ArrayExpress under accession code E-MTAB-12793 (https://www.ebi.ac.uk/biostudies/arrayexpress/studies/E-MTAB-12793). The raw FCS files for Supplementary Figure 10 are available on ImmPort (https://immport.niaid.nih.gov/) under accession code SDY2735. Other cytometry data are available under restricted access due to ongoing secondary analyses. Access can be obtained by contacting Dr Richard Ellis (Head of Advanced Cytometry Platform, King's College London; richard.ellis@kcl.ac.uk) with a brief analysis plan.

Research involving human participants, their data, or biological material

Policy information about studies with human participants or human data. See also policy information about sex, gender (identity/presentation), and sexual orientation and race, ethnicity and racism.

Reporting on sex and gender	Sex was not included in the study design and was self/parental-reported as sex at birth.
Reporting on race, ethnicity, or other socially relevant groupings	Ethnicity was self-reported by participating families on case record forms.
Population characteristics	Please see Table 1 of the Article for full details.
Recruitment	Eligibility criteria were suspected infection or inflammatory disease and weight ≥2.5 kg. Patients admitted to the Pediatric Intensive Care Unit were prioritized for recruitment. Samples from children in the cohort were selected for analysis if they were obtained from children acutely unwell with either MIS-C (as per WHO definition), COVID-19 pneumonitis (as per WHO definition), clinically diagnosed KD (as per American heart association guidelines, with or without SARS-CoV-2 IgG antibody), SVI, SBI, or a clinically diagnosed inflammatory disease. Age and other demographic data were not used in recruitment to the study.
Ethics oversight	The Derivation cohort were recruited to the observational study, DIAMONDS Search Study (Diagnosis and Management of Febrile Illness using RNA Personalised Molecular Signature Diagnosis; https://www.diamonds2020.eu) under UK HRA approval 20/HRA/1714 following informed written parental consent (children under 16 years of age) or informed written consent with parental assent (young people 16 to 18 years of age). The IRAS ID is 278651. The Transcriptomic cohort were recruited to the observational studies EUCLIDS (European Union Childhood Life-Threatening Infectious Disease Study, UK HRA approval 16/LO/1684), PERFORM (Personalised Risk Assessment in Febrile Illness to Optimise Real-Life Management across the European Union, UK HRA approval 11/LO/1982) and the DIAMONDS Search Study. Participants were not compensated.

Note that full information on the approval of the study protocol must also be provided in the manuscript.

Field-specific reporting

Please select the one below that is the best fit for your research. If you are not sure, read the appropriate sections before making your selection.

☒ Life sciences ☐ Behavioural & social sciences ☐ Ecological, evolutionary & environmental sciences

For a reference copy of the document with all sections, see nature.com/documents/nr-reporting-summary-flat.pdf

Life sciences study design

All studies must disclose on these points even when the disclosure is negative.

Sample size	Derivation cohort: 137 patients and 15 controls. Transcriptomic cohort: 372 children. Healthy adult COVID-19 vaccinated controls: 27 adults. Sample size was not pre-determined to the descriptive cohort study. Due to limited sample size, the statistical significance reported should only be interpreted as indicative of the direction of change in biological signals.
Data exclusions	One patient on whom mass cytometry data were acquired was excluded from the analysis due to incomplete clinical data. All other data are included within the paper and Source Data files.
Replication	Standardized sampling, storage and laboratory techniques were used for all patient samples. Batch effects were minimized by the random allocation of samples for processing on sequential days. Flow cytometry standard (FCS) files were then normalized with EQ beads for comparison between samples. Files were then concatenated using the CYTOF software (Fluidigm). We used CD45 labels ("barcodes") to enable pooling of six-plexed samples and subsequently debarcoding of samples from FCS files based on positive then negative selection by CD45 barcodes (BD, version 10.8.1). FCS files of debarcoded samples were then imported into CytofClean (https://rdrr.io/github/JimboMahoney/cytofclean/man/cytofclean.html) on R41 for further clean up and auto-gating based on the Gaussian parameters (Event

Length, Centre, Offset, Residual and Width). To normalize and correct for intra-experiment variability a whole blood technical control was run together with all the batches in each experiment and used for normalization using the FlowJo plugin Cytonorm.

For the supernatant cytokine assays measurements were done in duplicate and the mean of the two values was used. Principle component analyses (PCA) were performed to detect any batch effects as the samples were run across 20 plates.

Randomization

Participants were not randomly allocated into different groups. Disease group allocation was by predefined, internationally recognised, clinical diagnostic categories.

Blinding

Investigators were blinded to disease group and other participant details during the acquisition of single cell data. Investigators were otherwise not blinded to disease group and other participant details.

Reporting for specific materials, systems and methods

We require information from authors about some types of materials, experimental systems and methods used in many studies. Here, indicate whether each material, system or method listed is relevant to your study. If you are not sure if a list item applies to your research, read the appropriate section before selecting a response.

Materials & experimental systems

n/a	Involved in the study	
☐	☒	Antibodies
☒	☐	Eukaryotic cell lines
☒	☐	Palaeontology and archaeology
☒	☐	Animals and other organisms
☐	☒	Clinical data
☒	☐	Dual use research of concern
☒	☐	Plants

Methods

n/a	Involved in the study	
☒	☐	ChIP-seq
☐	☒	Flow cytometry
☒	☐	MRI-based neuroimaging

Antibodies

Antibodies used

Target Clone Metal tag Manufacturer Catalogue number Volume (µl) per 6 samples
CD45 HI30 106Cd Fluidigm 3106001B 2
CD45 HI30 110Cd Fluidigm 3110001B 2
CD45 HI30 198Pt Fluidigm 3198001B 2
CD45 HI30 89Y Fluidigm 3089003B 2
CD3 UCHT1 111Cd MaxPar Ready/GSTT 300443 2
CD4 RPA-T4 113Cd MaxPar Ready/GSTT 300541 2
CD25 2A3 169Tm Fluidigm 3169003B 2
CD127/IL-7Ra A019D5 176Yb Fluidigm 3176004B 2
CD45RO UCHL1 149Sm Fluidigm 3149001B 2
HLA-DR G46- 6 151Eu Fluidigm 3151023B 2
CD197/CCR7 G043H7 167Er Fluidigm 3167009A 2
CD45RA HI100 155Gd Fluidigm 3155011B 2
CD38 HB-7 HIT2 161Dy MaxPar Ready/GSTT 303535 2
CD8a RPA-T8 116Cd MaxPar Ready/GSTT 301002 2
TCRgd 11F2 152Sm Fluidigm 3152008B 2
CD19 HIB19 112Cd MaxPar Ready/GSTT 302247 2
CD14 RMO52 M5E2 114Cd MaxPar Ready/GSTT 301843 1
CD15 W6D3 142Nd Fluidigm 3144019B 2
CD16 3G8 209Bi Fluidigm 3209002B 2
CD20 2H7 171Yb Fluidigm 3171012B 2
CD11c Bu15 159Tb Fluidigm 3159001B 2
CD27 L128 162Dy Fluidigm 3162009B 2
CD5 UCHT2 141Pr MaxPar Ready/GSTT 300627 2
CD64 10.1 116Cd Fluidigm 305029 2
CD278/ICOS C398.4A 143Nd Fluidigm 3143025B 2
CD11b/Mac-1 ICRF44 144Nd Fluidigm 3144001B 2
CD223/LAG- 3 11C3C65 150Nd Fluidigm 3150030B 2
CD10 HI10a 156Gd Fluidigm 3156001B 2
CD134/OX40 ACT35 158Gd Fluidigm 3158012B 2
CD95/Fas DX2 163Dy MaxPar Ready/GSTT 305631 2
CD161 HP- 3G10 164Dy Fluidigm 3164009B 2
CD206/MMR 15-2 168Er Fluidigm 3168008B 2
CD279/PD-1 EH12.2H7 174Yb Fluidigm 3174020B 2
CD28 CD28.2 160Gd Fluidigm 3160003B 2

IL-6 MQ2- 13A5 154Sm Fluidigm 3154011B 3
IL-17A BL168 148Nd Fluidigm 3148008B 3
IFNg B27 165Ho Fluidigm 3165002B 3
TNFa MAb11 146Nd Fluidigm 3146010B 3
IL-2 MQ1-17H12 145Nd MaxPar Ready/GSTT 500339 3
CD152/CTLA-4 MQ1-17H12 170Er Fluidigm 3170005B 3
pStat5 [Y694] (47) 147Sm Fluidigm 3147012A 5
pStat1 [Y701] (4a) 153Eu Fluidigm 3153005A 5
pS6 [S235/S236] (N7- 548) 175Lu Fluidigm 3175009A 5
pNFkBp65 [S529] (K10x) 166Er Fluidigm 3166006A 5
IFNg B27 165Ho Fluidigm
TNFa MAb11 146Nd Fluidigm
IL-2 MQ1-17H12 145Nd MaxPar Ready/GSTT
CD152/CTLA-4 MQ1-17H12 170Er Fluidigm
pStat5 [Y694] (47) 147Sm Fluidigm
pStat1 [Y701] (4a) 153Eu Fluidigm
pS6 [S235/S236] (N7- 548) 175Lu Fluidigm
pNFkBp65 [S529] (K10x) 166Er Fluidigm

Validation

All antibodies were either from Fluidigm (now Standard Biotool) pre-conjugated to heavy metals, or MaxPar Ready antibodies from Biolegend that were conjugated to heavy metals at the Guy's and St Thomas' NHS Trust (GSTT) Advanced Flow Cytometry Facility. Fluidigm (Standard Biotools) are optimised for Cytof and ready to use. MaxPar Ready antibodies are CyTOF-validated purified antibodies for metal labeling are ready purified antibodies are optimized and ready-to-use. All antibodies underwent extensive further optimisation for the bespoke panel. The staining protocol is available on ImmPort under the accession code SDY2735.

Clinical data

Policy information about clinical studies
All manuscripts should comply with the ICMJEguidelines for publication of clinical research and a completedCONSORT checklist must be included with all submissions.

Clinical trial registration

This descriptive cohort received UK HRA approval (20/HRA/1714). The IRAS ID is 278651.

Study protocol

The study protocol is the same as the Diamonds Study (https://www.diamonds2020.eu/about/), which was the study to which the patients we report were co-recruited.

Data collection

The study began recruitment on 28th April 2020. Patients included in this cohort were recruited from August 2020 up until September 2021. Participants were recruited from the Paediatric Intensive Care and Paediatric Wards at Evelina London Children's Hospital, Imperial College Healthcare NHS Trust, and Greenwich and Lewisham NHS Trust all in London, UK. Details of the site of care at each stage of sampling are noted in Supplementary Figure 1.

Outcomes

This is a descriptive cohort study. The predefined outcomes of interest were immune cell features (analysed by mass cytometry) at acute presentation, defervescence and convalescence; response of immune cells using SARS-CoV-2 antigen and mitogen; differential gene expression. These primary outcomes were based on published literature in severe febrile illnesses in children.

Plants

Seed stocks

Not applicable.

Novel plant genotypes

Not applicable.

Authentication

Not applicable.

Flow Cytometry

Plots

Confirm that:

- [ ] The axis labels state the marker and fluorochrome used (e.g. CD4-FITC).
- [ ] The axis scales are clearly visible. Include numbers along axes only for bottom left plot of group (a 'group' is an analysis of identical markers).
- [ ] All plots are contour plots with outliers or pseudocolor plots.
- [ ] A numerical value for number of cells or percentage (with statistics) is provided.

Methodology

Sample preparation	Samples of 500 µl of whole blood from patients were pipetted directly into 500 µl of Cytodelics cell stabilizing solution (Cytodelics ab, Sweden) and stored within 30 min at -80ºC. Following storage, samples were initially processed by rapid thawing in a water bath at 37ºC for 1–2 min, fixing in 2500 µl Cytodelics fixation buffer with the addition of 50 µg EDTA (250 µl of 0.5 mM / 0.02% solution; Thermofisher, MA, USA) at 20ºC for 15 min before lysis of red cells using 5 ml Cytodelics lysis buffer for 20 min. Samples were subsequently washed (10 ml Cytodelics wash buffer) twice with centrifugation set at 1000 g for 5 min at 20ºC, before transfer from a 15 ml Falcon tube to a microcentrifuge tube for further processing. Unless otherwise stated, all further centrifugation steps were at 1000 g for 5 min at 20ºC. Cells were centrifuged and then resuspended in 50 µl Fc receptor blocking solution (45 µl cell staining buffer (CSB) / 5 U heparin / 5 µl FcX Trustain, Biolegend, CA, USA) and incubated for 10 min at 20 ºC. Further details of the staining protocol are available on ImmPort under accession code SDY2735.
Instrument	A Fluidigm Helios Mass Cytometer for mass cytometry at Guys' and St Thomas' NHS Trust Advanced Cytometry Facility. BD LSR Fortessa flow cytometer was used for the acquisition of multi-plexed ELISA data for cytokines in stimulated cell supernatant.
Software	For mass cytometry data, flow cytometry standard (FCS) files were normalized with EQ beads for comparison between samples. Files were then concatenated using the CYTOF software (Fluidigm). We used CD45 labels ("barcodes") to enable pooling of six-plexed samples and subsequently debarcoding of samples from FCS files based on positive then negative selection by CD45 barcodes (BD, version 10.8.1). FCS files of debarcoded samples were then imported into CytofClean (https://rdrr.io/github/JimboMahoney/cytofclean/man/cytofclean.html) on R41 for further clean up and auto-gating based on the Gaussian parameters (Event Length, Centre, Offset, Residual and Width). To normalize and correct for intra-experiment variability a whole blood technical control was run together with all the batches in each experiment and used for normalization using the FlowJo plugin Cytonorm. Samples were then imported on the online platform Cytobank for further informatic analysis (https://www.cytobank.org).<br><br>For supernatant cytokine data, the LEGENDplex (Biolegend) guide was followed to set up the PMT voltages with the set-up beads using the FACSDiva software (Version 6.0). The LEGENDplex Data Analysis Software Suite (Version 8) was used for initial quality control checks. The automated gating of bead population was reviewed and manually adjusted for any misclassifications.
Cell population abundance	All cell population abundance data are included as Source Data files with this paper for all gated cell populations.
Gating strategy	The gating strategy for the paper is in Supplementary Figure 3. FSC and SSC are not parameters measured on mass cytometry or on multiplexed ELISA data acquired using the Fortessa flow cytometer.

- [x] Tick this box to confirm that a figure exemplifying the gating strategy is provided in the Supplementary Information.
